# Supplementary material for: Thermally stable single atom Pt/m-Al2O3 for selective hydrogenation and CO oxidation
Source: Nat Commun. 2017 Jul 27;8:16100. doi: 10.1038/ncomms16100 (PMC5537564; doi:10.1038/ncomms16100)
Supplement: Supplementary Information [file ncomms16100-s1.pdf]

File Name: Supplementary Information
Description: Supplementary Figures, Supplementary Tables, Supplementary Methods and Supplementary References

File Name: Peer Review File
Description:

Supplementary methods

Materials

Chloroplatinic acid ($H_2PtCl_6.6H_2O$), aluminum isopropoxide ($C_9H_{21}AlO_3$), $(EO)_{20}(PO)_{70}(EO)_{20}$ triblock copolymer (Pluronic P123, Mn = 5800), *n*-hexane (≥ 99%) and commercial 5 wt% Pt/Al_2O_3 were obtained from Sigma-Aldrich. Ethanol (absolute) was from Fisher Scientific. Hydrochloric acid (37%) and nitric acid (67%) were supplied by Merk Pte. Ltd. All chemicals were used as received.

Catalysts synthesis

Synthesis of 0.2Pt/m-Al_2O_3-O_2-600 and 0.2Pt/m-Al_2O_3-O_2-800

The gel, prepared by the same process for the synthesis of the 0.2Pt/m-Al_2O_3-O_2 precursor, was calcined at 600 ^oC and 800 ^oC for 4 h at a heating rate of 1 ^oC min^{-1}. These two samples are named as 0.2Pt/m-Al_2O_3-O_2-600 and 0.2Pt/m-Al_2O_3-O_2-800, respectively.

Synthesis of 0.2Pt/p-Al_2O_3-O_2, 0.5Pt/p-Al_2O_3-O_2, and 2.0Pt/p-Al_2O_3-O_2

In a typical synthesis, 67% nitric acid (3.2 mL) and aluminum isopropoxide (4.08 g) were dissolved in ethanol (20 mL). Then, stoichiometric amounts of H_2PtCl_6 (0.2 wt%, 0.5 wt% and 2.0 wt% of Pt, respectively, compared with Al_2O_3 to be generated) dissolved in ethanol (0.0193 mol/L) were added to the above solution under stirring. The obtained mixture was covered with a PE film, stirred at room temperature for 48 h and was placed in an oven at 60 ^oC for ethanol evaporation for 72 h. The final gel was calcined at 400 ^oC for 4 h at a heating rate of 1 ^oC min^{-1}, and are named as 0.2Pt/p-Al_2O_3-O_2, 0.5Pt/p-Al_2O_3-O_2, and 2.0Pt/p-Al_2O_3-O_2, respectively.

Synthesis of 0.2Pt/p-Al_2O_3-H_2, 0.5Pt/p-Al_2O_3-H_2, and 2.0Pt/p-Al_2O_3-H_2

The samples (0.2Pt/p-Al_2O_3-O_2, 0.5Pt/p-Al_2O_3-O_2, and 2.0Pt/p-Al_2O_3-O_2) were reduced in 5% H_2/N_2 at 400 ^oC for 1 h at a heating rate of 5 ^oC min^{-1}, and are named as 0.2Pt/p-Al_2O_3-H_2, 0.5Pt/p-Al_2O_3-H_2, and 2.0Pt/p-Al_2O_3-H_2, respectively.

Synthesis of 0.2Pt/p-Al_2O_3-O_2-600 and 0.2Pt/p-Al_2O_3-O_2-800

The gel, prepared by the same process for synthesis of the 0.2Pt/p-Al_2O_3-O_2 precursor, was calcined at 600 ^oC and 800 ^oC for 4 h at a heating rate of 1 ^oC min^{-1}, and are named as 0.2Pt/p-Al_2O_3-O_2-600 and 0.2Pt/p-Al_2O_3-O_2-800, respectively.

Synthesis of mesoporous Al_2O_3

In a typical synthesis, Pluronic P123 (2.1 g) was dissolved in ethanol (20 mL) at room temperature. 67% nitric acid and aluminum isopropoxide (4.08 g) were dissolved in ethanol (20 mL). The two solvents were combined with vigorous stirring, and then the mixture was covered with a PE film and stirred at

room temperature for 48 h before placing in an oven at 60 ^{o}C for 72 h for ethanol evaporation. The gel obtained was calcined at 400 ^{o}C for 4 h at a heating rate of 1 ^{o}C min^{-1}, and is named as m-Al_2O_3.

Synthesis of Pt supported on m-Al_2O_3 using wet-impregnation method

Stoichiometric amounts of H_2PtCl_6 (0.2 wt% of Pt compared with m-Al_2O_3) dissolved in ethanol (0.0193 mol/L) was added to an ethanol solution (20 mL) containing 0.5 g m-Al_2O_3 under stirring at room temperature for 48 h and was placed in an oven at 60 ^{o}C for ethanol evaporation for 72 h. The final power was calcined at 400 ^{o}C for 4 h at a heating rate of 1 ^{o}C min^{-1}. Then, the sample was reduced in 5% H_2/N_2 at 400 ^{o}C for 1 h at a heating rate of 5 ^{o}C min^{-1} (denoted as 0.2Pt/m-Al_2O_3-imp).

Additional methods for characterizations

HRSTEM image analysis

Pt single atoms in the HAADF-STEM image are identified by searching the local intensity maxima using the algorithm reported by I. F. Sbalzarini etc.[1]. The positions of single atoms are further refined and their coordinates are extracted for the nearest neighbor distance analysis. Specifically, we found that by using a aperture size of ~ 1.6 Å, a cut-off score of 0 for the non-particle discrimination, and an accepted percentile of 0.01 ~ 0.05, single atoms can be well identified in most HRSTEM images. Those located and refined single atoms are carefully inspected to avoid spurious identification before proceeding to the distance analysis.

DRIFTS analysis

For 0.2Pt/m-Al_2O_3-O_2, 0.5Pt/m-Al_2O_3-O_2, and 2.0Pt/m-Al_2O_3-O_2, the catalysts were pretreated with 40 ml min^{-1} of air at 400 °C for 1 h. For 0.2Pt/m-Al_2O_3-H_2, 0.5Pt/m-Al_2O_3-H_2, and 2.0Pt/m-Al_2O_3-H_2, the catalysts were pretreated with 40 ml min^{-1} of 5% H_2/N_2 at 400 °C for 1 h. The CO adsorption on these samples was performed at 30 °C. 5% CO/Ar was introduced into the DRIFTS cell at a flow rate of 40 ml min^{-1}. After CO saturation, a N_2 purge at a flow rate of 40 ml min^{-1} was performed to remove gas phase CO from the DRIFTS cell. A temperature programmed desorption process was carried out to completely remove CO adsorption on Pt catalysts. All the spectra were recorded using 32 scans with a resolution of 4 cm^{-1}.

XAS measurement

Pt L_3-edge XAS spectra of catalysts and Pt foil were recorded at the BL01B1 beamline at the SPring-8 (Japan Synchrotron Radiation Research Institute, Hyogo, Japan) in the transmission mode at ambient temperature. Data analysis was carried out with Athena and Artemis included in the Demeter package. For curve fitting analysis of EXAFS spectra, each theoretical scattering path was generated with FEFF 6.0 L. The k^3-weighted EXAFS oscillation in the range of 3.0–12 Å^{-1} was Fourier transformed.

TPR, TPD, H_2-O_2 and CO titration analysis

For TPR analysis, 0.2Pt/m-Al_2O_3-O_2 (500 mg), 0.5Pt/m-Al_2O_3-O_2 (200 mg), and 2.0Pt/m-Al_2O_3-O_2 (50 mg) catalysts were pre-treated in N_2 at 150 °C for 1 h before cooling down to 50 °C. Afterwards, the flow gas was switched to 5% H_2/N_2 (80 mL min^{-1}) for 1 h at this temperature, and then TPR was performed over the samples with the temperature increasing from 50 °C to 600 °C at a speed of 10 °C min^{-1}. 2nd and 3rd rounds of TPR were collected with the same temperature program, without sample exposure to air. For TPD analysis of 0.2Pt/m-Al_2O_3-O_2 (500 mg) before treatment, the TPD signal was collected in He (80 mL min^{-1}) flow from 30 °C to 400 °C at a speed of 10 °C min^{-1}. For another run, the sample was treated in He flow at 150 °C for 1 h to remove the adsorbed water. For H_2-O_2 titration experiment a certain amount of precatalysts (0.2Pt/m-Al_2O_3-O_2, 0.5Pt/m-Al_2O_3-O_2, or 2.0Pt/m-Al_2O_3-O_2, detailed amounts were indicated in related figures) pre-treated at 100 °C with flowing pure air for 30 min to remove the hydrogen atoms absorbed on Pt atoms. After that, N_2 was used as carried gas at 80 mL min^{-1}, and the successive doses of H_2 gas were subsequently introduced into N_2 stream by means of a calibrated injection valve (159 μL 20% H_2/N_2 pulse^{-1}) at 100 °C. The titration will end when the intensities of three peaks in a row keeps constant. In the second round of titration, the samples were first reduced at 400 °C in 5% H_2/N_2 (80 mL min^{-1}) for 1 h, cooled to 100 °C with gas switched to pure air for 30 min to remove the hydrogen atoms absorbed on Pt atoms. After that, N_2 was used as carried gas at 80 mL min^{-1}, and the successive doses of H_2 gas were subsequently introduced into N_2 stream by means of a calibrated injection valve (159 μL 20% H_2/N_2 pulse^{-1}) at 100 °C. The titration will end when the intensities of three peaks in a row keeps constant. For CO titration experiment, a certain amount of catalyst (detailed amounts were indicated in related figures) was pre-treated at 400 °C, then cooled to 30 °C. After that, helium (He) was used as carried gas at 80 mL min^{-1}, and successive doses of CO gas were introduced into He stream by a calibrated injection valve (159 μL 5% CO/He pulse^{-1}) at 30 °C. The titration ended after 16 injections. In the second round titration, the samples were first reduced at 400°C in 5% H_2/N_2 (80 mL min^{-1}) for 1 h, cooled to 30 °C. After that, helium (He) was used as a carry gas at 80 mL min^{-1}, and then successive doses of CO gas were introduced into He stream by a calibrated injection valve (159 μL 5% CO/He pulse^{-1}) at 30 °C. The titration ended after 16 injections.

Small-angle Angle X-ray Diffraction analysis

Small-angle XRD measurements were conducted on a SmartLab diffractometer (Rigaku Corporation) equipped with a 9 kW rotating anode Cu source at 40 kV and 40 mA, from 0.6 to 5° (0.02° s^{-1}), Incident slit, 0.500 mm.

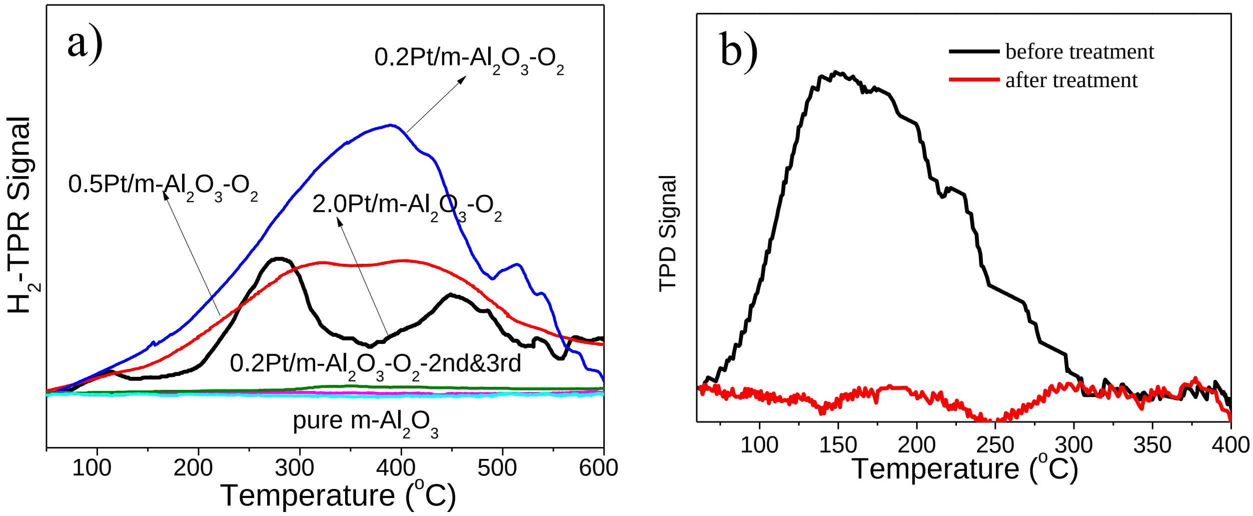

Supplementary Figure 1 | (a) TPR profiles for Pt/m-Al_2O_3. Reduction conditions: 5% H_2/N_2, 80 mL min^{-1}, 10 °C min^{-1} heating rate (50–600 °C). Before TPR, the samples were heated at 150 °C for one hour (N_2), cooled down to 50 °C (N_2) and stabilized (5% H_2/N_2) at 50 °C for 1 h. **(b) TPD profiles for 0.2Pt/m-Al_2O_3-O_2 before and after 150 °C treatment for one hour.** This suggests that 150 °C pre-treating is sufficient to prevent the interference of adsorbed H_2O on Al_2O_3 to TPR signals.

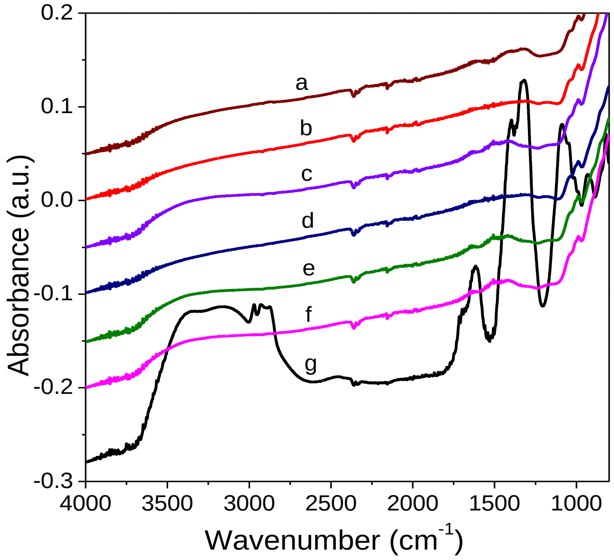

Supplementary Figure 2 | FT-IR spectra of some samples after thermal treatment. (**a**) 0.2Pt/m-Al_2O_3-H_2, (**b**) 0.2Pt/m-Al_2O_3-O_2, (**c**) 0.5Pt/m-Al_2O_3-H_2, (**d**) 0.5Pt/m-Al_2O_3-O_2, (**e**) 2.0Pt/m-Al_2O_3-H_2, (**f**) 2.0Pt/m-Al_2O_3-O_2, and (**g**) the gel for preparing sample 0.2Pt/m-Al_2O_3-O_2 before calcination. Compared with the gel before calcination, the samples exhibited similar FT-IR spectra with no detectable C-H stretching bands, indicating complete removal of ligands and templates after calcination.

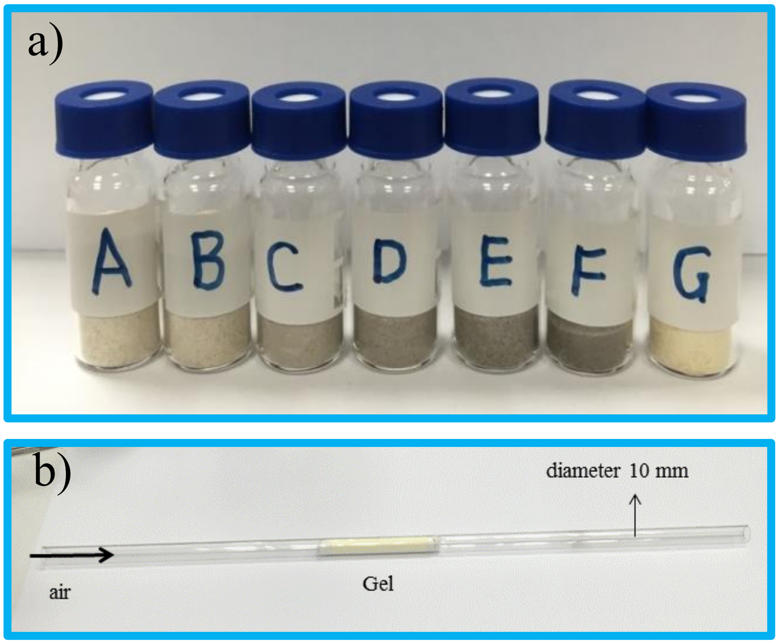

Supplementary Figure 3 | Photographs of samples. (**a**) (**A**) 0.2Pt/m-Al_2O_3-O_2, (**B**) 0.2Pt/m-Al_2O_3-H_2, (**C**) 0.5Pt/m-Al_2O_3-O_2, (**D**) 0.5Pt/m-Al_2O_3-H_2, (**E**) 2.0Pt/m-Al_2O_3-O_2, (**F**) 2.0Pt/m-Al_2O_3-H_2, and (**G**) pure m-Al_2O_3, and (**b**) the quartz reactor tube fed with gel before calcination. The color of the samples, to some extent, is an indicator of the existence of Pt nanoparticles in the material. The 0.5 and 2.0 wt% samples exhibited dark color suggesting the presence of Pt nanoparticles. The 0.2 wt% samples, although slightly darker than pure m-Al_2O_3, are much lighter than higher loading samples. Indeed, the presence of Pt NPs in 0.2Pt/m-Al_2O_3-O_2 and 0.2Pt/m-Al_2O_3-H_2 are below the detection limit of X-ray absorption spectroscopy (*vide infra*).

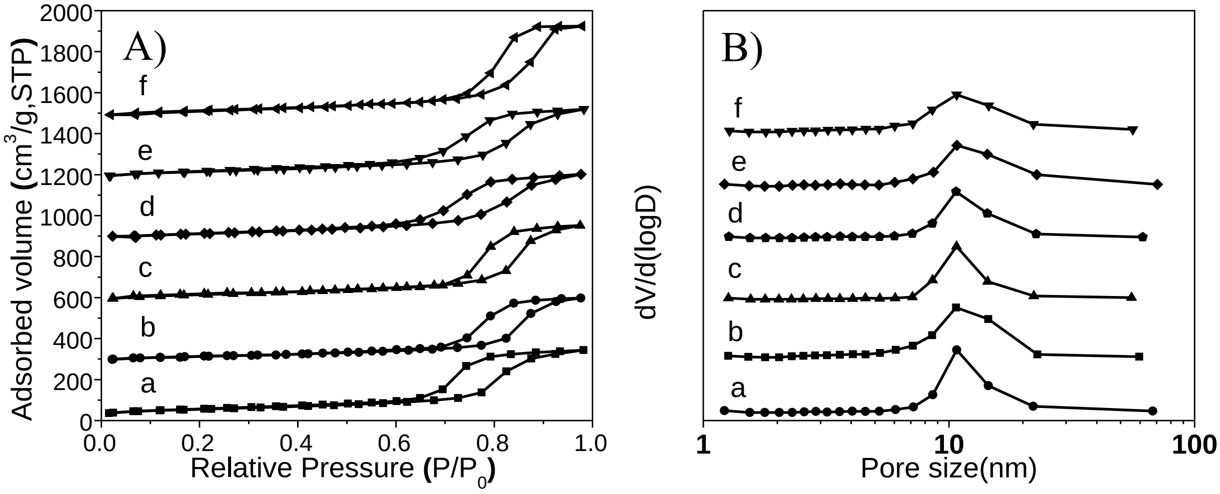

Supplementary Figure 4 | (A) N_2 adsorption-desorption isotherms, and (B) their PSD curves. (**a**) 0.2Pt/m-Al_2O_3-O_2, (**b**) 0.2Pt/m-Al_2O_3-H_2, (**c**) 0.5Pt/m-Al_2O_3-O_2, (**d**) 0.5Pt/m-Al_2O_3-H_2, (**e**) 2.0Pt/m-Al_2O_3-O_2, and (**f**) 2.0Pt/m-Al_2O_3-H_2 (samples **b,c,d,e,f** were vertically shifted 300, 600, 900, 1200 and

1500 cm^3 g^{-1}, respectively). The surface area and pore volume of these samples are summarized in Supplementary Table 3.

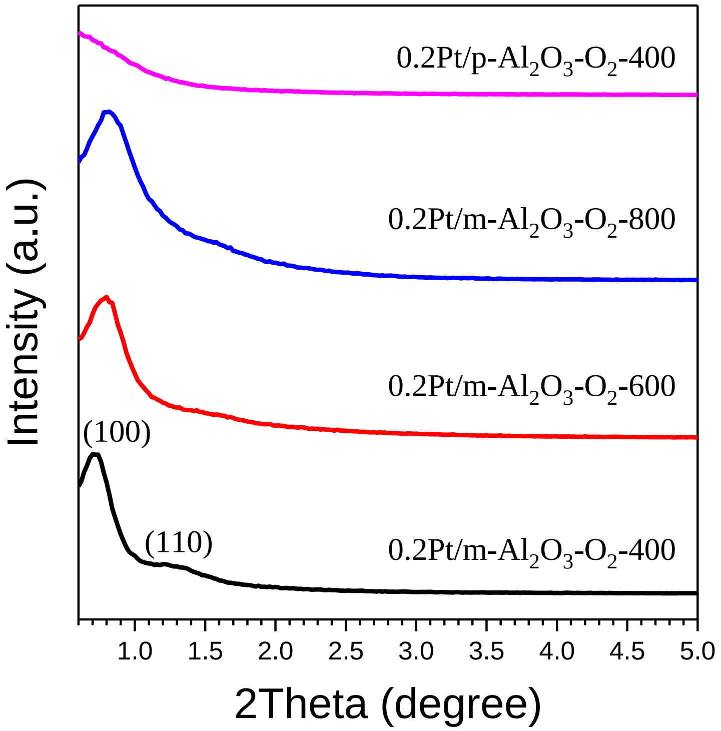

Supplementary Figure 5 | Small-angle XRD patterns of 0.2Pt/m-Al_2O_3-O_2 and 0.2Pt/p-Al_2O_3-O_2 after high temperature treatment. Evidence of the presence of hexagonally ordered mesopores (p6mm symmetry) for the 0.2Pt/m-Al_2O_3 samples is provided by small angle XRD patterns. The XRD patterns show two reflections, (100) and (110), for the m-Al_2O_3 samples calcined at 400, 600, and 800 °C; the d-spacing values corresponding to (100) reflections are 11.1 nm for 0.2Pt/m-Al_2O_3-O_2-400, 10.0 nm for 0.2Pt/m-Al_2O_3-O_2-600, and 9.7 nm for 0.2Pt/m-Al_2O_3-O_2-800. In sharp contrast, the XRD spectrum for 0.2Pt/p-Al_2O_3-O_2-400 is featureless, indicating the lack of ordered porosity.

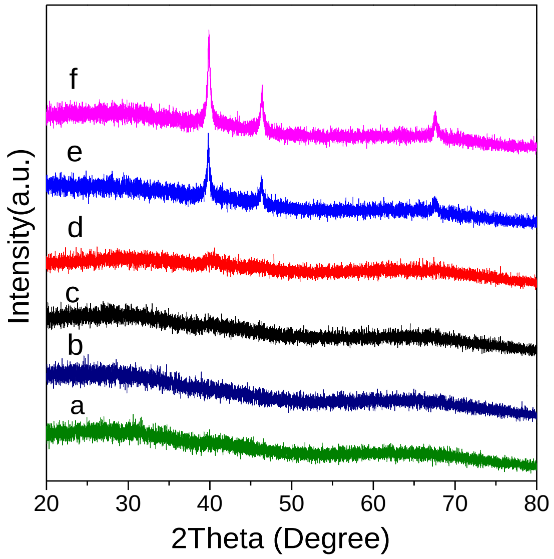

Supplementary Figure 6 | XRD patterns of typical samples. (**a**) 0.2Pt/m-Al_2O_3-O_2, (**b**) 0.2Pt/m-Al_2O_3-H_2, (**c**) 0.5Pt/m-Al_2O_3-O_2, (**d**) 0.5Pt/m-Al_2O_3-H_2, (**e**) 2.0Pt/m-Al_2O_3-O_2 and (**f**) 2.0Pt/m-Al_2O_3-H_2. XRD patterns suggest the presence of nanoparticles on 2.0Pt/m-Al_2O_3-O_2 and 2.0Pt/m-Al_2O_3-H_2. No obvious Pt peaks were observed for 0.2Pt/m-Al_2O_3-O_2, 0.2Pt/m-Al_2O_3-H_2, 0.5Pt/m-Al_2O_3-O_2, 0.5Pt/m-Al_2O_3-H_2, indicating most Pt species are nanoclusters and/or single-atoms.

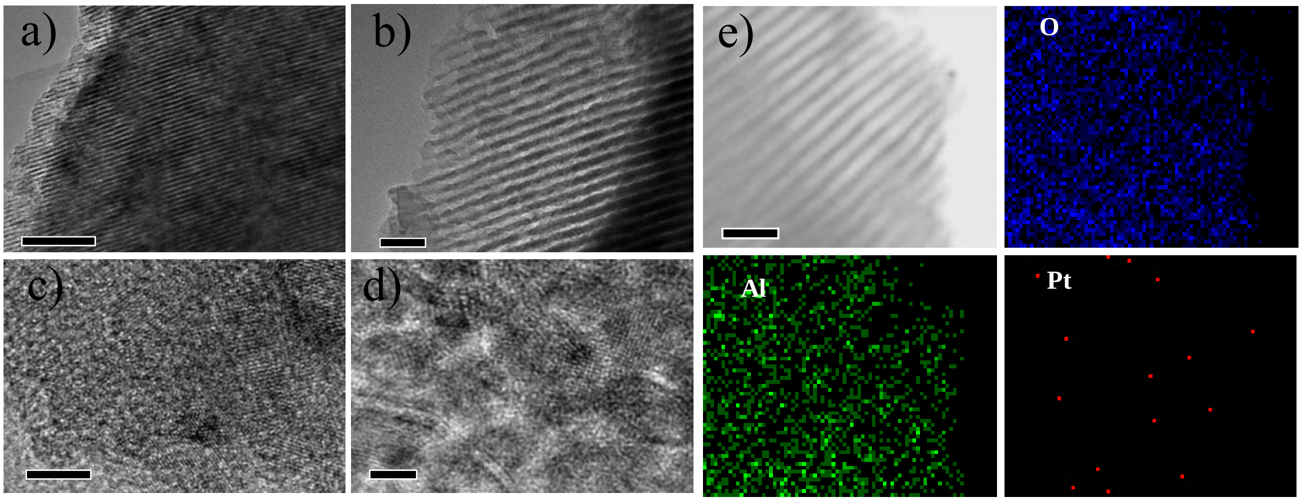

Supplementary Figure 7 | (**a-d**) TEM images and (**e**) TEM-EDS elemental mapping images for 0.2Pt/m-Al_2O_3-O_2. Scale bar, 100 nm **(a)**, 20 nm **(b)**, 5 nm **(c)**, 2 nm **(d)**, 20 nm **(e)**.

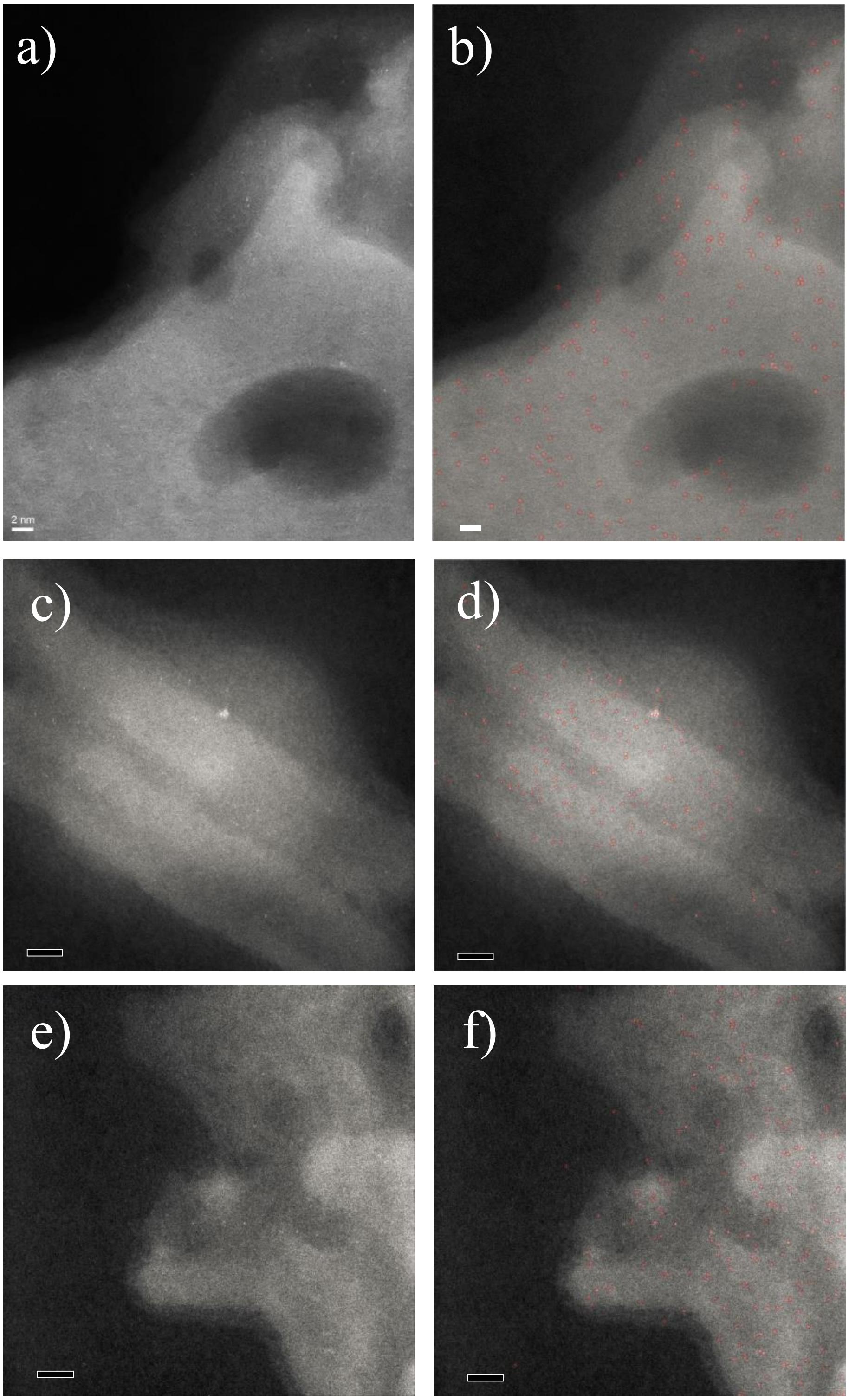

Supplementary Figure 8 | Additional HAADF-STEM images of 0.2Pt/m-Al_2O_3 catalysts. (**a**) 0.2Pt/m-Al_2O_3-H_2 and (**b**) the corresponding images with single Pt atom circled in red, (**c,e**) 0.2Pt/m-Al_2O_3-O_2 and (**d,f**) the corresponding images with single Pt atoms circled in red. Scale bar, 2 nm**.** These additional HAADF-STEM images indicate that Pt single-atoms are uniformly dispersed in m-Al_2O_3.

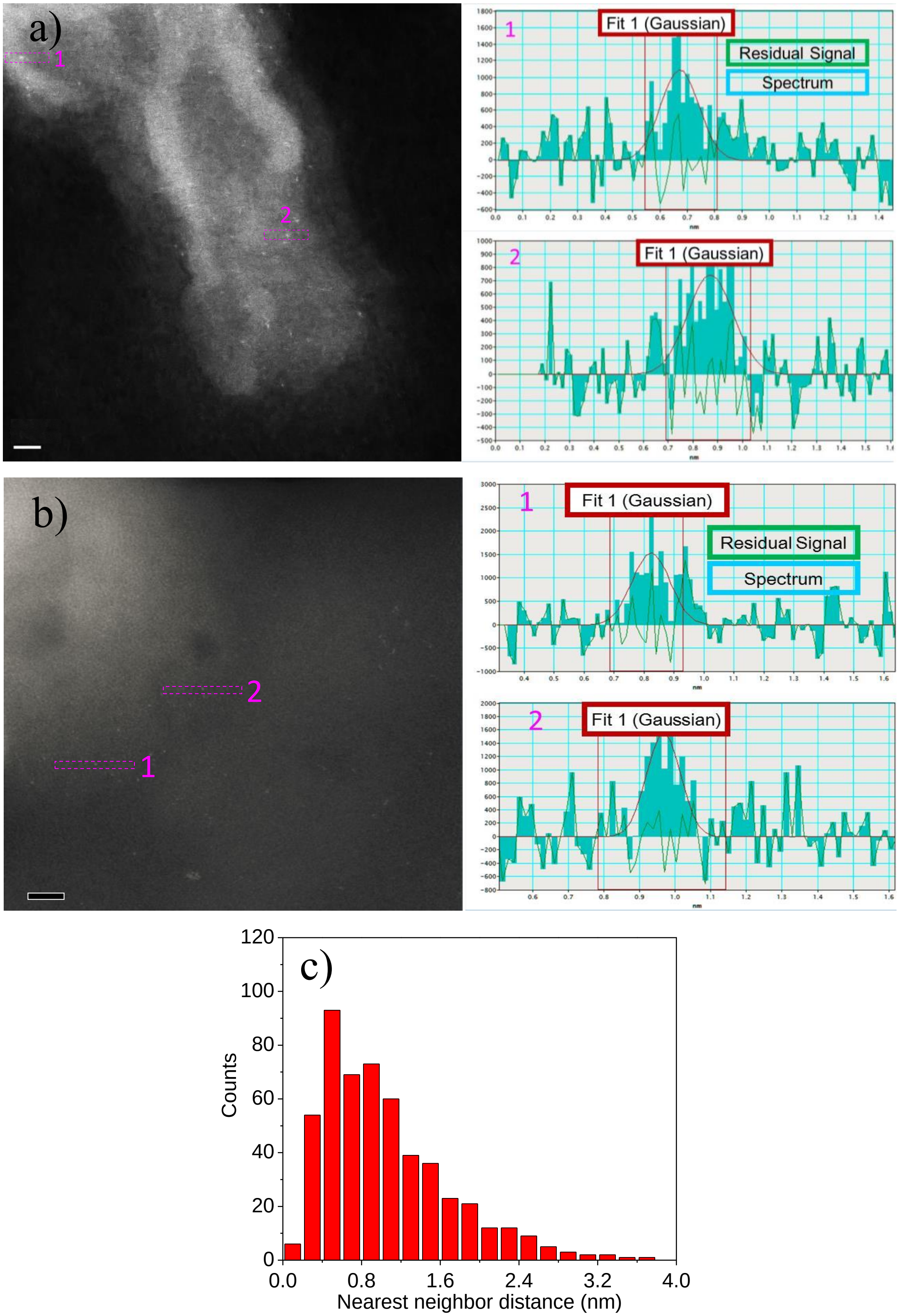

Supplementary Figure 9 | Additional HAADF-STEM image, and the normalized image intensities in the rectangle region directions 1 and 2 from the Pt atoms of samples (**a**) 0.2Pt/m-Al_2O_3-O_2 and (**b**) 0.2Pt/m-Al_2O_3-H_2, and (**c**) the number of atoms for the nearest neighbor distance from 525 single

atoms identified for 0.2Pt/m-Al_2O_3-O_2. Note that the distances between neighboring single atoms measured from HAADF-STEM are "projected distances". The true three-dimensional "inter-atom distances" should be larger. However, given that the HAADF-STEM images were taken from ultra-thin areas in the specimen to gain reasonable contrast of single atoms and that the image contrast is very sensitive to the vertical position of single atoms, the identified single atoms should not differ much in height. Therefore, it would not bring much error to approximate the "projected distances" as "inter-atom distances" in this specific case. Scale bar, 2 nm**.**

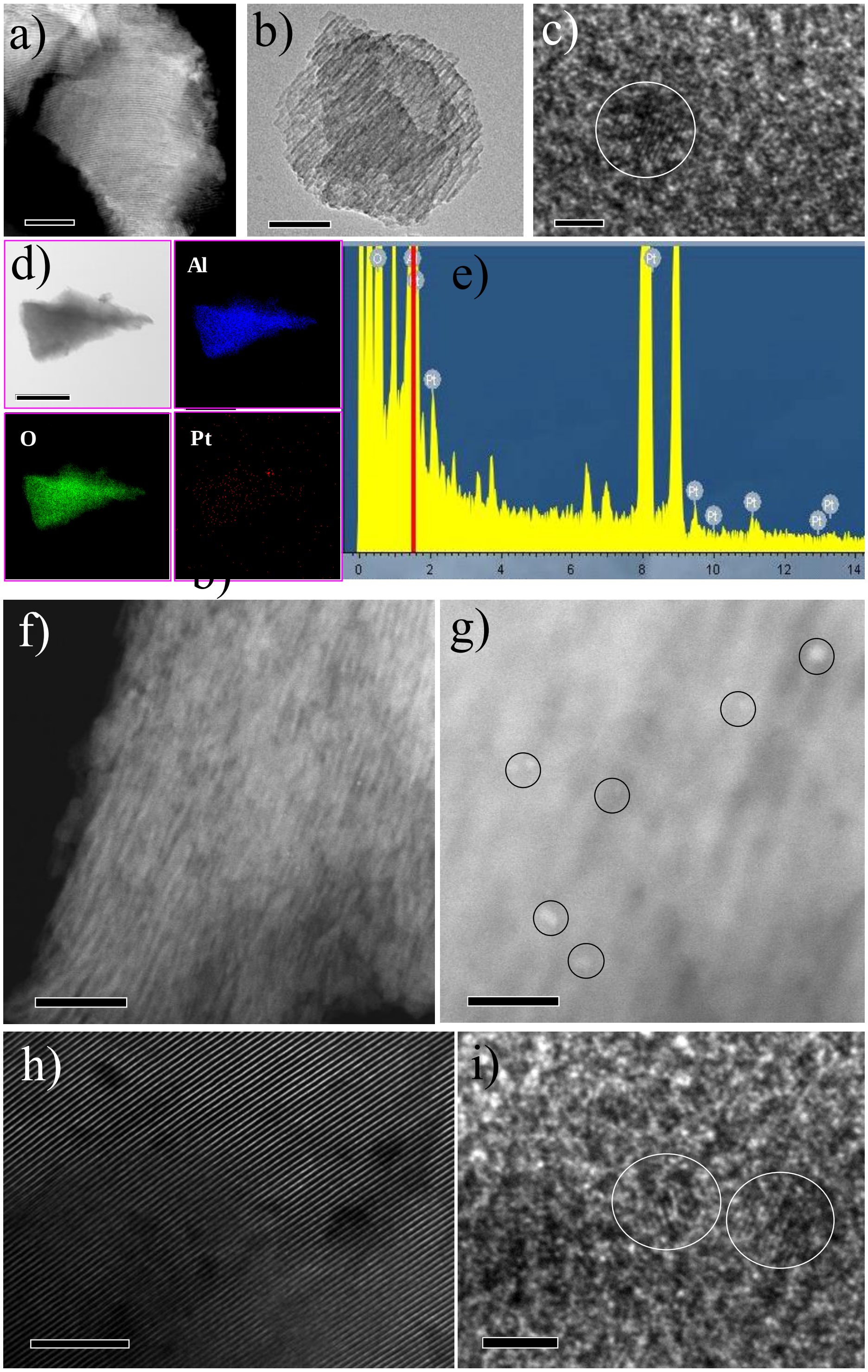

Supplementary Figure 10 | Characterizations of fresh 0.5Pt/m-Al_2O_3 catalyst. (**a**) HAADF-STEM image, (**b,c**) TEM images, (**d**) TEM elemental mapping images, (**e**) TEM-EDS image of 0.5Pt/m-Al_2O_3-O_2, (**f,g**) HAADF-STEM images and (**h,i**) TEM images of 0.5Pt/m-Al_2O_3-H_2. Scale bar, 200 nm **(a)**, 50 nm **(b)**, 2 nm **(c)**, 1 μm **(d)**, 100 nm **(f)**, 20 nm **(g)**, 200 nm **(h)**, 2 nm **(i)**. When Pt loading increased to 0.5 wt%, some Pt nanoclusters were formed. TEM elemental mapping and EDS image showed that the Pt species uniformly dispersed in the sample.

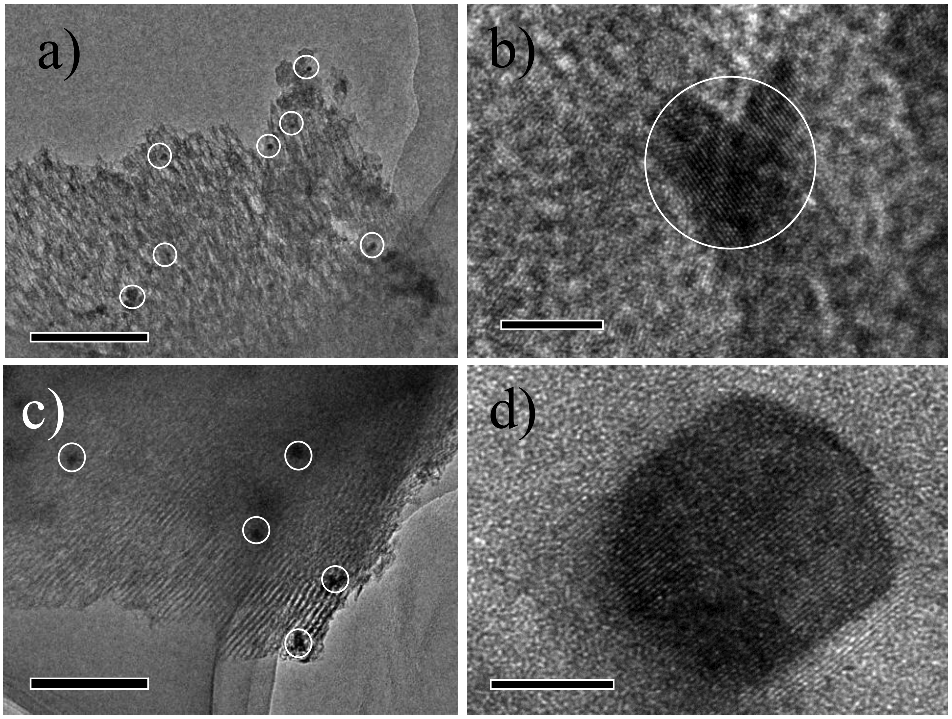

Supplementary Figure 11 | TEM images. (**a,b**) 2.0Pt/m-Al_2O_3-O_2 and (**c,d**) 2.0Pt/m-Al_2O_3-H_2. Scale bar, 200 nm **(a,c)**, 5 nm **(b,d)**. When the Pt loading increased to 2.0 wt%, Pt nanoparticles were clearly observed on 2.0Pt/m-Al_2O_3-O_2, and 2.0Pt/m-Al_2O_3-H_2.

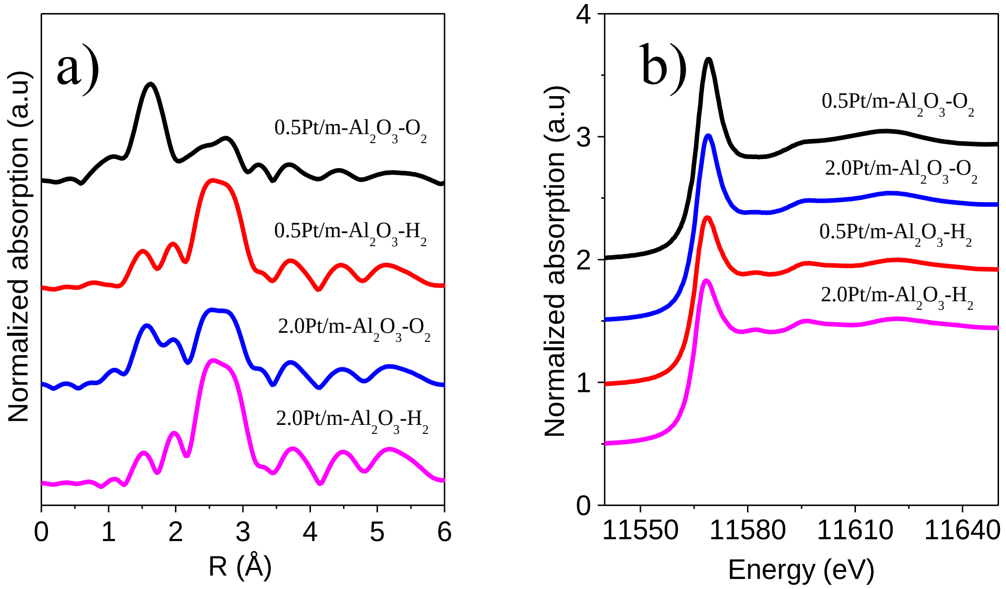

Supplementary Figure 12 | (**a**) The k^3-weighted Fourier transform spectra derived from EXAFS and (**b**) normalized XANES spectra at the Pt L_3-edge of 0.5Pt/m-Al_2O_3-O_2, 0.5Pt/m-Al_2O_3-H_2, 2.0Pt/m-Al_2O_3-O_2, and 2.0Pt/m-Al_2O_3-H_2.

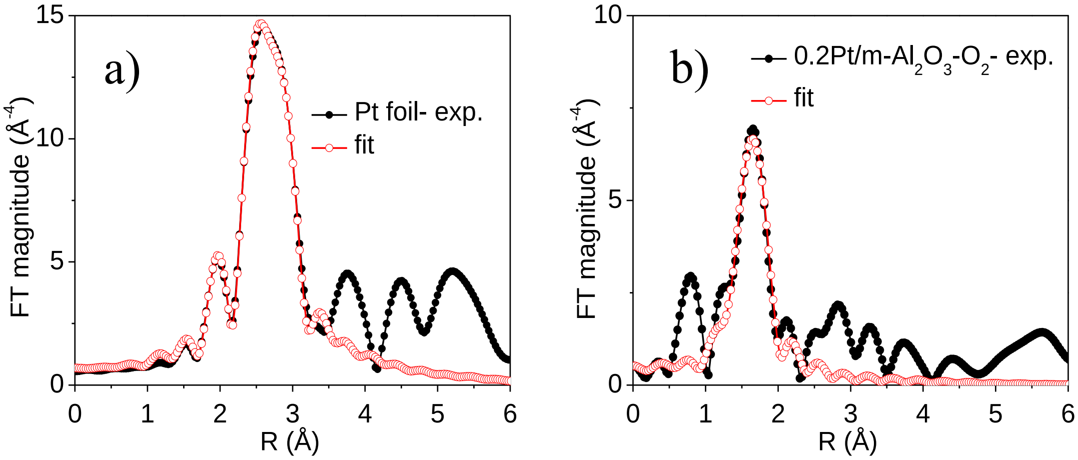

Supplementary Figure 13 | Comparison of FT-EXAFS curves between the experimental data and the fitting curve of the K-edge spectra. (**a**) Pt foil and (**b**) 0.2Pt/m-Al_2O_3-O_2.

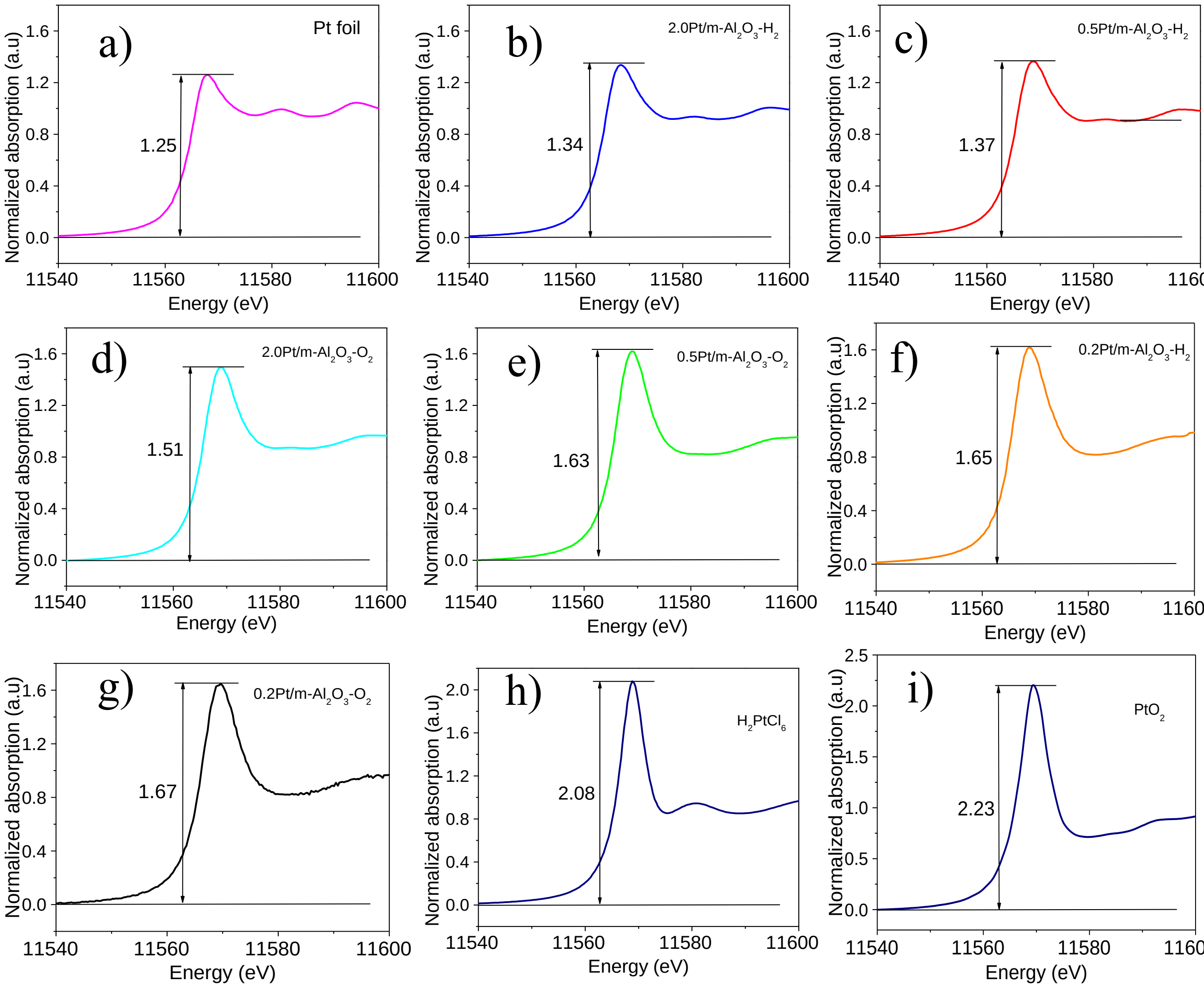

Supplementary Figure 14 | XANES spectra of Pt/m-Al_2O_3 catalysts and standard samples. (**a**) Pt foil, (**b**) 2.0Pt/m-Al_2O_3-H_2, (**c**) 0.5Pt/m-Al_2O_3-H_2, (**d**) 2.0Pt/m-Al_2O_3-O_2, (**e**) 0.5Pt/m-Al_2O_3-O_2, (**f**) 0.2Pt/m-Al_2O_3-H_2, (**g**) 0.2Pt/m-Al_2O_3-O_2, (**h**) H_2PtCl_6, and (**i**) PtO_2. The spectra exhibited a decreasing

trend in the intensities of white-line: PtO_2 (2.20) > H_2PtCl_6 (2.08) > 0.2Pt/m-Al_2O_3-O_2 (1.66) ≈ 0.2Pt/m-Al_2O_3-H_2 (1.65) > 0.5Pt/m-Al_2O_3-O_2 (1.63) > 2.0Pt/m-Al_2O_3-O_2 (1.51) > 0.5Pt/m-Al_2O_3-H_2 (1.37) > 2.0Pt/m-Al_2O_3-H_2 (1.34) > Pt foil (1.25). The Pt valence state is decreasing with the same trend.

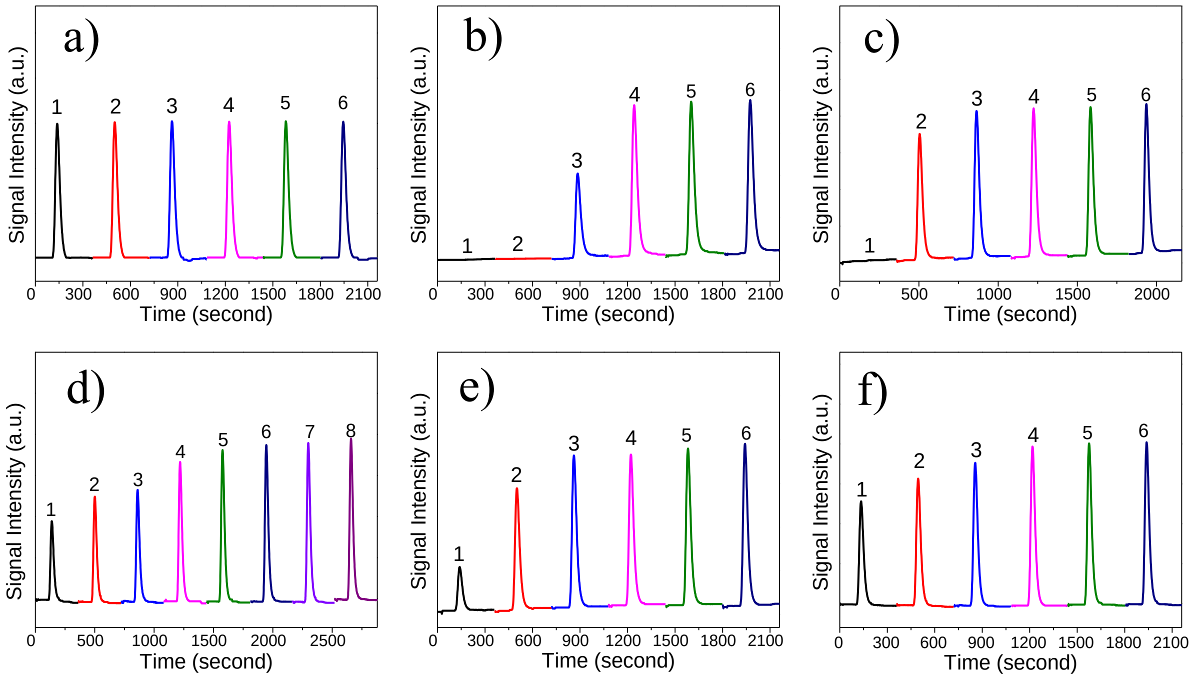

Supplementary Figure 15 | H_2-O_2 titration curves for Pt/m-Al_2O_3. (**a**) 200 mg pure m-Al_2O_3, (**b**) 500 mg 0.2Pt/m-Al_2O_3-O_2, (**c**) 200 mg 0.5Pt/m-Al_2O_3-O_2, and (**d**) 0.5Pt/m-Al_2O_3-H_2, (**e**) 50 mg 2.0Pt/m-Al_2O_3-O_2, and (**f**) 2.0Pt/m-Al_2O_3-H_2. 0 mole H_2 consumption was observed for pure m-Al_2O_3; 2.7 mole H_2 per mol of Pt for 0.2Pt/m-Al_2O_3-O_2; 1.33 mole H_2 per mole of Pt for 0.5Pt/m-Al_2O_3-O_2; 1.25 mole H_2 per mol of Pt for 0.5Pt/m-Al_2O_3-H_2; 0.96 mole H_2 per mol of Pt for 2.0Pt/m-Al_2O_3-O_2; 0.80 mole H_2 per mol of Pt for 2.0Pt/m-Al_2O_3-H_2.

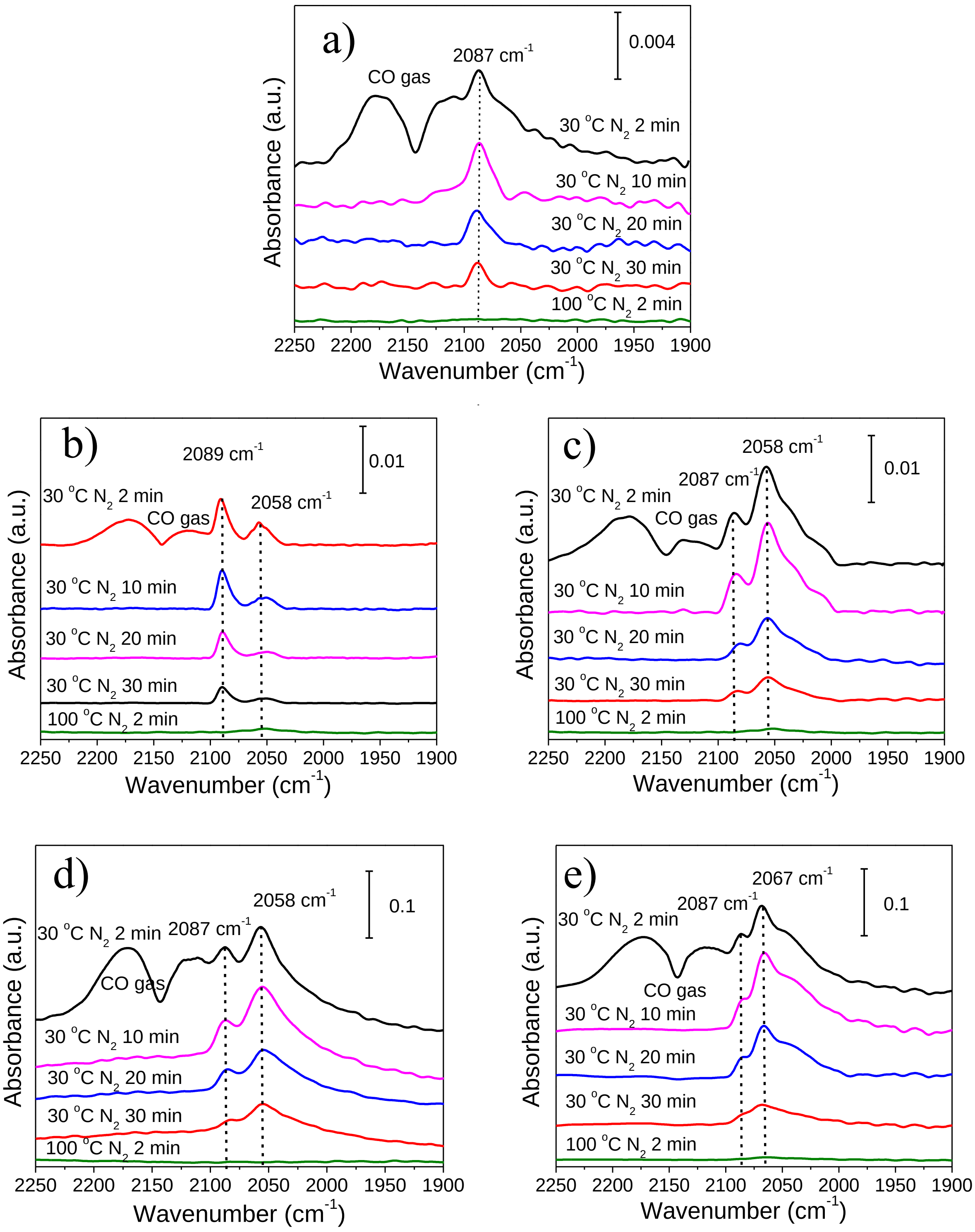

Supplementary Figure 16 | IR spectra of CO adsorbed on Pt/m-Al_2O_3. (**a**) 0.2Pt/m-Al_2O_3-O_2, (**b**) 0.5Pt/m-Al_2O_3-O_2, (**c**) 0.5Pt/m-Al_2O_3-H_2, (**d**) 2.0Pt/m-Al_2O_3-O_2, and (**e**) 2.0Pt/m-Al_2O_3-H_2. The bands at 2058 and 2067 cm^{-1} are ascribed to the linearly bonded CO on Pt^0 sites, while the band at 2087-2089 cm^{-1} is ascribed to CO linearly adsorbed on $Pt^{\delta+}$.

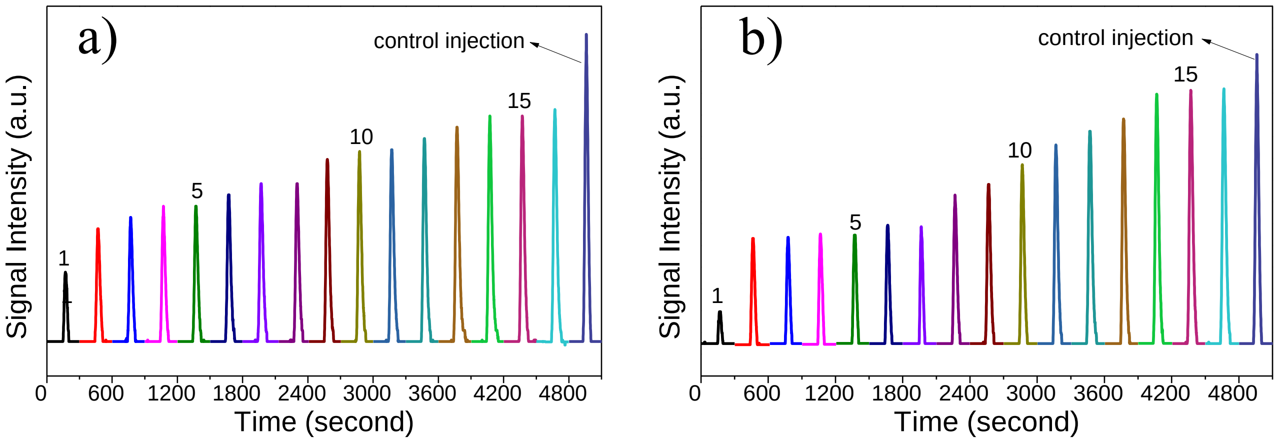

Supplementary Figure 17 | CO titration curves for 0.2Pt/m-Al_2O_3 samples. (**a**) 0.2Pt/m-Al_2O_3-O_2 and (**b**) 0.2Pt/m-Al_2O_3-H_2. 250 mg sample was used in each titration experiment. The control injection (the last peak) indicates the peak height for full CO dosage (no CO adsorption). CO adsorption did not get saturated even if a large excess of CO gas has been provided in consecutive 16 runs, which highlights that the adsorption of CO on Pt single atoms is not very strong, and is constantly under adsorption-desorption equilibrium condition.

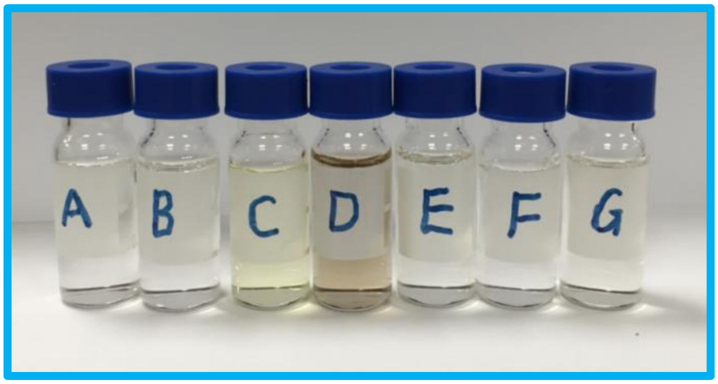

Supplementary Figure 18 | Photograph of solutions containing, Al, Pt and Al-Pt precursors. (**A**) $C_9H_{21}AlO_3$ (5.0 μmol), (**B**) P123 (50 mg), (**C**) H_2PtCl_6 (5.0 μmol) (light yellow in color), (**D**) H_2PtCl_6 (5.0 μmol) + P123 (50 mg) (light beige in color); (**E**) H_2PtCl_6 (5.0 μmol) + $C_9H_{21}AlO_3$ (5.0 μmol); (**F**) $C_9H_{21}AlO_3$ (5.0 μmol) + P123 (50 mg), and (**G**) H_2PtCl_6 (5.0 μmol) + $C_9H_{21}AlO_3$ (5.0 μmol) + P123 (50 mg) in HNO_3 (67%, 0.4 mL) and ethanol (5 mL). In an ethanol solution containing $(EO)_{20}(PO)_{70}(EO)_{20}$ triblock copolymer (P123) template, the $C_9H_{21}AlO_3$ molecules coordinate with Pt^{2+} ions, which were formed by reducing Pt^{4+} ions in ethanol (see Supplementary Fig. 18), forming an almost colorless complexes (H_2PtCl_6 + $C_9H_{21}AlO_3$, and H_2PtCl_6 + $C_9H_{21}AlO_3$ + P123), while H_2PtCl_6 + P123 show light beige in color.

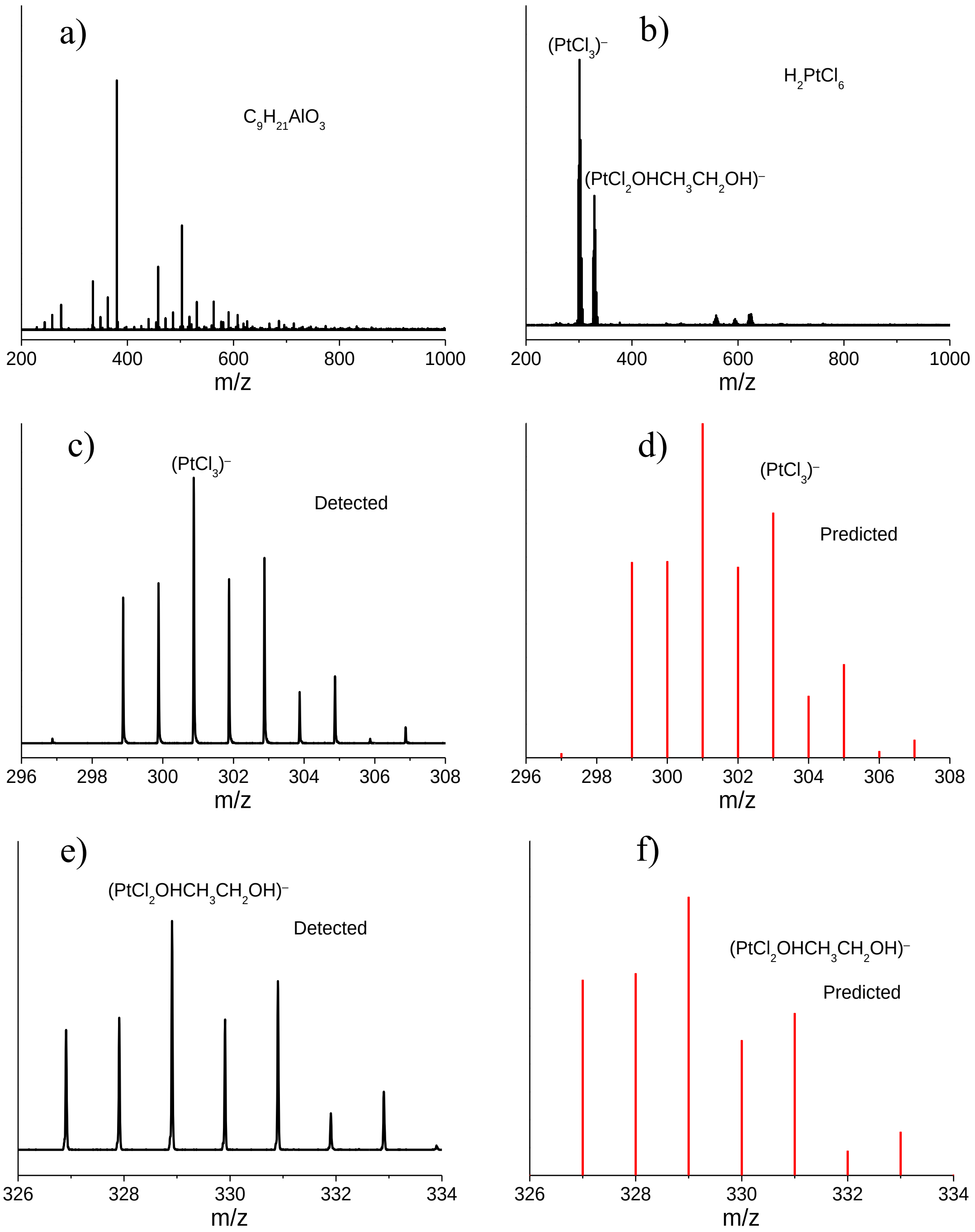

Supplementary Figure 19 | ESI mass spectra (negative mode) of Al precursor and Pt precursor. (**a**) aluminum isopropoxide ($C_9H_{21}AlO_3$) (5.0 μmol), (**b,c,e**) H_2PtCl_6 (5.0 μmol) in HNO_3 (67%, 0.4 mL) and ethanol (5 mL), (**d**) predicted isotope distribution of $PtCl_3^-$, and (**f**) predicted isotope distribution of $PtCl_2OHEtOH^-$. From the ESI-MS spectra of Supplementary Fig. 19, the Pt^{4+} was reduced to Pt^{2+} after stirring in ethanol solution for 48 h, acting as the real Pt precursor.

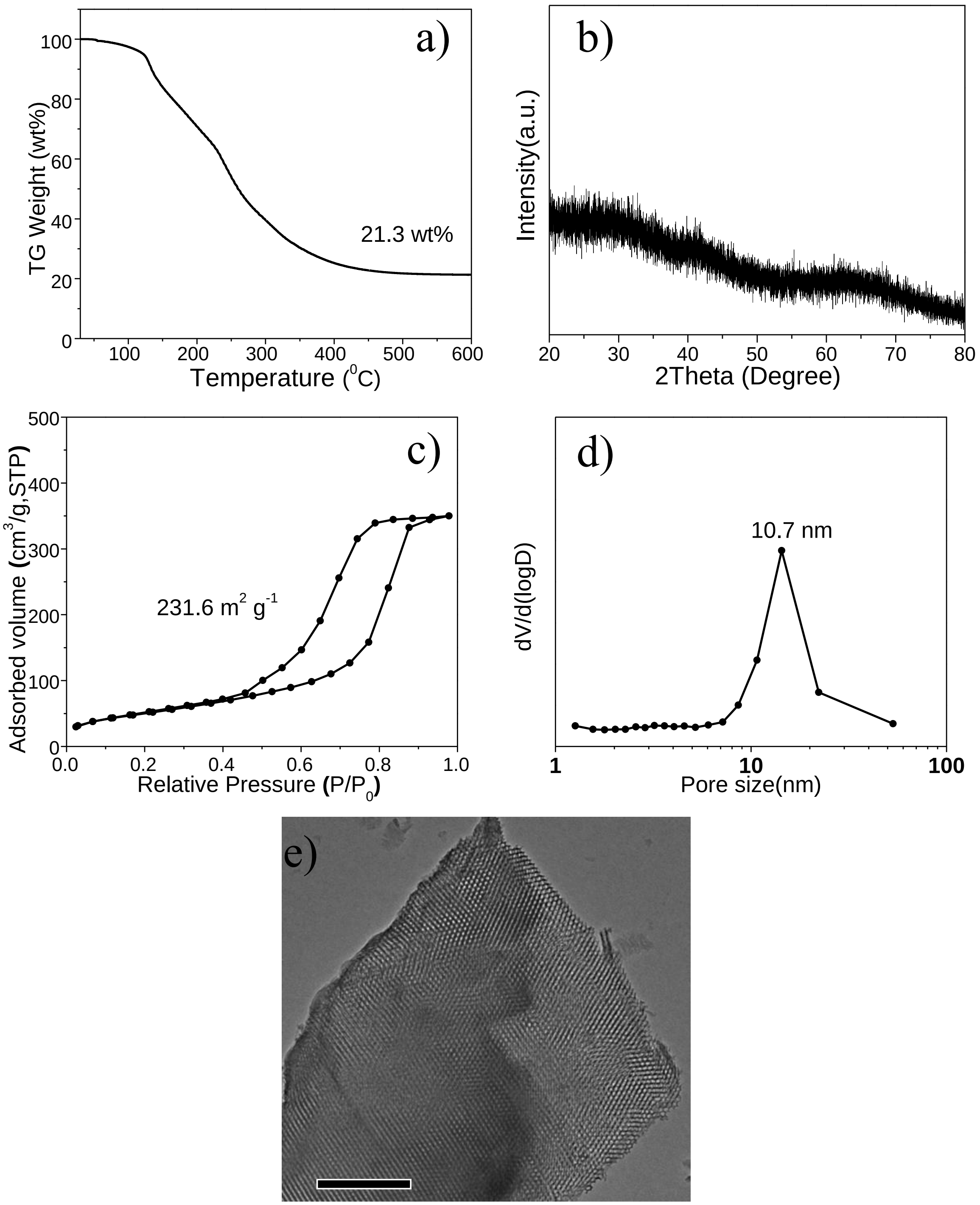

Supplementary Figure 20 | Characterizations of m-Al_2O_3 and the precursor. (a) TG curve of aluminum isopropoxide and Pluronic P123 mixture in air from 30°C-600 °C, (b) XRD pattern of the mixture, (c, d) N_2 adsorption-desorption isotherm and the PSD curve, and (e) TEM image of pure m-Al_2O_3 after calcination at 400 °C in air. Scale bar, 200 nm **(e)**. Supplementary Fig. 20 indicates that high surface area, amorphous and mesoporous Al_2O_3 was obtained using the sol-gel method.

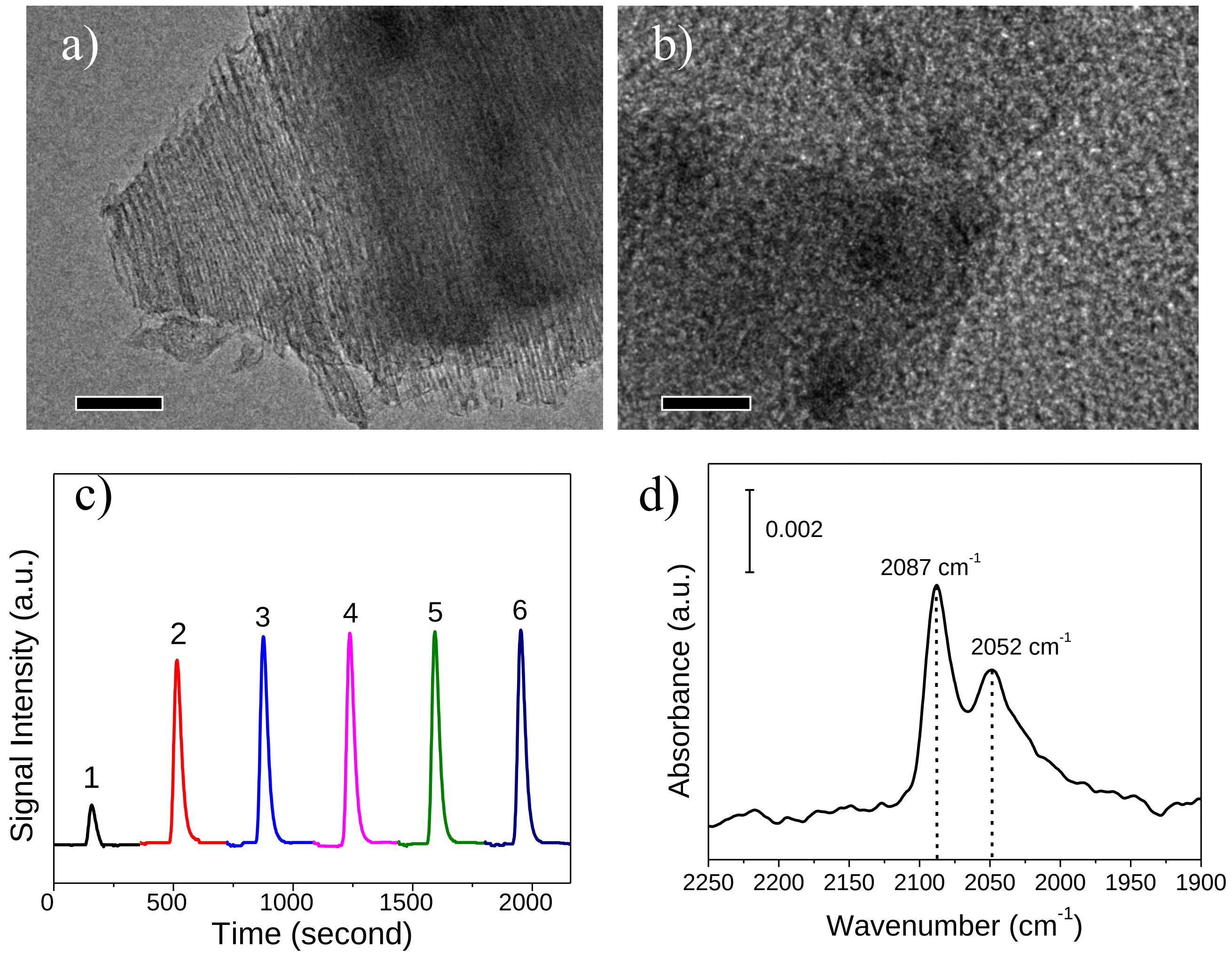

Supplementary Figure 21 | Characterizations of 0.2 wt% Pt on m-Al_2O_3 (0.2Pt/m-Al_2O_3-imp) using a conventional wet-impregnation method. (**a,b**) TEM images, (**c**) H_2-O_2 titration curves (250 mg catalyst), and (**d**) IR spectrum of CO adsorption for sample prepared by calcination in air at 400°C, followed by reduction in 5% H_2/N_2 at 400 °C. Scale bar, 100 nm **(a)**, 5 nm **(b)**. TEM images (Supplementary Fig. 21a,b) showed the formation of small Pt nanoclusters after reduction in 5% H_2/N_2 at 400°C. In the titration experiment, one mole Pt consumed 1.13 mole H_2 (Supplementary Fig. 21c), much lower than single-atom 0.2Pt/m-Al_2O_3-H_2 catalyst prepared by sol-gel method. Two bands at 2052 cm^{-1} and 2087 cm^{-1} are observed, further confirming the formation of Pt nanoclusters prepared via a conventional wet-impregnation method (Supplementary Fig. 21d), highlighting the importance of complexing effect between Pt and Al precursors.

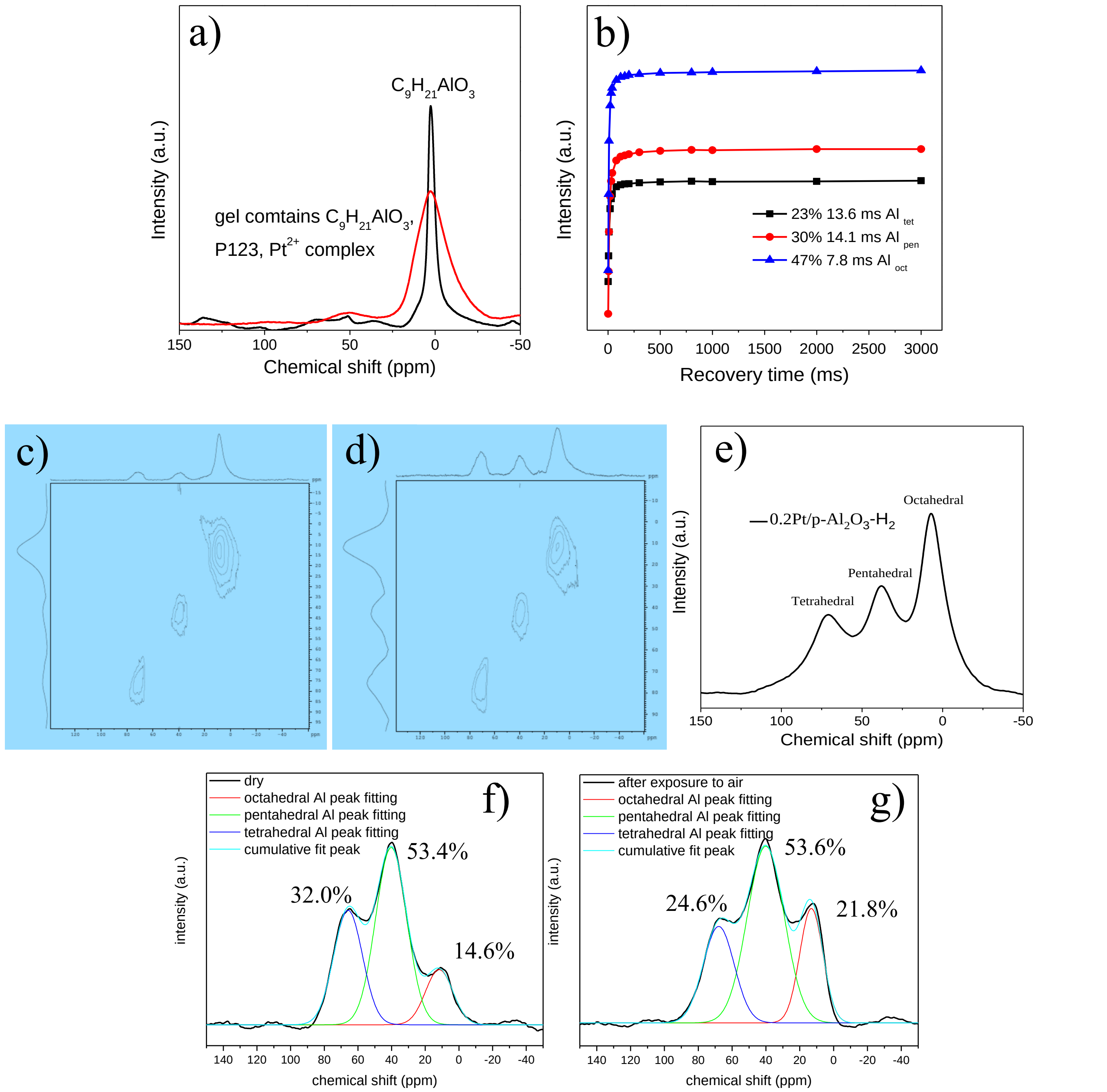

Supplementary Figure 22 | The ^{27}Al MAS-NMR spectra of catalyst and catalyst precursors. (**a**) $C_9H_{21}AlO_3$ and gel contains $C_9H_{21}AlO_3$, P123 and Pt^{2+} complex, (**b**) relative intensity changes of tetra-, penta-, and octa-coordinated Al_2O_3 with recovery time for a spin-lattice relaxation (T1) measurement of Al_2O_3 for 0.2Pt/m-Al_2O_3-O_2, two-dimensional ^{27}A MQ NMR spectrum of (**c**) 0.2Pt/m-Al_2O_3-O_2 and (**d**) 0.2Pt/m-Al_2O_3-H_2, and (**e**) ^{27}Al MAS-NMR spectra of 0.2Pt/p-Al_2O_3-H_2,[2] ^{27}Al MAS-NMR spectra and peak deconvolutions of 0.2Pt/m-Al_2O_3-H_2 (**f**) before and (**g**) after exposure to air. According to the T1 distributions and two-dimensional ^{27}Al MQ NMR spectrum measurement, the percentage of pentahedraly coordinated Al^{3+} centers in 0.2Pt/m-Al_2O_3-O_2 is about 30%. For the peak fittings in f) and

g), constrains including peak position and/or peak width were considered in initial attempts. However, no acceptable fitting results were achieved. This suggested that the signals for different Al species in samples before and after exposure to air might not share the same features. As such the final fitting were conducted with no constrains. The surface Al^{IV} and Al^{V} species can interact with adsorbed water molecules to be transformed to Al^{VI} species via partial hydrolysis. The fact that the percentage of Al^{V} species remain unchanged suggests a dominant of Al^{V} species are in the bulk.

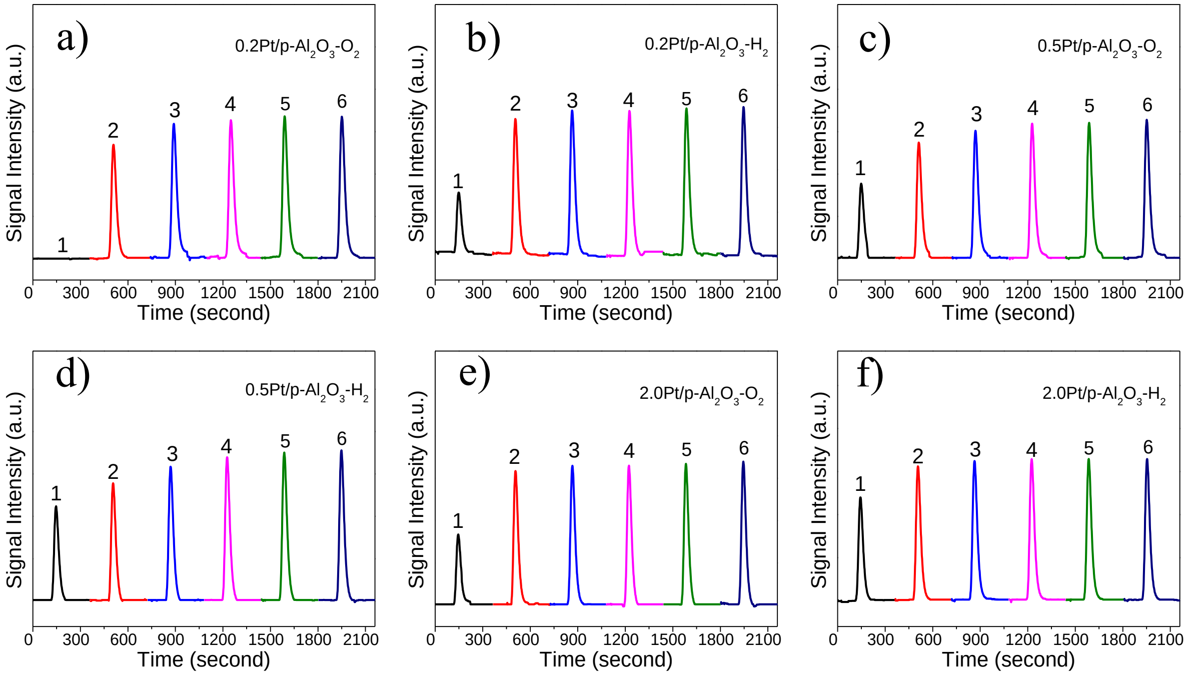

Supplementary Figure 23 | H_2-O_2 titration curves of Pt/p-Al_2O_3. (**a**) 250 mg 0.2Pt/p-Al_2O_3-O_2, (**b**) 250 mg 0.2Pt/p-Al_2O_3-H_2, (**c**) 100 mg 0.5Pt/p-Al_2O_3-O_2, (**d**) 100 mg 0.5Pt/p-Al_2O_3-H_2, (**e**) 25 mg 2.0Pt/p-Al_2O_3-O_2, and (**f**) 25 mg 2.0Pt/p-Al_2O_3-H_2. Consumption of H_2 per mol of Pt: 2.5 mole H_2 for 0.2Pt/p-Al_2O_3-O_2, 1.23 mole H_2 for 0.2Pt/p-Al_2O_3-H_2, 1.2 mole H_2 for 0.5Pt/p-Al_2O_3-O_2, 0.75 mole H_2 for 0.5Pt/p-Al_2O_3-H_2, 0.97 mole H_2 for 2.0Pt/p-Al_2O_3-O_2 and 0.68 mole H_2 for 2.0Pt/p-Al_2O_3-H_2.

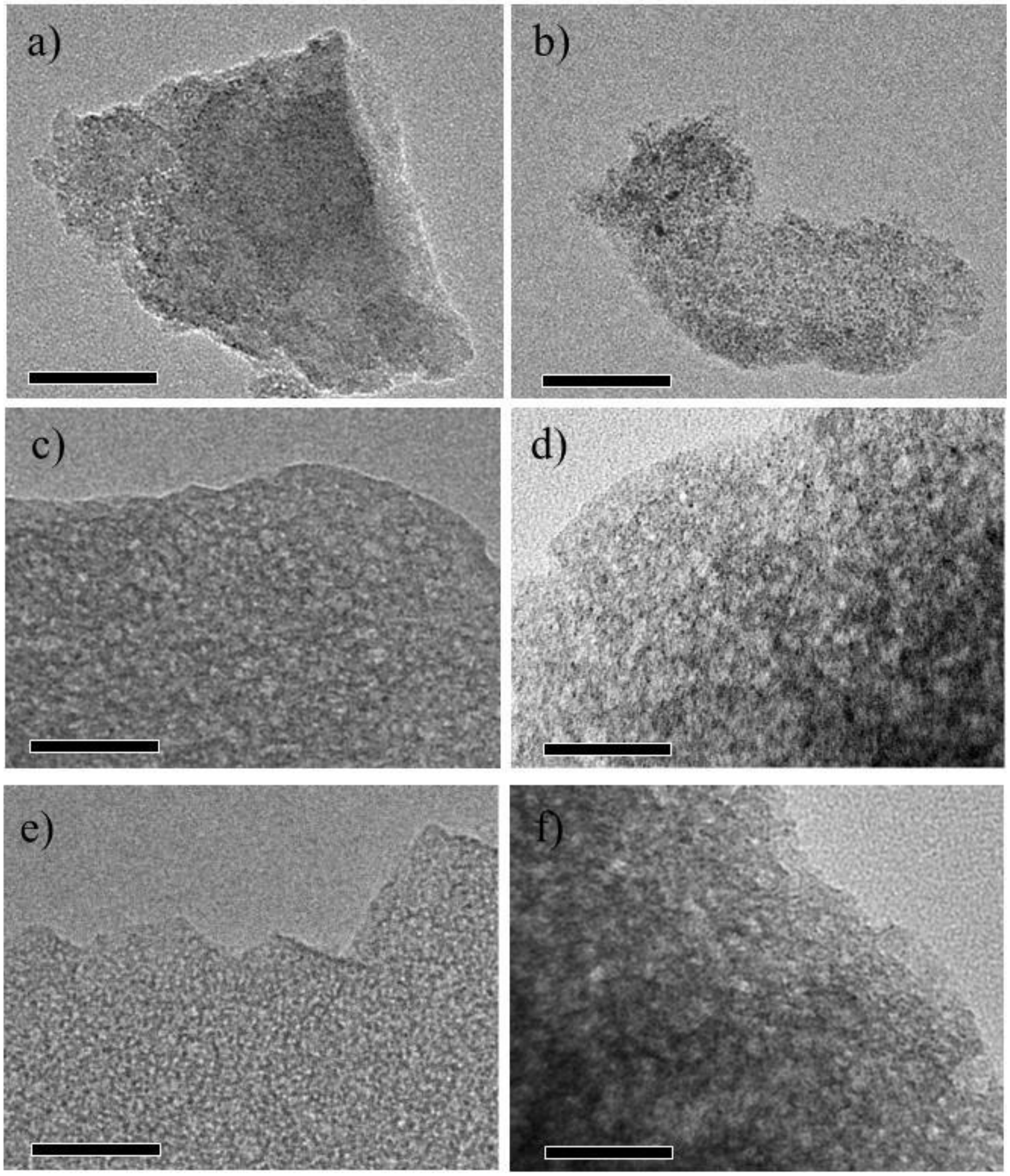

Supplementary Figure 24 | TEM images of some Pt/p-Al_2O_3 samples. (a) 2.0Pt/p-Al_2O_3-O_2, **(b)** 2.0Pt/p-Al_2O_3-H_2, **(c)** 0.5Pt/p-Al_2O_3-O_2, **(d)** 0.5Pt/p-Al_2O_3-H_2, **(e)** 0.2Pt/p-Al_2O_3-O_2, **(f)** 0.2Pt/p-Al_2O_3-H_2 after **(a,c,e)** calcination in air and **(b,d,f)** reduction in 5% H_2/N_2 at 400 °C for 4 h. Scale bar, 50 nm. TEM images showed the formation of Pt nanoclusters and nanoparticles in 2.0Pt/p-Al_2O_3-O_2, 2.0Pt/p-Al_2O_3-H_2, 0.5Pt/p-Al_2O_3-O_2, and 0.5Pt/p-Al_2O_3-H_2 samples. However, for sample 0.2Pt/p-Al_2O_3-O_2, no obvious nanoclusters and nanoparticles were observed. The nanoclusters are formed after H_2 reduction for sample 0.2Pt/p-Al_2O_3-H_2, indicating the Pt single-atom can be converted to nanoclusters in the presence of H_2 at 400 °C on disordered p-Al_2O_3.

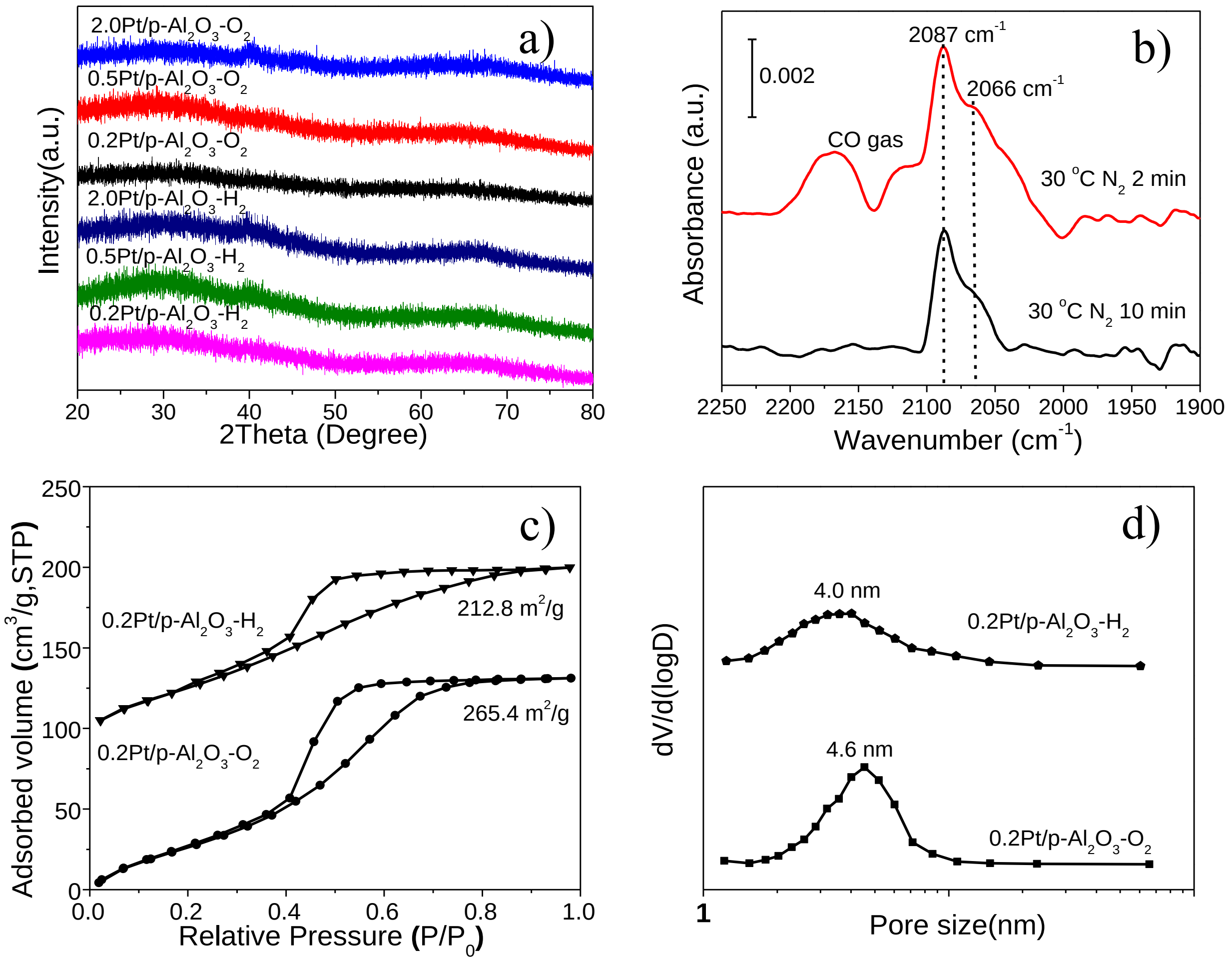

Supplementary Figure 25 | Characterizations of Pt/p-Al_2O_3 catalyst with different Pt loading. (**a**) XRD patterns for samples 2.0Pt/p-Al_2O_3-O_2, 2.0Pt/p-Al_2O_3-H_2, 0.5Pt/p-Al_2O_3-O_2, 0.5Pt/p-Al_2O_3-H_2, 0.2Pt/p-Al_2O_3-O_2, and 0.2Pt/p-Al_2O_3-H_2, (**b**) IR spectra of CO adsorbed on 0.2Pt/p-Al_2O_3-H_2, (**c**) N_2 adsorption-desorption isotherms, and (**d**) their PSD curves for 0.2Pt/p-Al_2O_3-O_2, 0.2Pt/p-Al_2O_3-H_2 (samples 0.2Pt/p-Al_2O_3-H_2 was shifted upwards for 100 cm^3 g^{-1}). XRD patterns in Supplementary Fig. 25a exhibit small Pt peaks for samples 2.0Pt/p-Al_2O_3-O_2, 2.0Pt/p-Al_2O_3-H_2, 0.5Pt/p-Al_2O_3-H_2, while no obvious Pt peaks were observed for samples 0.5Pt/Al_2O_3-O_2, 0.2Pt/p-Al_2O_3-O_2, and 0.2Pt/p-Al_2O_3-H_2. For IR spectra of CO adsorption on 0.2Pt/p-Al_2O_3-H_2 (Supplementary Fig. 25b), two bands at 2087 and 2066 cm^{-1} were observed, confirming that the sample is a mixture of Pt single-atoms and nanoclusters after reduction. The N_2 adsorption-desorption isotherms (Supplementary Fig. 25c,d) showed that the surface area for 0.2Pt/p-Al_2O_3-O_2 and 0.2Pt/p-Al_2O_3-H_2 is 265.4 and 212.8 m^2 g^{-1}, indicating these disorder p-Al_2O_3 is not stable at high-temperature H_2 atmosphere.

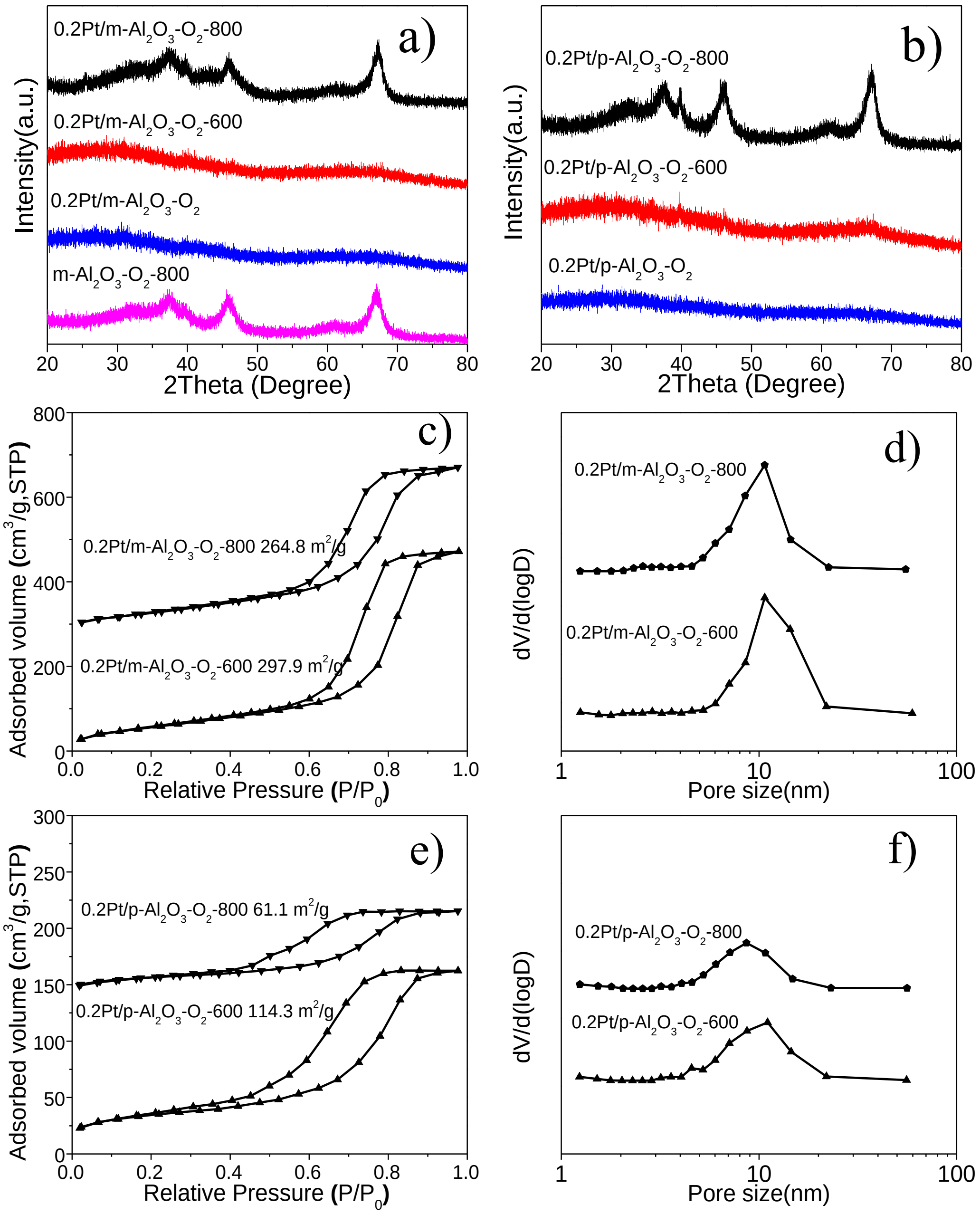

Supplementary Figure 26 | XRD and BET analysis of 0.2Pt/m-Al_2O_3-O_2 and 0.2Pt/p-Al_2O_3-O_2 after high temperature treatment. XRD patterns for (**a**) 0.2Pt/m-Al_2O_3-O_2, 0.2Pt/m-Al_2O_3-O_2-600, 0.2Pt/m-Al_2O_3-O_2-800, m-Al_2O_3-O_2-800, and (**b**) 0.2Pt/p-Al_2O_3-O_2, 0.2Pt/p-Al_2O_3-O_2-600, 0.2Pt/p-Al_2O_3-O_2-800; N_2 adsorption-desorption isotherms and the PSD curves of (**c,d**) 0.2Pt/m-Al_2O_3-O_2-600 and 0.2Pt/m-Al_2O_3-O_2-800, and (**e,f**) 0.2Pt/p-Al_2O_3-O_2-600 and 0.2Pt/p-Al_2O_3-O_2-800. The samples were either treated at 600 °C or 800 °C in air for 4 h. XRD patterns indicate that the amorphous Al_2O_3 become γ-Al_2O_3 after calcination at 800 °C. BET analysis indicates that m-Al_2O_3 with 0.2 wt% embedded Pt can maintain its pore structure and surface area at high-temperature (600 °C and 800 °C). However, the porous structure was destroyed in disordered porous Al_2O_3 reflected by decreased surface area.

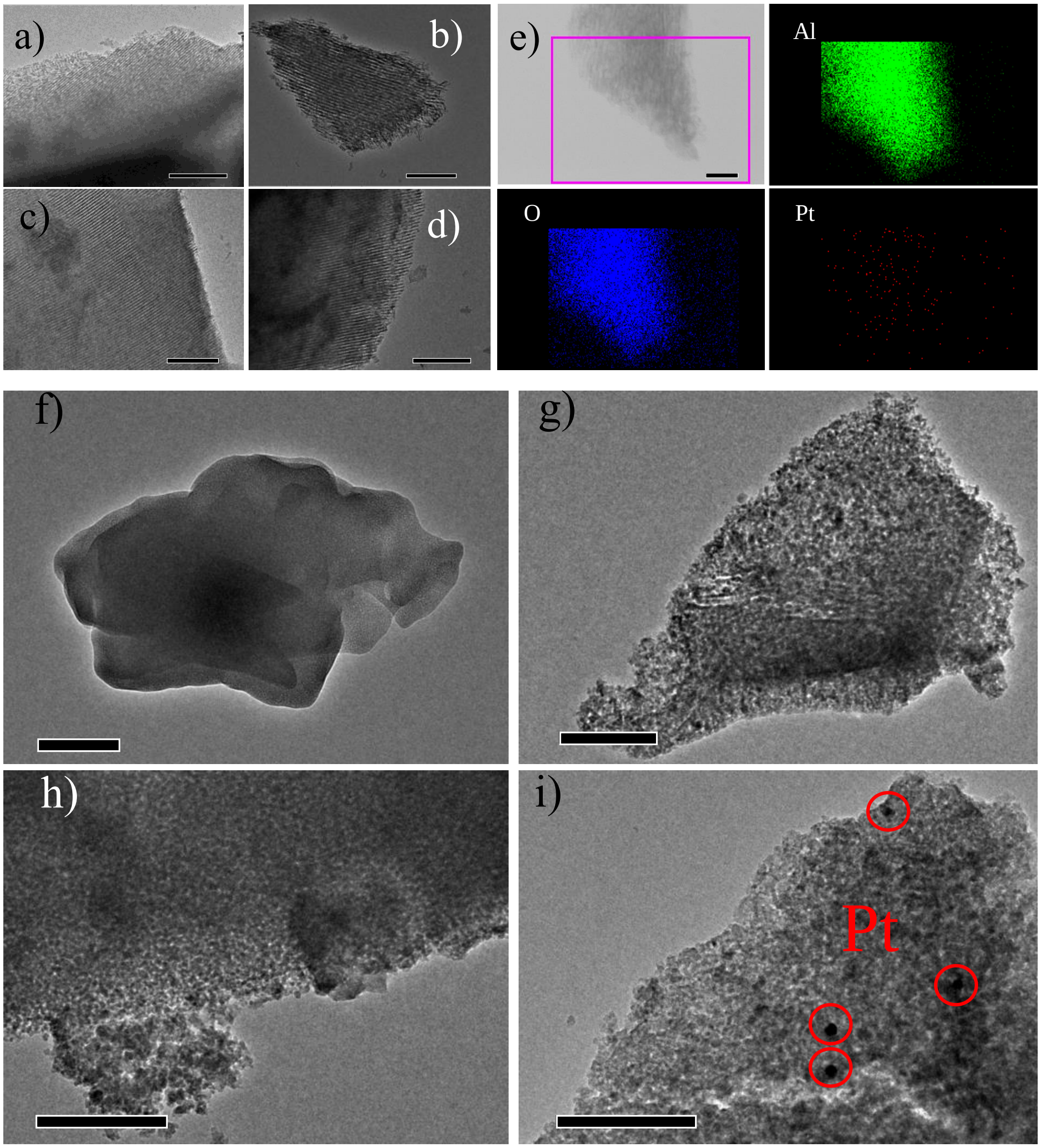

Supplementary Figure 27 | TEM Characterizations of 0.2Pt/m-Al_2O_3-O_2 and 0.2Pt/p-Al_2O_3-O_2 after high temperature treatment. (**a,b**) 0.2Pt/m-Al_2O_3-O_2-600, (**c,d**) 0.2Pt/m-Al_2O_3-O_2-800, (**e**) TEM mapping images of 0.2Pt/m-Al_2O_3-O_2-800, (**f**) 0.2Pt/p-Al_2O_3-O_2, (**g,h**) 0.2Pt/p-Al_2O_3-O_2-600, and (**i**) 0.2Pt/p-Al_2O_3-O_2-800. Scale bar, 200 nm (**a,b,c,d,f,g,h,i**), 100 nm (**e**). TEM analysis for 0.2Pt/m-Al_2O_3-O_2-600 and 0.2Pt/m-Al_2O_3-O_2-800 indicate that the mesoporous structure was well conserved with Pt remaining uniformly dispersed after high-temperature calcination. On the other hand, TEM images clearly indicated the damage of the pore on 0.2Pt/p-Al_2O_3-O_2-600 and 0.2Pt/p-Al_2O_3-O_2-800. Pt nanoparticles were also identified in these samples.

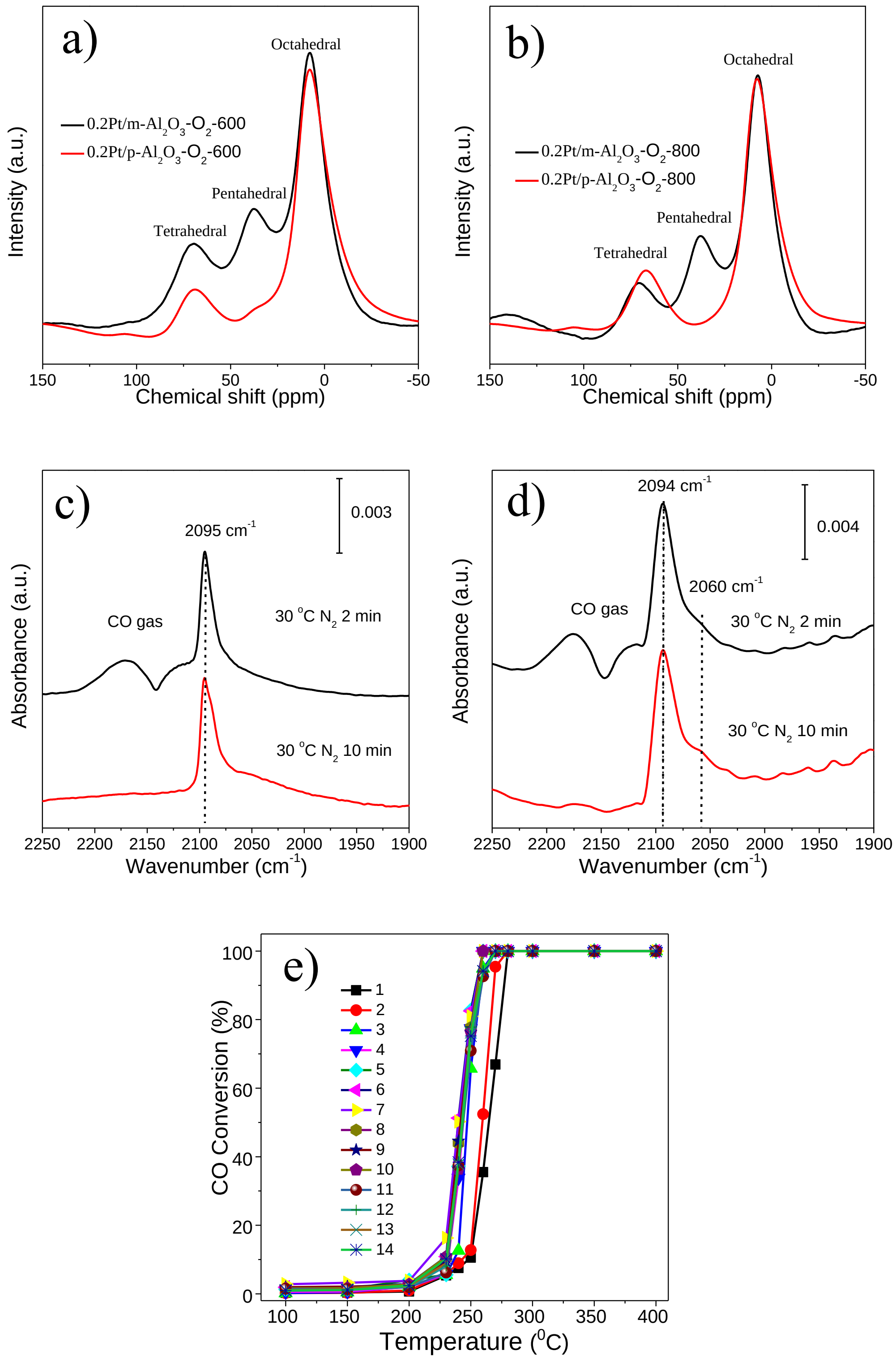

Supplementary Figure 28 | Characterizations of 0.2Pt/m-Al_2O_3-O_2 and 0.2Pt/p-Al_2O_3-O_2 after high temperature treatment. The ^{27}Al MAS-NMR spectra of (**a**) 0.2Pt/m-Al_2O_3-O_2-600 and 0.2Pt/p-Al_2O_3-O_2-600, (**b**) 0.2Pt/m-Al_2O_3-O_2-800 and 0.2Pt/p-Al_2O_3-O_2-800. IR spectra of CO adsorbed on (**c**) 0.2Pt/m-Al_2O_3-O_2-600 and (**d**) 0.2Pt/m-Al_2O_3-O_2-800. (**e**) 1st to 14th cycles of CO conversion over 0.2Pt/m-Al_2O_3-O_2-600 between 100-400 °C. The ^{27}Al MAS-NMR spectra indicate that m-Al_2O_3 maintained a

considerable portion of pentahedral unsaturated Al^{3+} centers after high-temperature calcination (600 ^{o}C and 800 ^{o}C), whereas p-Al_2O_3 lost most of penta-coordinated Al^{3+} species. IR spectra of adsorbed CO suggested that a majority of the Pt species remained as isolated $Pt^{\delta+}$ species after calcination at 600 and 800 ^{o}C, with co-existence of nanoparticles.

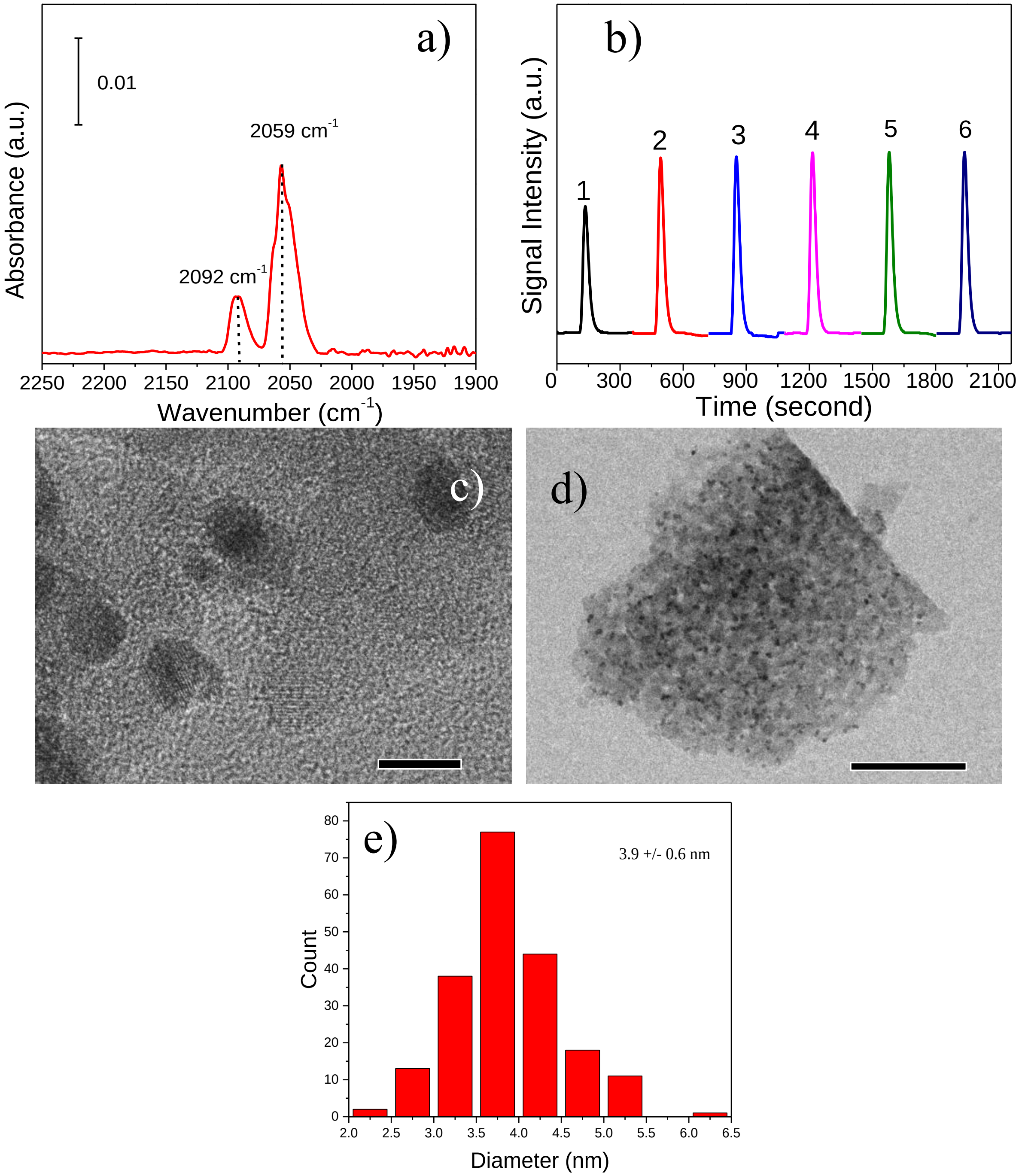

Supplementary Figure 29 | Characterizations for fresh commercial Pt/Al_2O_3 catalyst. (**a**) IR spectra of CO adsorption (diluted 25 times with commercial Al_2O_3), (**b**) H_2-O_2 titration profiles, (**c,d**) TEM images and (**e**) size distribution. Scale bar, 5 nm (**c**), 100 nm (**d**). The bands at about 2059 cm^{-1} and 2092 cm^{-1} are ascribed to the linearly bonded CO on Pt^{0} sites, and $Pt^{\delta+}$ on the surface of Pt nanoparticles,

respectively, indicating that most of Pt species are nanoparticles on commercial Pt/Al_2O_3 catalyst (Supplementary Fig. 29a). H_2-O_2 titration showed that one mole Pt consumed 0.61 mole H_2 (Supplementary Fig. 29b), consistent with the dispersion of small Pt nanoparticles. TEM images (Supplementary Fig. 29c,d) indicate that the size of Pt nanoparticles is centered at around 4 nm in commercial Pt/Al_2O_3 catalyst.

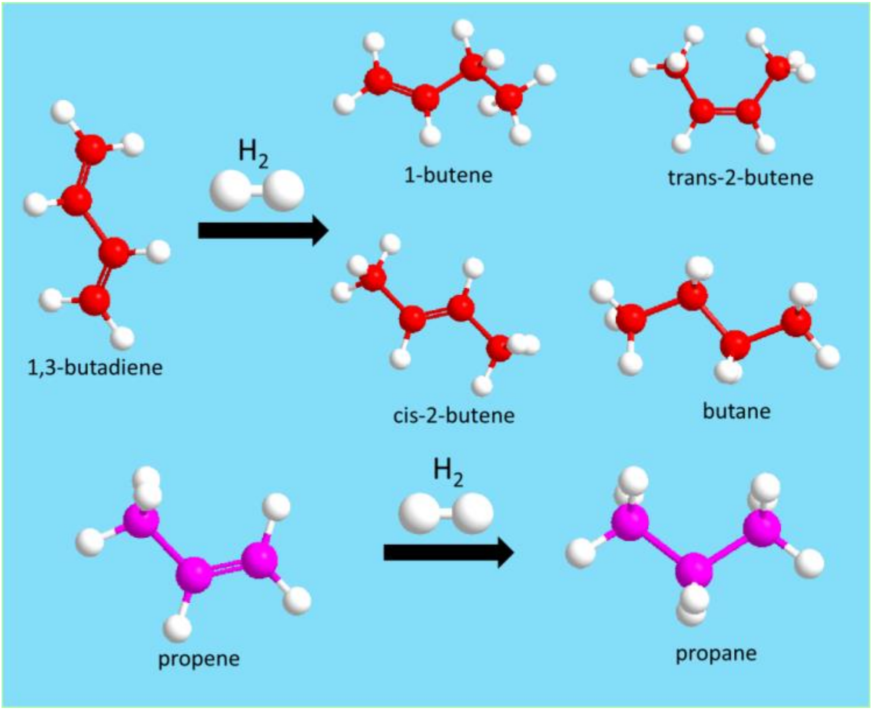

Supplementary Figure 30 | Possible products from selective hydrogenation of 1,3-butadiene in the presence of propylene, including butane, trans-2-butene, 1-butene, cis-2-butene and propane.

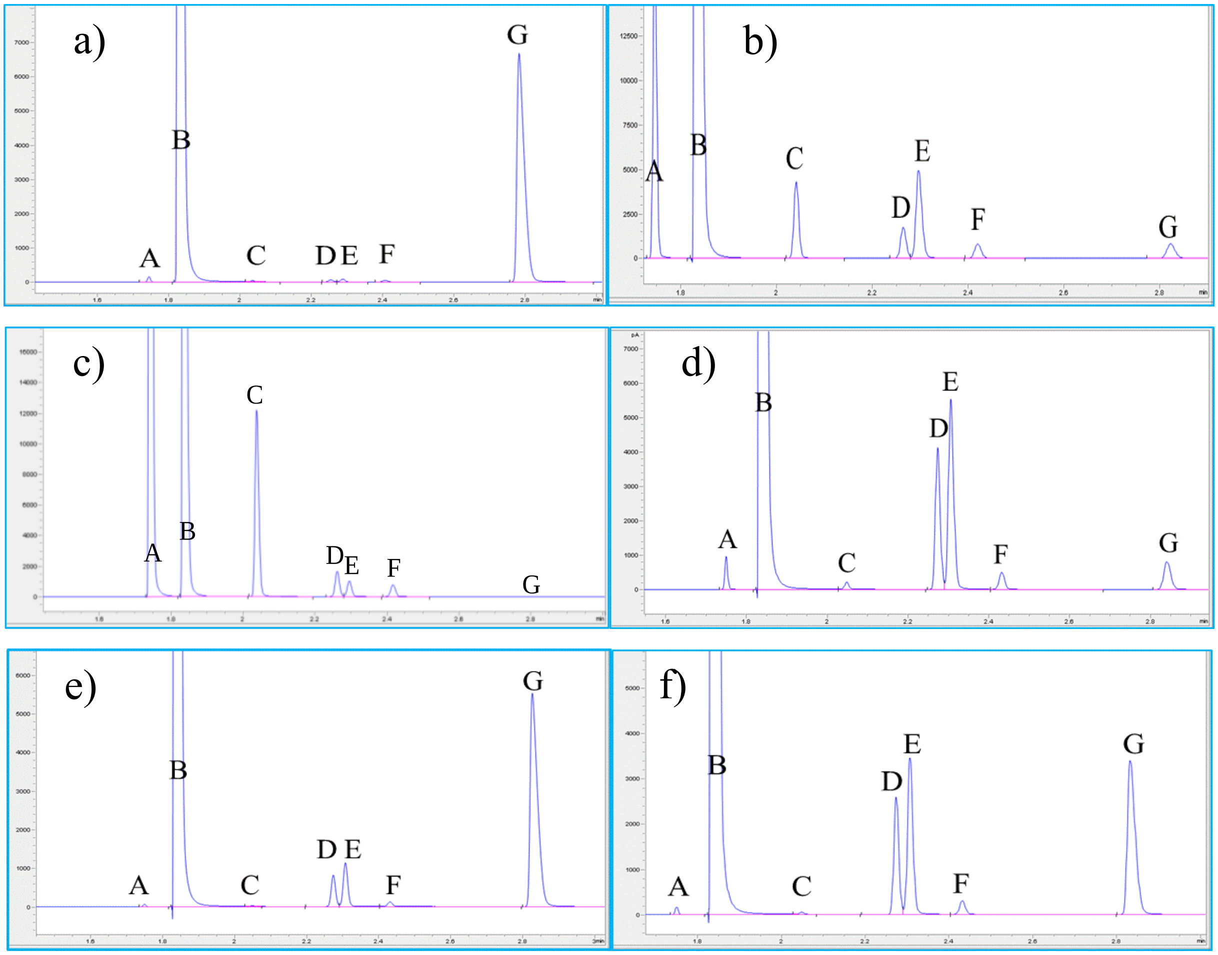

Supplementary Figure 31 | Representative GC data for 1,3-butadiene selective hydrogenation. (**a**) Pure Al_2O_3 at 50 ^{o}C, (**b**) 0.2Pt/p-Al_2O_3-H_2 ^{o}C, (**c**) commercial Pt/Al_2O_3 at 50 ^{o}C, and 0.2Pt/m-Al_2O_3-H_2 at (**d**) 50 ^{o}C, (**e**) 30 ^{o}C, and (**f**) 40 ^{o}C. A, B, C, D, E, F and G stand for propane, propene, butane, trans-2-butene, 1-butene, cis-2-butene, and 1,3-butadiene, respectively.

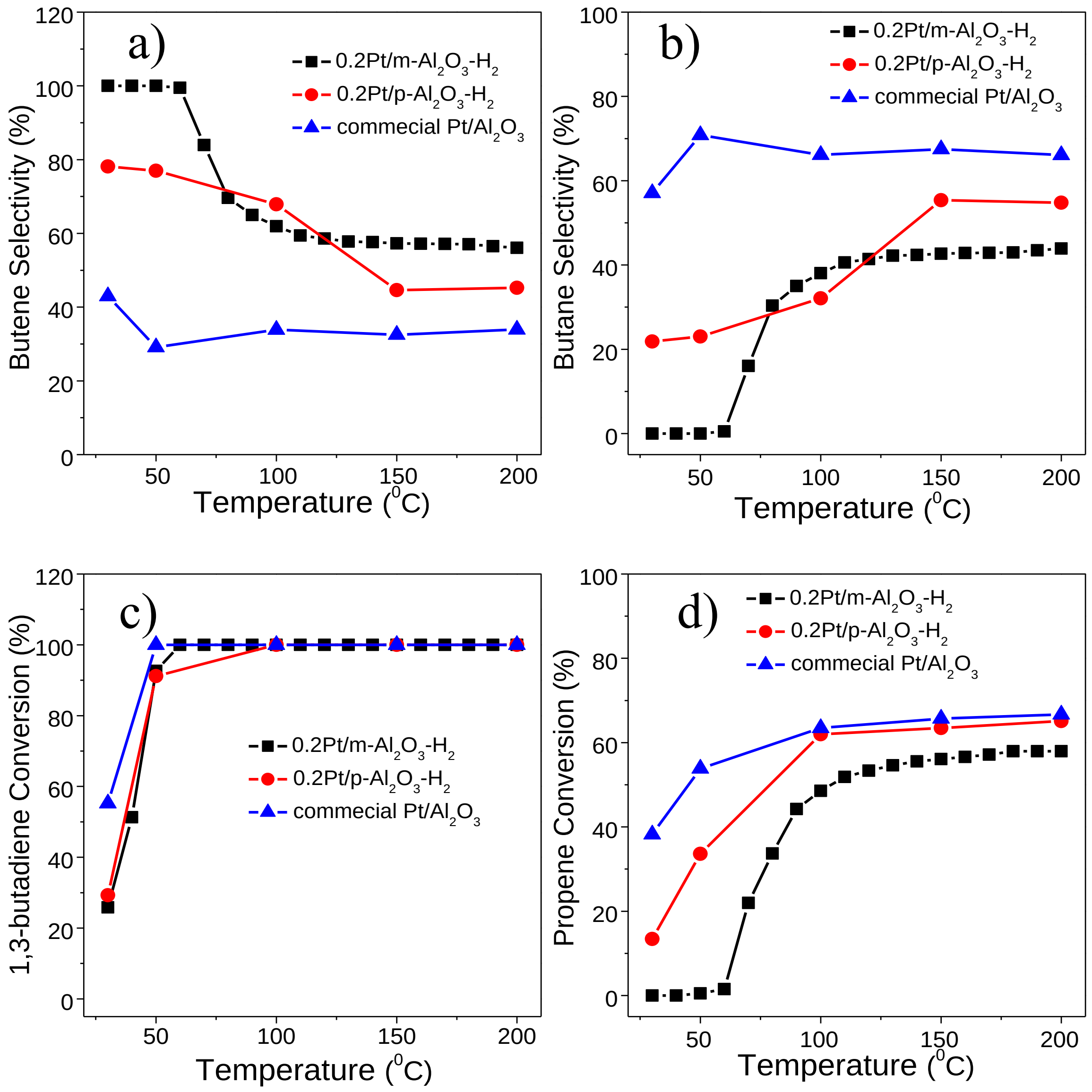

Supplementary Figure 32 | Catalytic performance of various catalysts in selective 1,3-butadiene hydrogenation at different temperature. The selectivity of (**a**) butene, (**b**) butane, the conversion of (**c**) 1,3-butadiene, (**d**) propene for 0.2Pt/m-Al_2O_3-H_2, 0.2Pt/p-Al_2O_3-H_2 and commercial Pt/Al_2O_3 catalysts from 30 ºC-200 ºC. Supplementary Fig. 32 indicates that only single-atom 0.2Pt/p-Al_2O_3-H_2 catalyst can selectively hydrogenate 1,3-butadiene forming mono-ene at low-temperature, whereas butane and propane were produced over control samples (0.2Pt/p-Al_2O_3-H_2 and commercial Pt/Al_2O_3). At higher temperature (> 60 °C), on the other hand, full hydrogenation occurred over all catalysts.

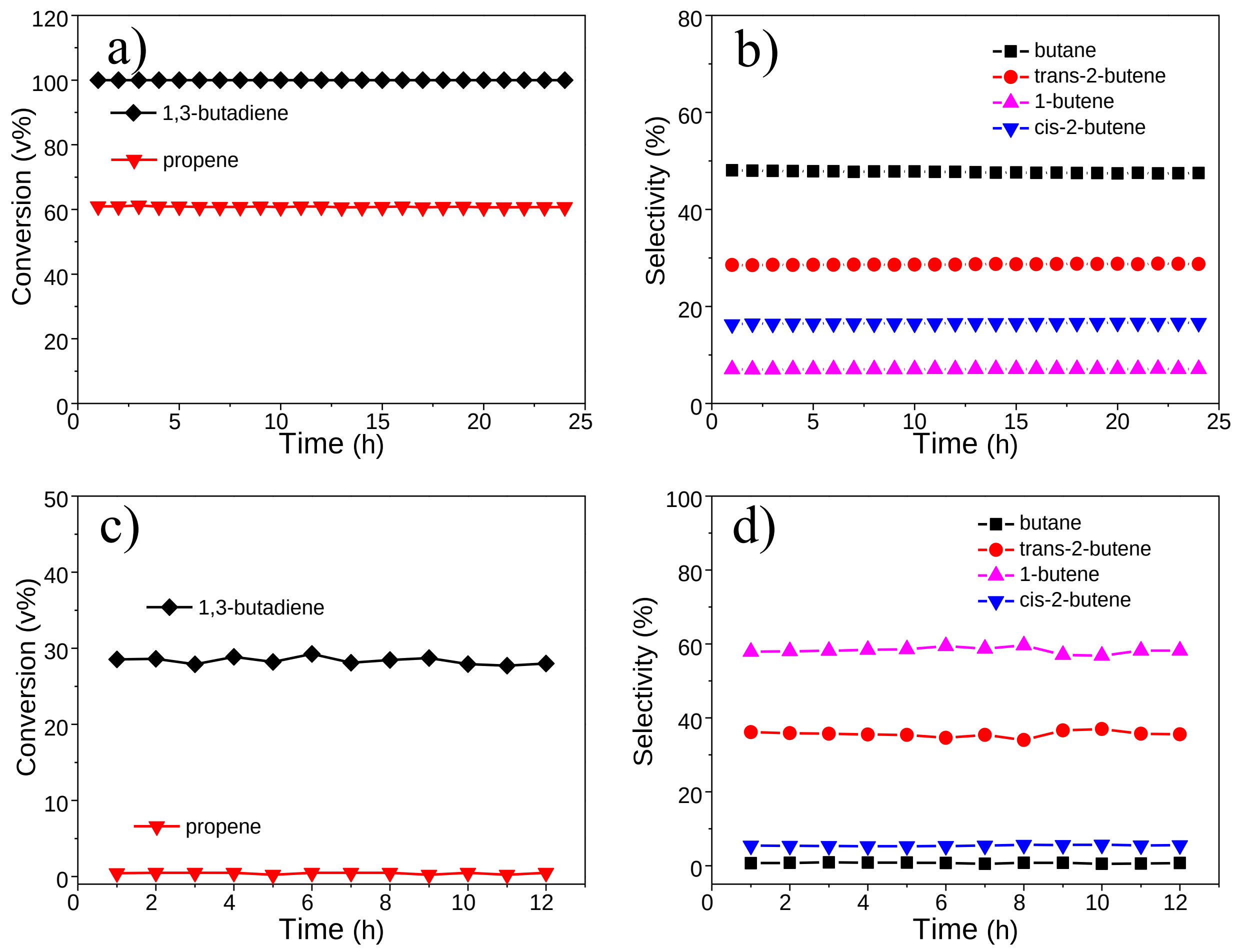

Supplementary Figure 33 | Deactivation test of 0.2Pt/m-Al_2O_3-H_2 catalyst in selective 1,3-butadiene hydrogenation. The catalyst was treated at 200 °C for 24 h and then re-evaluated at 30 °C for 12 h. The catalyst was pretreated in 5% H_2/N_2 atmospheres at 400 °C for 1 h before the reaction. (**a**) The conversion of 1,3-butadiene and propene, (**b**) the selectivity of butane, trans-2-butene, 1-butene, and cis-2-butene at 200 °C for 24 h; (**c**) the conversion of 1,3-butadiene and propene and (**d**) the selectivity of butane, trans-2-butene, 1-butene, and cis-2-butene at 30 °C for 12 h.

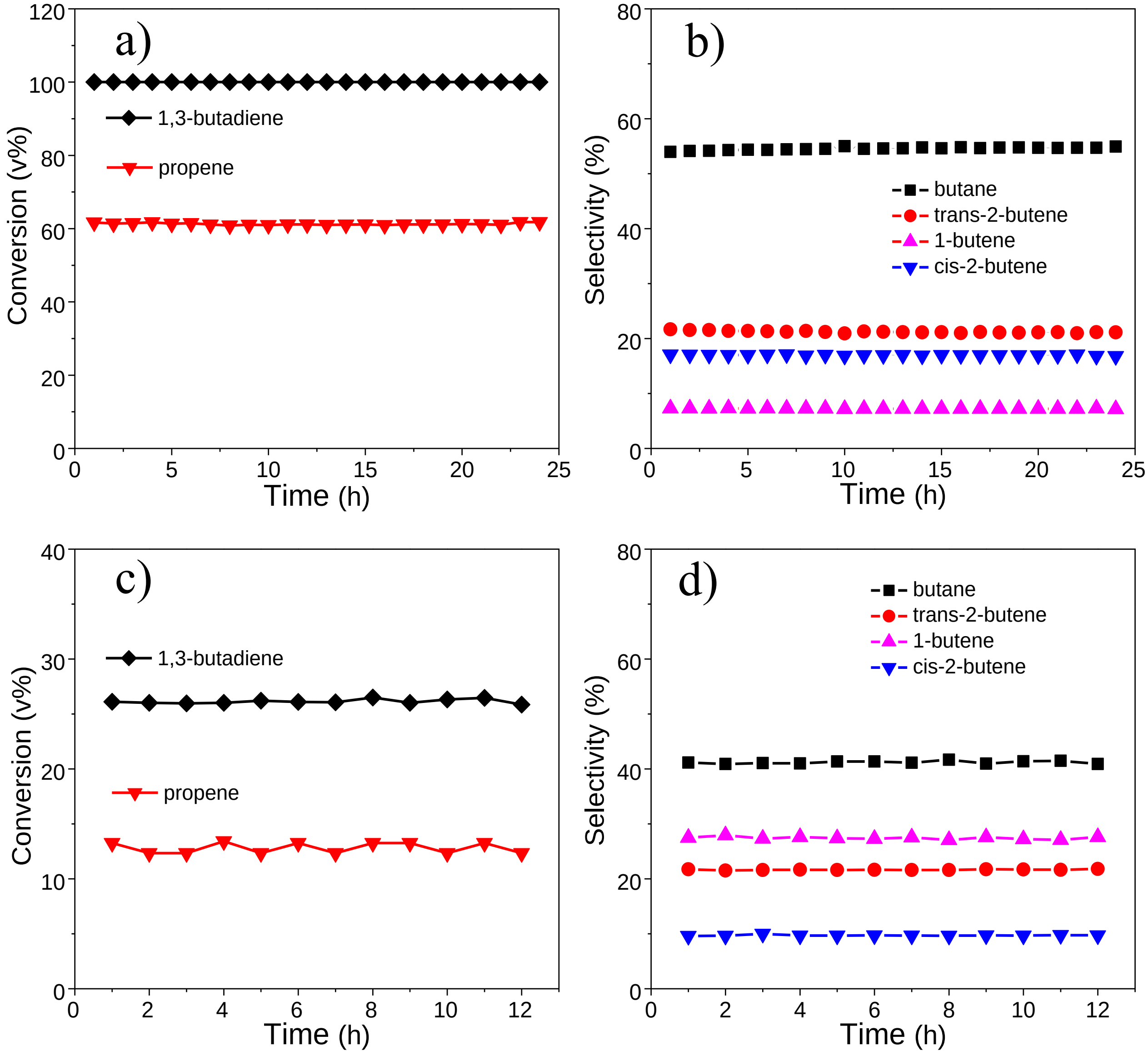

Supplementary Figure 34 | Deactivation test of 0.2Pt/p-Al_2O_3-H_2 catalyst in selective 1,3-butadiene hydrogenation. The catalyst was treated at 200 °C for 24 h and then re-evaluated at 30 °C for 12 h. The catalyst was pretreated in 5% H_2/N_2 atmospheres at 400 °C for 1 h before the reaction. (**a**) The conversion of 1,3-butadiene and propene, (**b**) the selectivity of butane, trans-2-butene, 1-butene, and cis-2-butene at 200 °C for 24 h; (**c**) the conversion of 1,3-butadiene and propene and (**d**) the selectivity of butane, trans-2-butene, 1-butene, and cis-2-butene at 30 °C for 12 h.

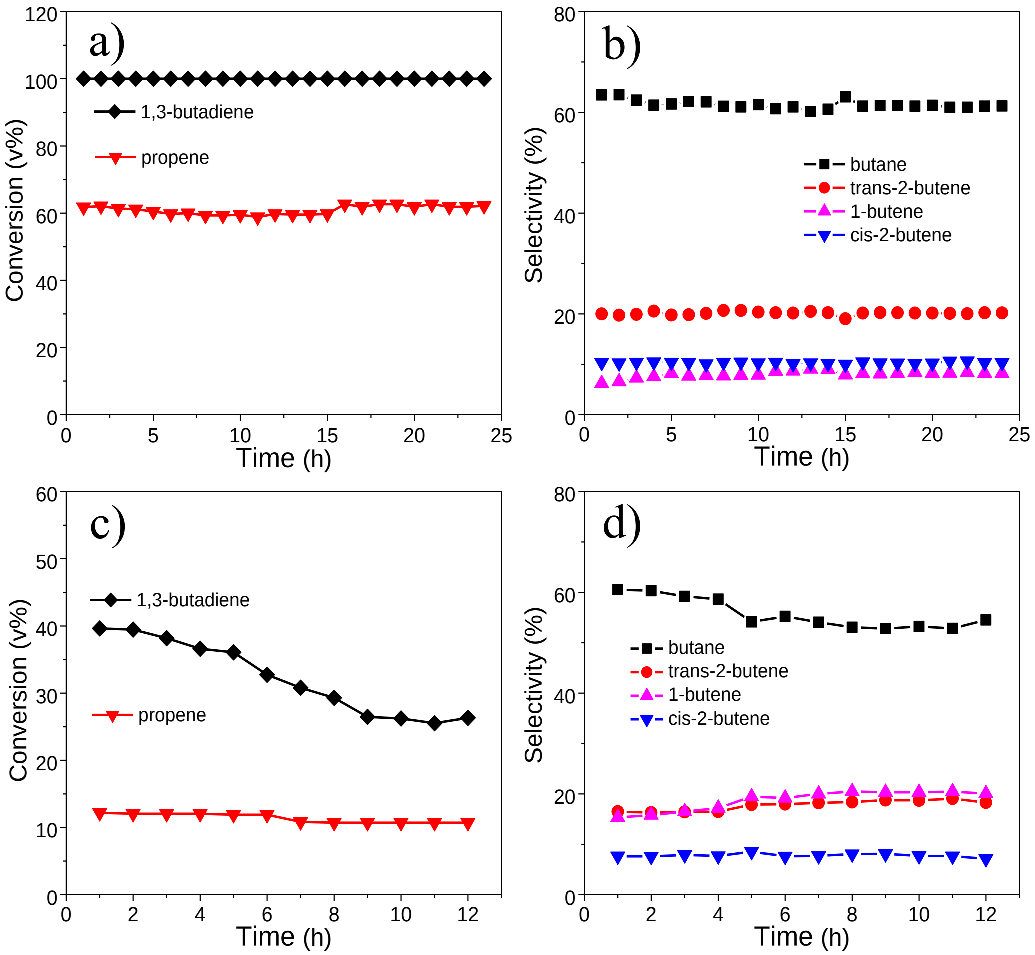

Supplementary Figure 35 | Deactivation test of commercial Pt/Al_2O_3 catalyst in selective 1,3-butadiene hydrogenation. The catalyst was treated at 200 °C for 24 h and then re-evaluated at 30 °C for 12 h. The catalyst was pretreated in 5% H_2/N_2 atmospheres at 400 °C for 1 h before the reaction. (**a**) The conversion of 1,3-butadiene and propene, (**b**) the selectivity of butane, trans-2-butene, 1-butene, and cis-2-butene at 200 °C for 24 h; (**c**) the conversion of 1,3-butadiene and propene and (**d**) the selectivity of butane, trans-2-butene, 1-butene, and cis-2-butene at 30 °C for 12 h.

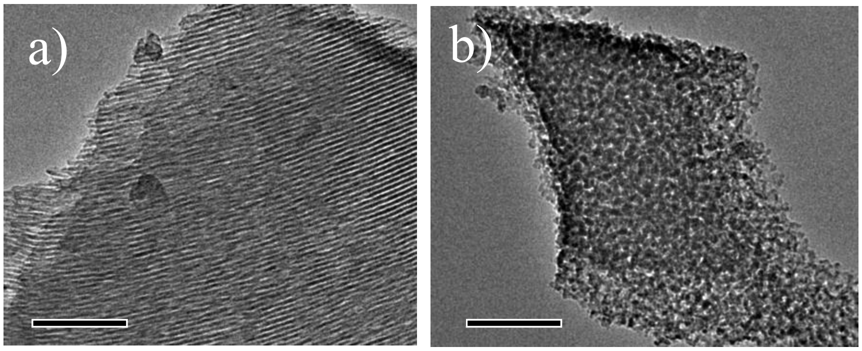

Supplementary Figure 36 | TEM images for spent catalysts after 1,3-butadiene hydrogenation. (**a**) 0.2Pt/m-Al_2O_3-H_2 and (**b**) 0.2Pt/p-Al_2O_3-H_2 at 200 °C for 24 h and then 30 °C for 12 h. Scale bar, 100 nm.

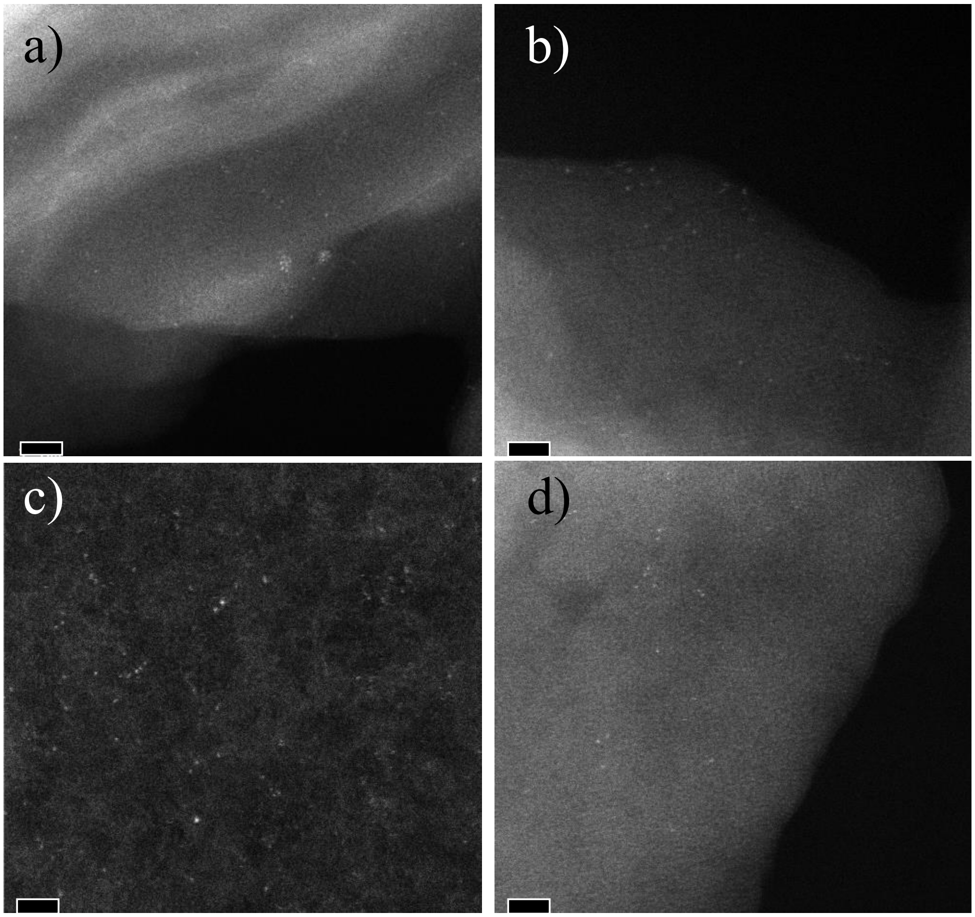

Supplementary Figure 37 | Additional HAADF-STEM images for 0.2Pt/m-Al_2O_3-H_2 after 1,3-butadiene hydrogenation at 200 ºC for 24 h and then at 30 ºC for 12 h. (**a-d**) images taken at different positions of the specimen; scale bar, 2 nm.

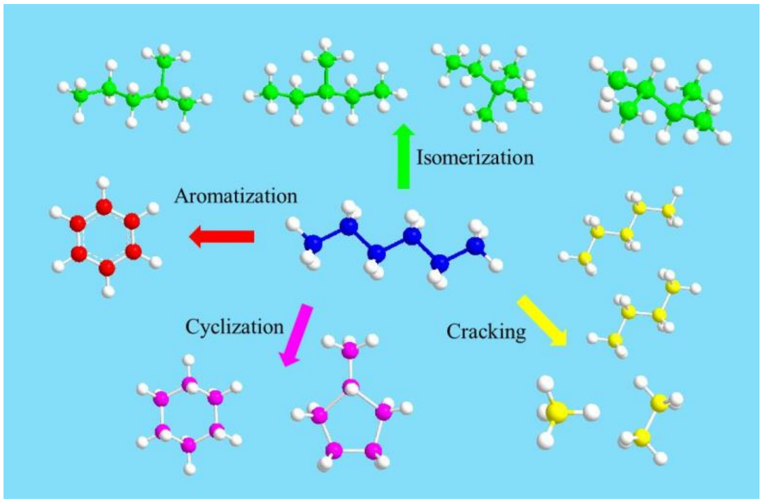

Supplementary Figure 38 | Possible reaction products for *n*-hexane reforming. *n*-hexane can react via four major pathways: cracking to small hydrocarbons; isomerization to 2-methylpentane, 3-methylpentane and others; cyclization to methylcyclopentane or cyclohexane; aromatization to benzene.

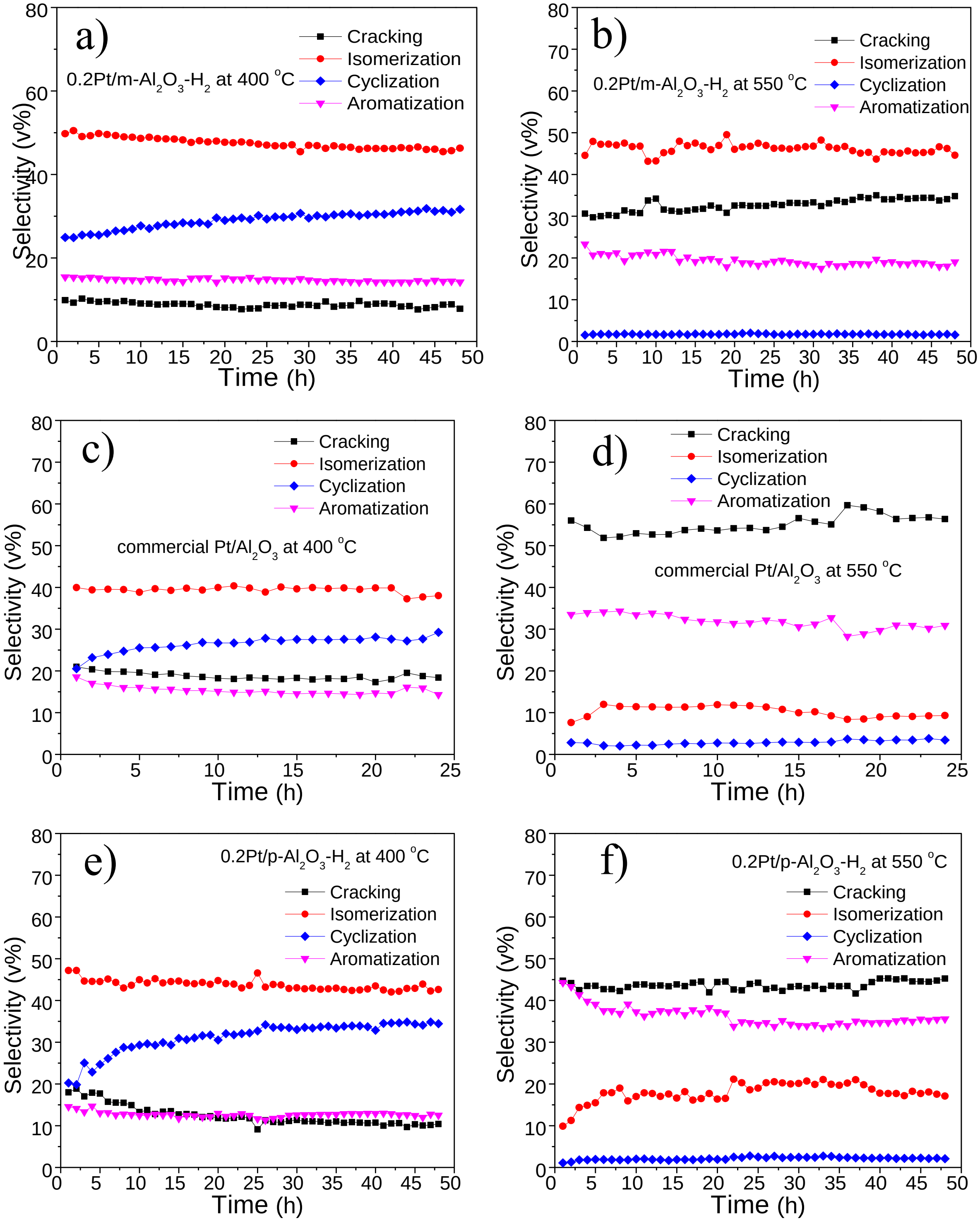

Supplementary Figure 39 | Product selectivity for *n*-hexane reforming reaction over different catalysts. (**a**) 0.2Pt/m-Al_2O_3-H_2 at 400 °C and (**b**) at 550 °C; (**c**) commercial Pt/Al_2O_3 at 400 °C and (**d**) at 550 °C (24 mg 5 wt% Pt/Al_2O_3 catalyst was diluted with 276 mg commercial Al_2O_3); (**e**) 0.2Pt/p-Al_2O_3-H_2 at 400 °C and (**f**) at 550 °C. The reaction was conducted with a 0.1 ml h^{-1} *n*-hexane and 6 ml min^{-1} pure H_2 at ambient pressure.

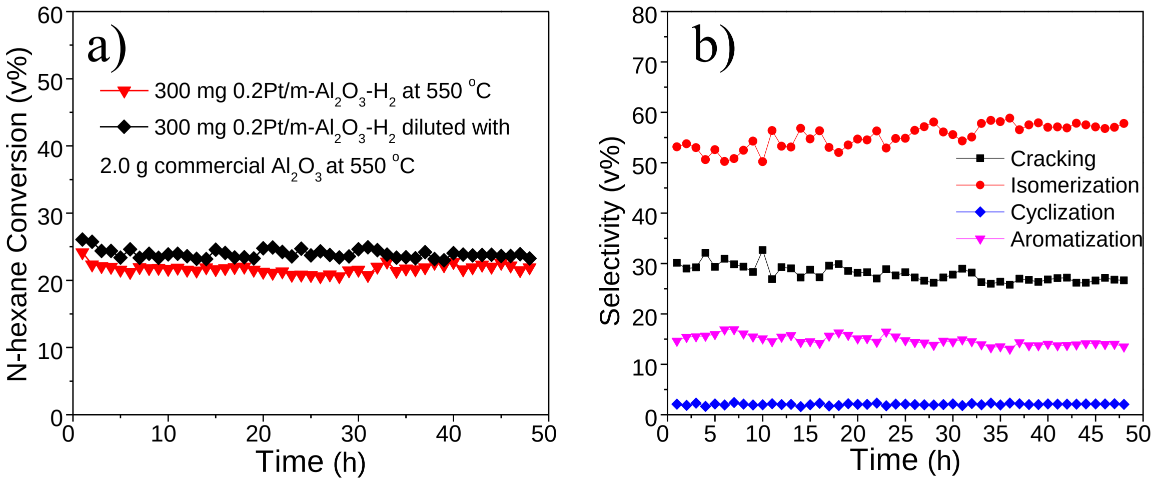

Supplementary Figure 40 | (a) The conversion of *n*-hexane and (**b**) products selectivity over 300 mg 0.2Pt/m-Al_2O_3-H_2 diluted with 2.0 g commercial Al_2O_3 at 550 °C. The reaction was conducted with a 0.1 ml h^{-1} *n*-hexane and 6 ml min^{-1} pure H_2 at ambient pressure. For comparison, *n*-hexane conversion over pure 0.2Pt/m-Al_2O_3-H_2 catalyst was also shown in (**a**), in red line.

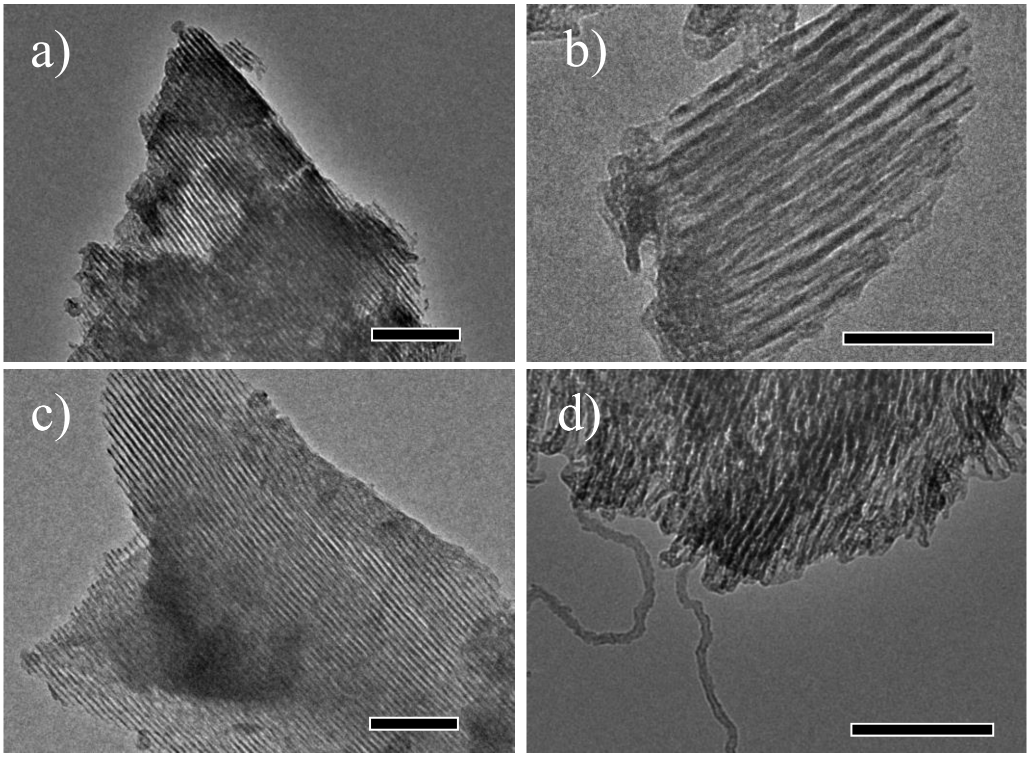

Supplementary Figure 41 | TEM images of 0.2Pt/m-Al_2O_3-H_2 catalyst after *n*-hexane reforming. (**a,b**) 400 °C for 48 h and (**c,d**) 550 °C for 48 h. Scale bar, 200 nm **(a,c,d)**, 100 nm **(b)**. TEM images of 0.2Pt/m-Al_2O_3-H_2 catalyst after *n*-hexane reforming indicate that no obvious Pt nanoparticle formation and carbon deposition after reaction at 400 °C for 48 h and some carbon nanotube formation after reaction at 550 °C for 48 h.

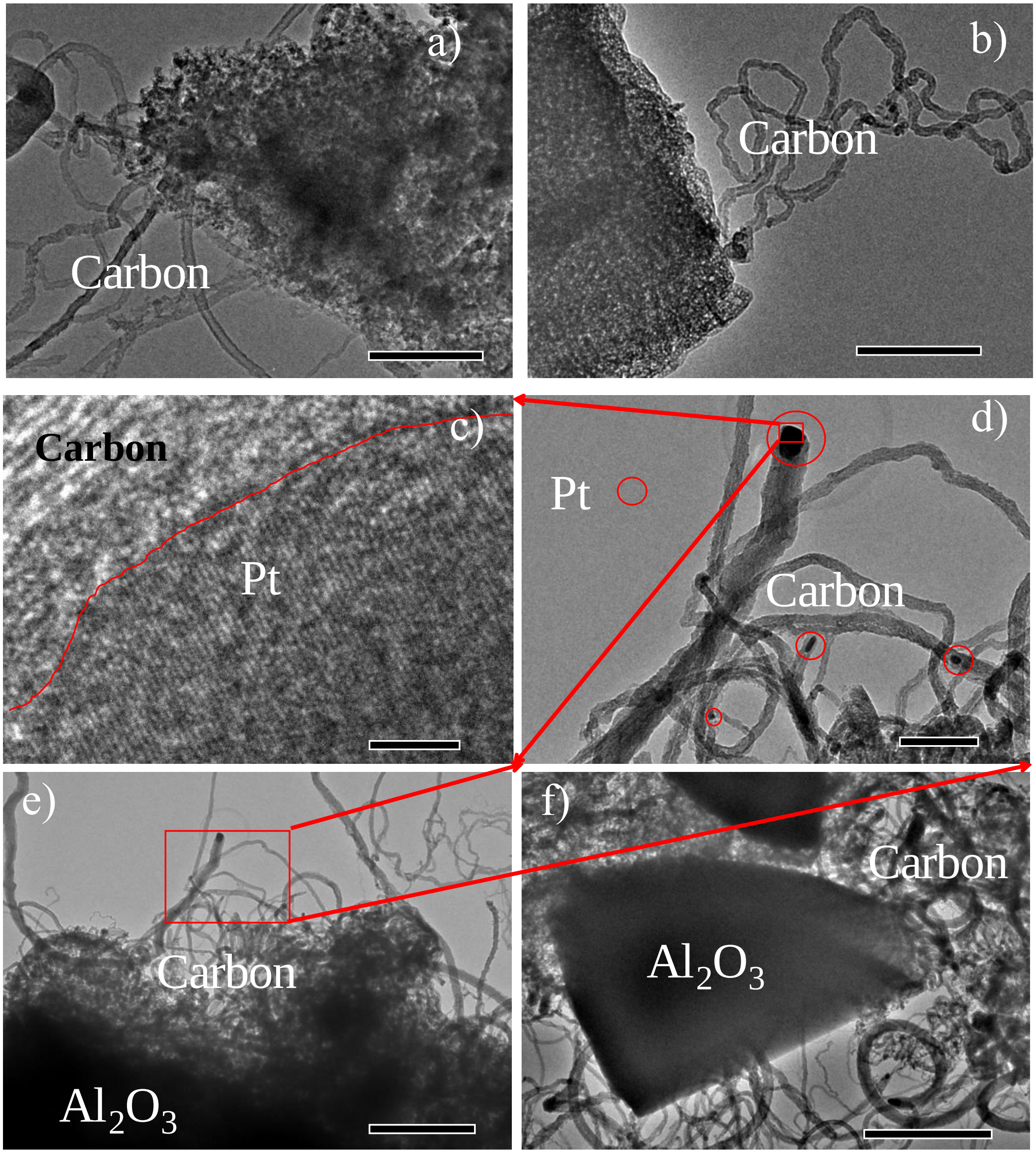

Supplementary Figure 42 | TEM images of 0.2Pt/p-Al_2O_3-H_2 catalyst after *n*-hexane reforming reaction. (**a,b**) 400 °C for 48 h and (**c,d,e,f**) 550 °C for 48 h. Scale bar, 500 nm **(a)**, 200 nm **(b)**, 2 nm **(c)**, 200 nm **(d)**, 1μm **(e,f)**. Surprisingly, a number of carbon nanotubes were identified on 0.2Pt/p-Al_2O_3-H_2 catalyst after *n*-hexane reforming at 400 °C for 48 h. Similarly, considerable carbon deposition and Pt nanoparticle formation were observed after *n*-hexane reforming at 550 °C for 48 h.

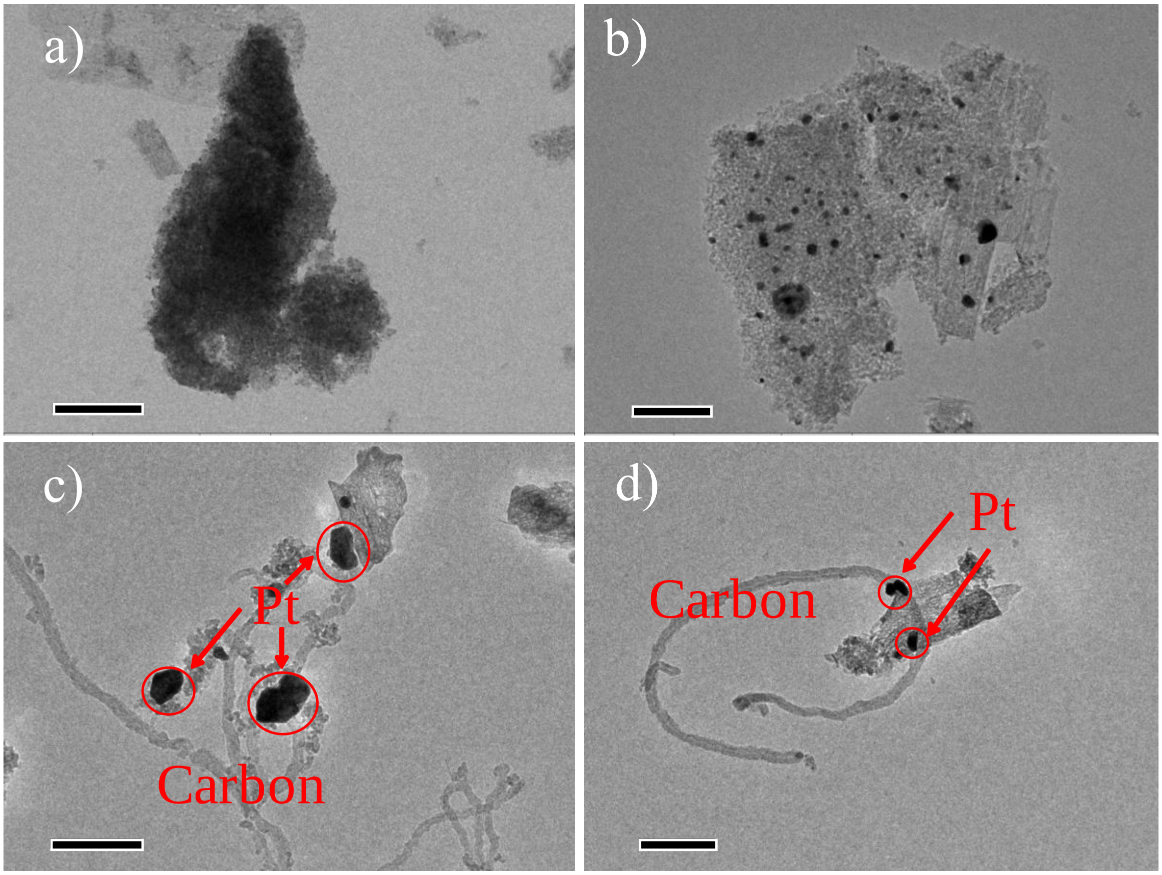

Supplementary Figure 43 | TEM images of commercial Pt/Al_2O_3 catalyst. (**a**) fresh catalyst, (**b**) spent catalyst after *n*-hexane reforming reaction at 400 °C for 24 h, and (**c,d**) at 550 °C for 24 h. Scale bar, 200 nm. Compared with the fresh catalyst, large Pt particles were formed after *n*-hexane reforming at 400 °C. In addition to this, significant carbon nanotubes formation occurred after *n*-hexane reforming at 550 °C.

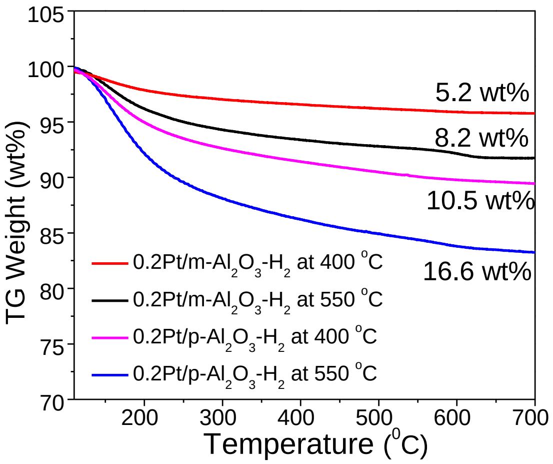

Supplementary Figure 44 | TG curves of the 0.2Pt/m-Al_2O_3-H_2 and 0.2Pt/p-Al_2O_3-H_2 after *n*-hexane reforming at 400 °C and 550 °C for 48 h. Measuring condition: air; 100°C-700 °C; samples were pre-treated in air at 100 °C for 10 min. The TG results indicate that the 0.2Pt/p-Al_2O_3-H_2 catalyst after *n*-hexane reforming at 400 °C and 550 °C induced more carbon deposition than Pt/m-Al_2O_3-H_2 single-atom catalyst, consistent with the above TEM observation.

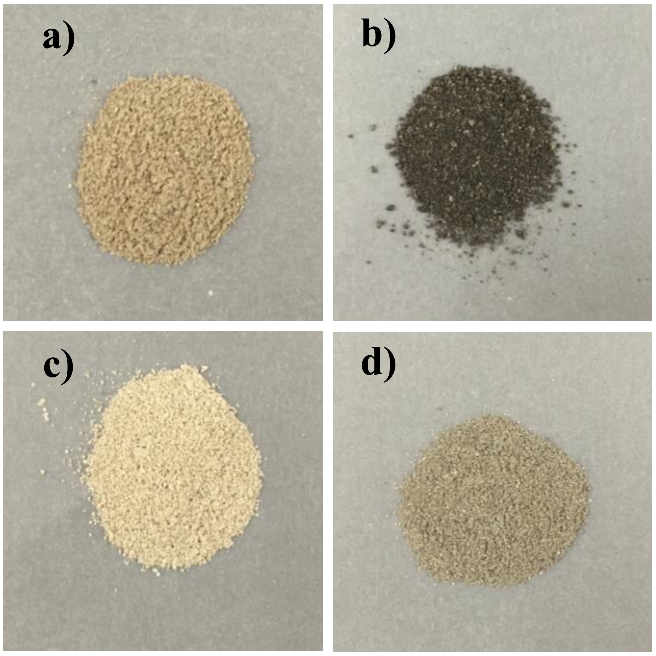

Supplementary Figure 45 | Photos of (**a,c**) 0.2Pt/m-Al_2O_3-H_2 and (**b,d**) 0.2Pt/p-Al_2O_3-H_2 catalysts after *n*-hexane reforming at (**a,b**) 550 °C and (**c,d**) 400 °C for 48 h. Compared with the light black color of 0.2Pt/m-Al_2O_3-H_2 catalyst after *n*-hexane reforming reaction, the dark black color of 0.2Pt/p-Al_2O_3-H_2 catalyst after *n*-hexane reforming reaction were found, indicating much less carbon deposition on 0.2Pt/m-Al_2O_3-H_2 catalyst.

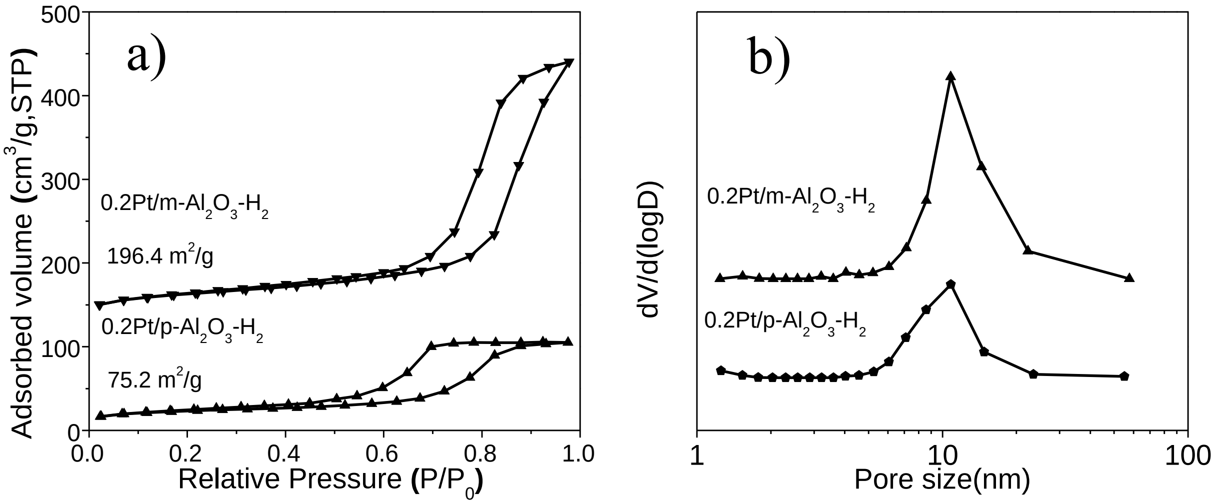

Supplementary Figure 46 | (a) N_2 adsorption-desorption isotherms, and (**b**) their PSD curves of 0.2Pt/m-Al_2O_3-H_2, and 0.2Pt/p-Al_2O_3-H_2 after *n*-hexane reforming at 550 °C for 48 h (sample 0.2Pt/m-Al_2O_3-H_2 was shifted upwards for 150 cm^3 g^{-1}). The surface area of 0.2Pt/p-Al_2O_3-H_2 decreased considerably, in sharp contrast with 0.2Pt/m-Al_2O_3-H_2 which only underwent slight decrease of surface area after *n*-hexane reforming at 550 °C for 48 h.

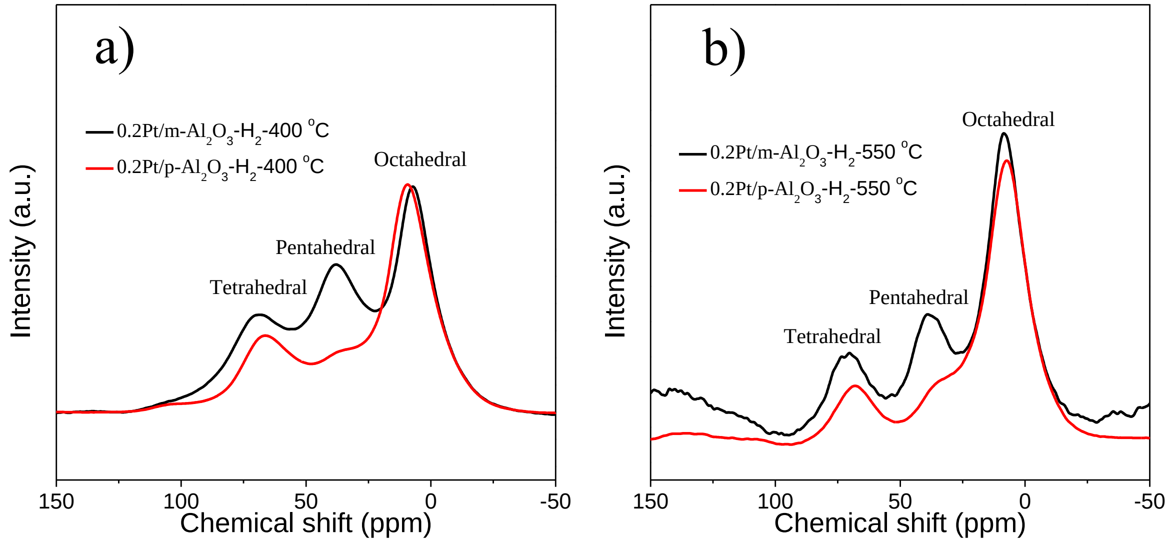

Supplementary Figure 47 | The ^{27}Al MAS-NMR spectra of spent catalysts. 0.2Pt/m-Al_2O_3-H_2 and 0.2Pt/p-Al_2O_3-H_2 after *n*-hexane reforming at (**a**) 400 °C for 48 h and (**b**) 550 °C for 48 h. A majority of pentahedral Al^{3+} centers in 0.2Pt/m-Al_2O_3-H_2 spent catalyst remained after the reaction, while most pentahedral Al^{3+} centers in 0.2Pt/p-Al_2O_3-H_2 catalyst disappeared after *n*-hexane reforming.

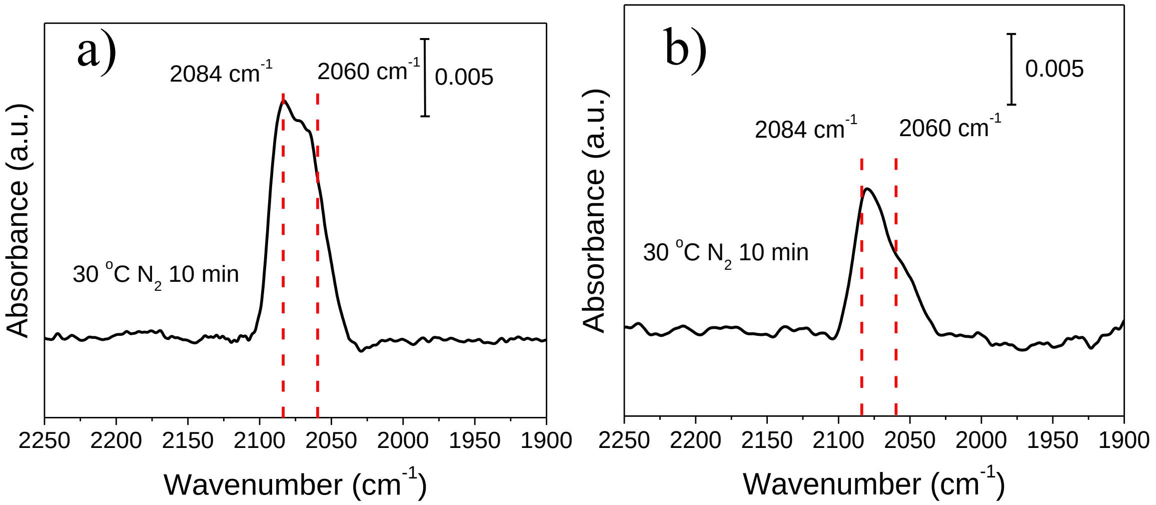

Supplementary Figure 48 | IR spectra of CO adsorbed on 0.2Pt/m-Al_2O_3-H_2 catalyst after *n*-hexane reforming. After 48 h reaction at (**a**) 400 °C and (**b**) 550 °C. All spectra were collected after CO absorption followed by N_2 purging for 10 min.

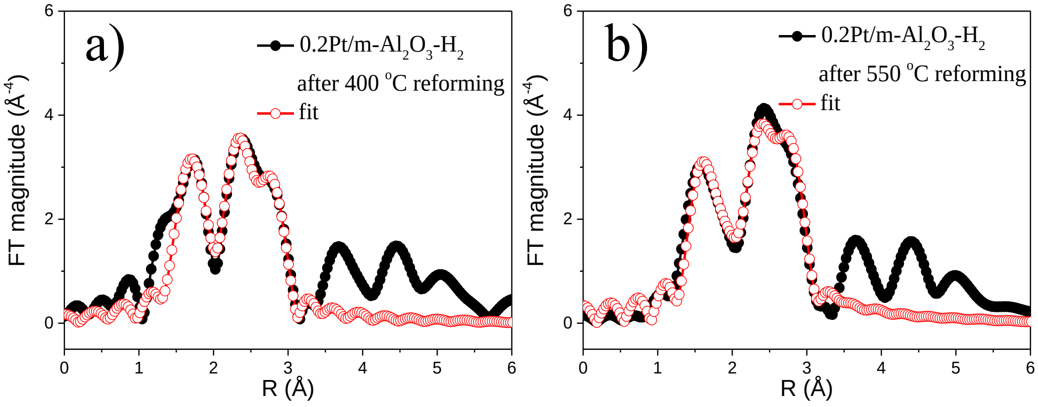

Supplementary Figure 49 | FT-EXAFS curves and fitting for 0.2Pt/m-Al_2O_3-H_2 catalyst after *n*-hexane reforming. After 48 h reaction at (**a**) 400 °C and (**b**) 550 °C.

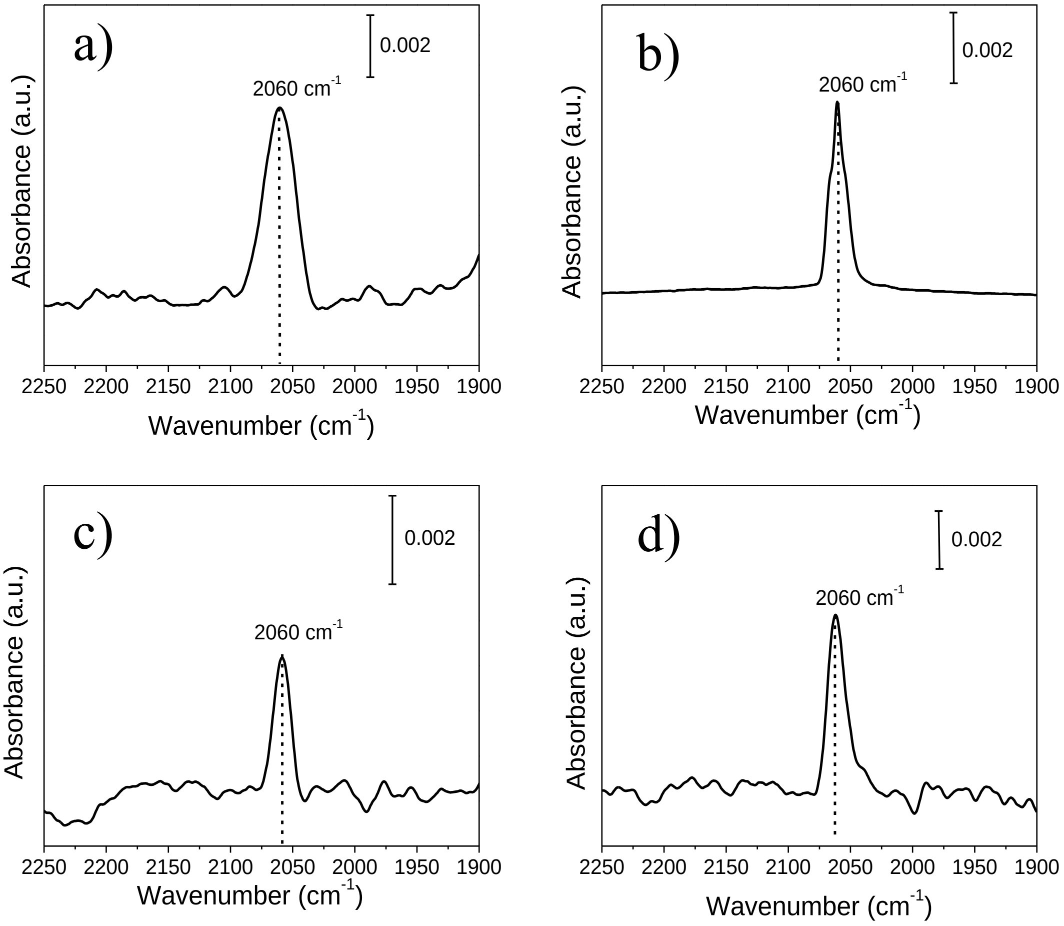

Supplementary Figure 50 | IR spectra of CO adsorption for commercial Pt/Al_2O_3 and 0.2Pt/p-Al_2O_3-H_2 catalysts after *n*-hexane reforming reaction. Commercial Pt/Al_2O_3 for *n*-hexane reforming at (**a**) 400 ^oC and (**b**) 550 ^oC after 24 h reaction; 0.2Pt/p-Al_2O_3-H_2 catalyst for *n*-hexane reforming at (**c**) 400 ^oC and (**d**) 550 ^oC after 48 h reaction. All spectra were collected after CO absorption followed by N_2 purging for 10 min. The band at about 2060 cm^{-1} is ascribed to the linearly bonded CO on Pt^0 sites.

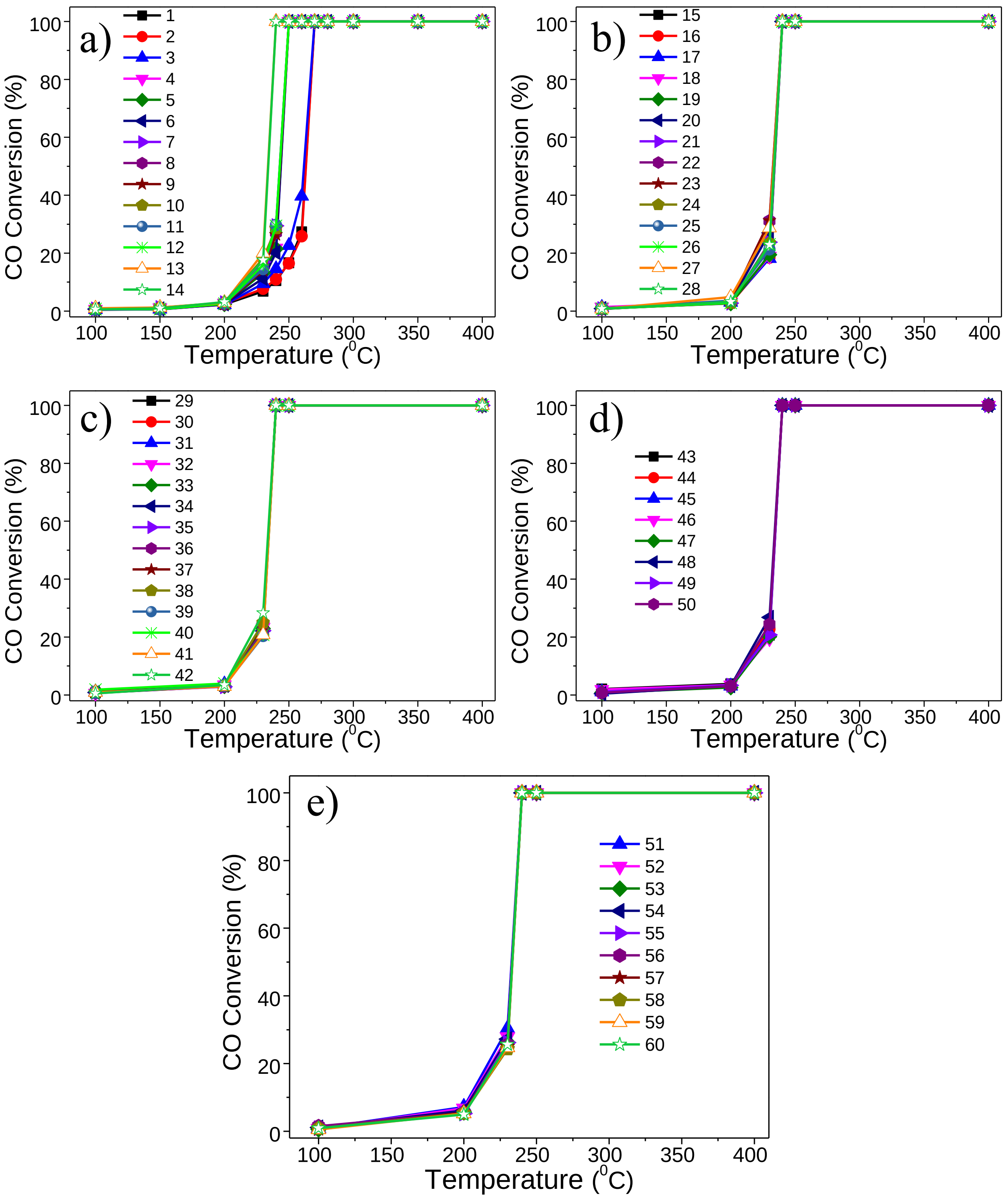

Supplementary Figure 51 | CO conversion of over 0.2Pt/m-Al_2O_3-H_2 between 100-400 °C. (**a**) 1st-14th cycles, (**b**) 15th-28th cycles, (**c**) 29th-42nd cycles, (**d**) 43th-50th cycles, and (**e**) 50th-60th cycles. The feed gas containing 2.5 vol% CO, 2.5 vol% O_2 and balance Ar was allowed to pass through the reactor at a flow rate of 80 ml min^{-1}.

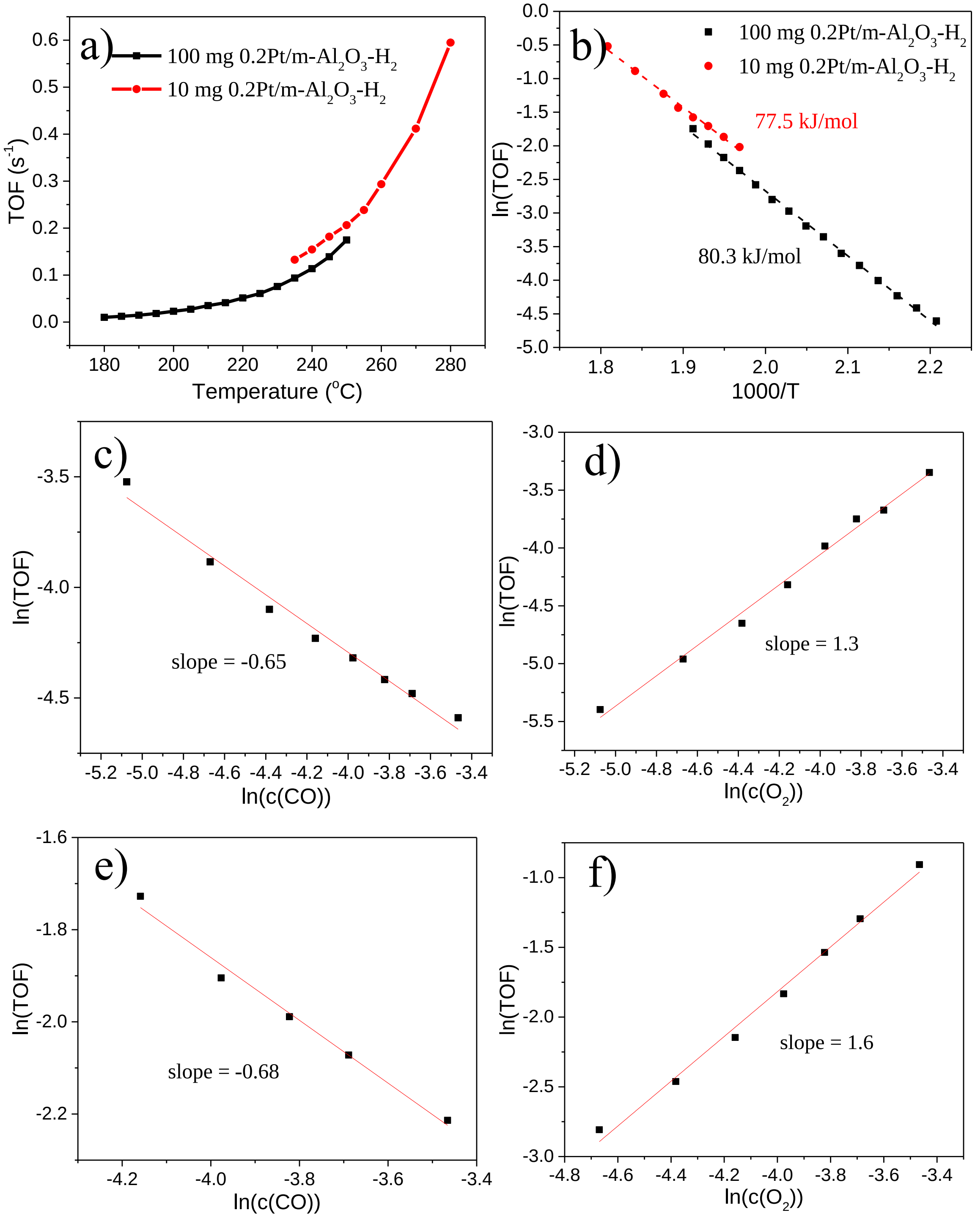

Supplementary Figure 52 | CO oxidation kinetics over 0.2Pt/m-Al_2O_3-H_2. (**a**) TOF as a function of reaction temperature. (**b**) Arrhenius plots. (**c**) CO and (**d**) O_2 order measured at 195 °C over 100 mg 0.2Pt/m-Al_2O_3-H_2. (**e**) CO and (**f**) O_2 order measured at 250 °C over 10 mg 0.2Pt/m-Al_2O_3-H_2. Catalyst was diluted 10 times with pure Al_2O_3. The apparent activation energy for CO oxidation between 180-250 °C was 80.3 kJ/mol, whereas it was 77.5 kJ/mol between 235-280 °C (Supplementary Fig. 52). This is different from the classic scenario of CO oxidation over Pt nanoparticles where a two-stage reaction

mechanism was often observed. O_2 displayed a positive order (ca. +1.3-1.6) while CO displayed a negative order (ca. -0.6) at both lower temperature (195 °C) and higher temperature (250 °C), which also suggests a uniform reaction mechanism. In a recent report, water reacts with CeO_2 to form surface hydroxyl groups, enabling a new pathway for CO oxidation over single-atom Pt catalyst. In our system, the TOF of the catalyst at 150, 200, and 250 °C were 0.012, 0.039, and 0.26 s^{-1} without adding water, and 0.015, 0.043, and 0.22 s^{-1}, respectively, in the presence of 1.25% water, indicating such a promotional effect does not apply to the current system.

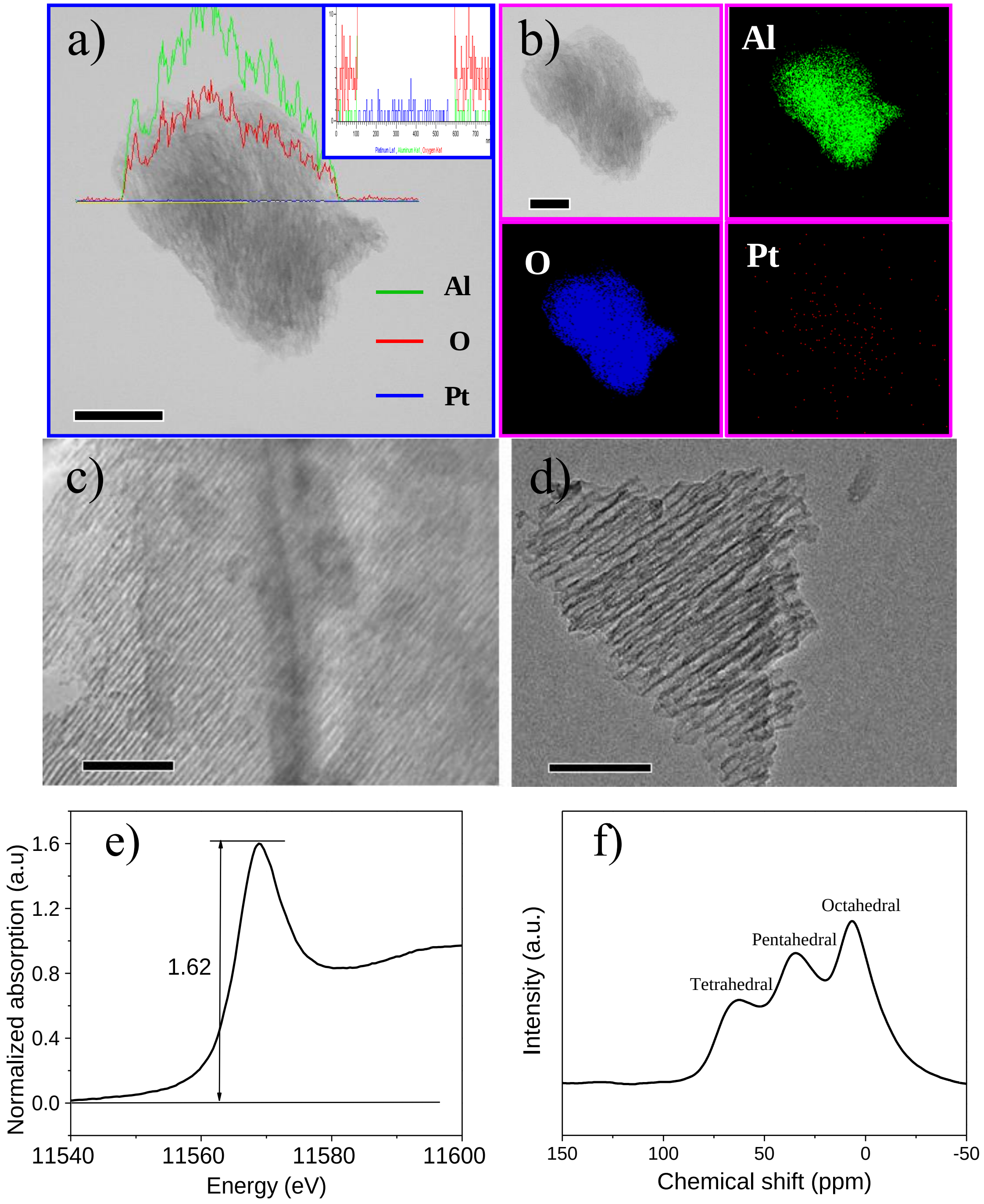

Supplementary Figure 53 | Characterization of spent 0.2Pt/m-Al_2O_3-H_2 catalyst after CO oxidation. (**a**) TEM image and the line scan along the yellow line (inset is the enlarge line scan along the yellow line), (**b**) TEM elemental mapping images, **(c,d**) additional TEM images, (**e**) normalized XANES spectra at the Pt L_3-edge, and (**f**) the ^{27}Al MAS-NMR spectrum. Before these characterizations, 0.2Pt/m-Al_2O_3-H_2 catalyst was subjected to the following test sequence: 1) CO oxidation for 50 cycles between 100 and 400 °C, 2) maintained at 400 °C for 220 h, 3) 51st to 60th cycles between 100 and 400 °C, and 4) maintained at 230 °C for 70 h. Scale bar, 200 nm (**a**), 100 nm (**b,c,d**).

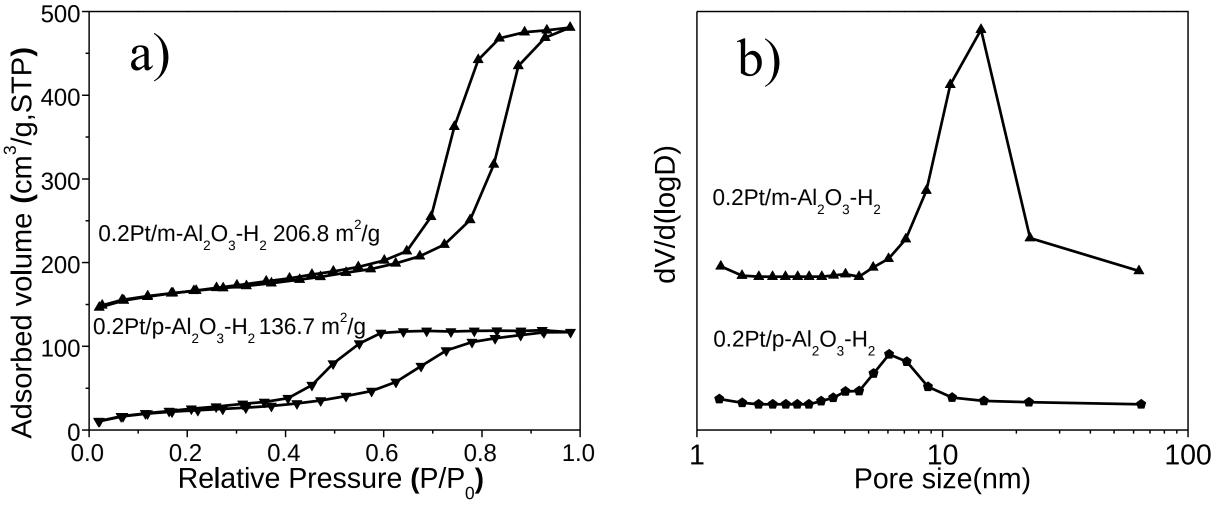

Supplementary Figure 54 | (**a**) N_2 adsorption-desorption isotherms and (**b**) their PSD curves of 0.2Pt/m-Al_2O_3-H_2, 0.2Pt/p-Al_2O_3-H_2 after CO oxidation reaction (sample 0.2Pt/m-Al_2O_3-H_2 was shifted upwards for 150 cm^3 g^{-1}). Before the measurement, 0.2Pt/m-Al_2O_3-H_2 catalyst was subjected to the following test sequence: 1) CO oxidation for 50 cycles between 100 and 400 °C, 2) maintained at 400 °C for 220 h, 3) 51st to 60th cycles between 100 and 400 °C, and 4) maintained at 230 °C for 70 h, while 0.2Pt/p-Al_2O_3-H_2 catalyst was cycled 14 times between 100 and 400 °C.

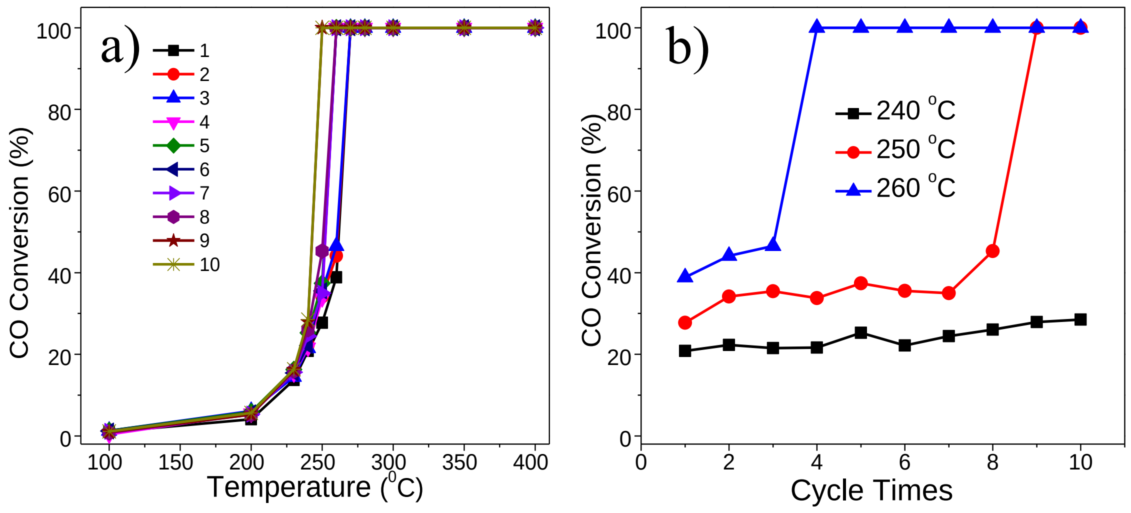

Supplementary Figure 55 | CO oxidation over 0.2Pt/m-Al_2O_3-H_2 (100 mg) diluted in commercial Al_2O_3 (1.0 g). (**a**) CO conversion during 1st-10th cycles from 100-400 °C, and (**b**) CO conversion at various temperatures vs. cycle times. The feed gas containing 2.5 vol% CO, 2.5 vol% O_2 and balance Ar was allowed to pass through the reactor at a flow rate of 80 ml min^{-1}.

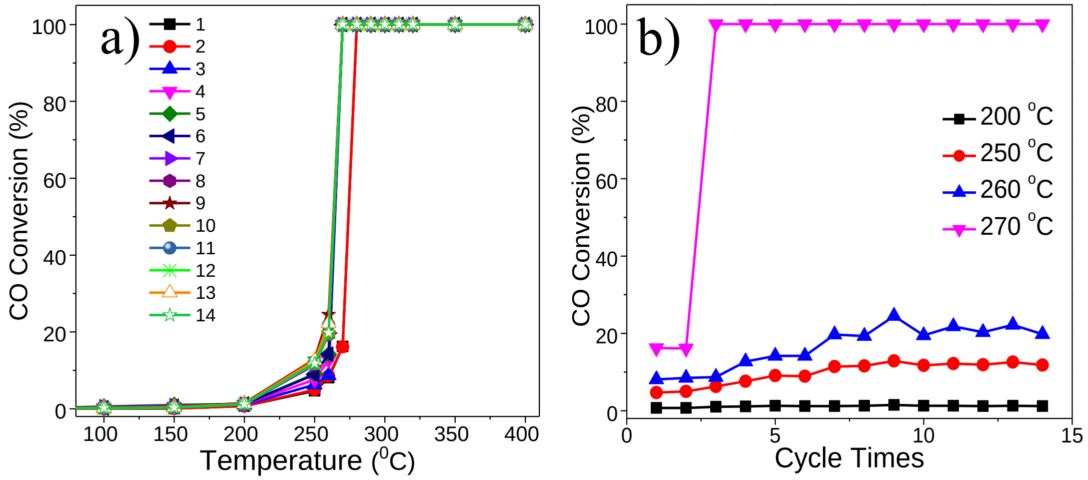

Supplementary Figure 56 | CO conversion over 0.2Pt/m-Al_2O_3-O_2 (**a**) CO conversion during 1st-14th cycles from 100-400 °C and (**b**) CO conversion at various temperatures vs. cycle times. The feed gas containing 2.5 vol% CO, 2.5 vol% O_2 and balance Ar was allowed to pass through the reactor at a flow rate of 80 ml min^{-1}.

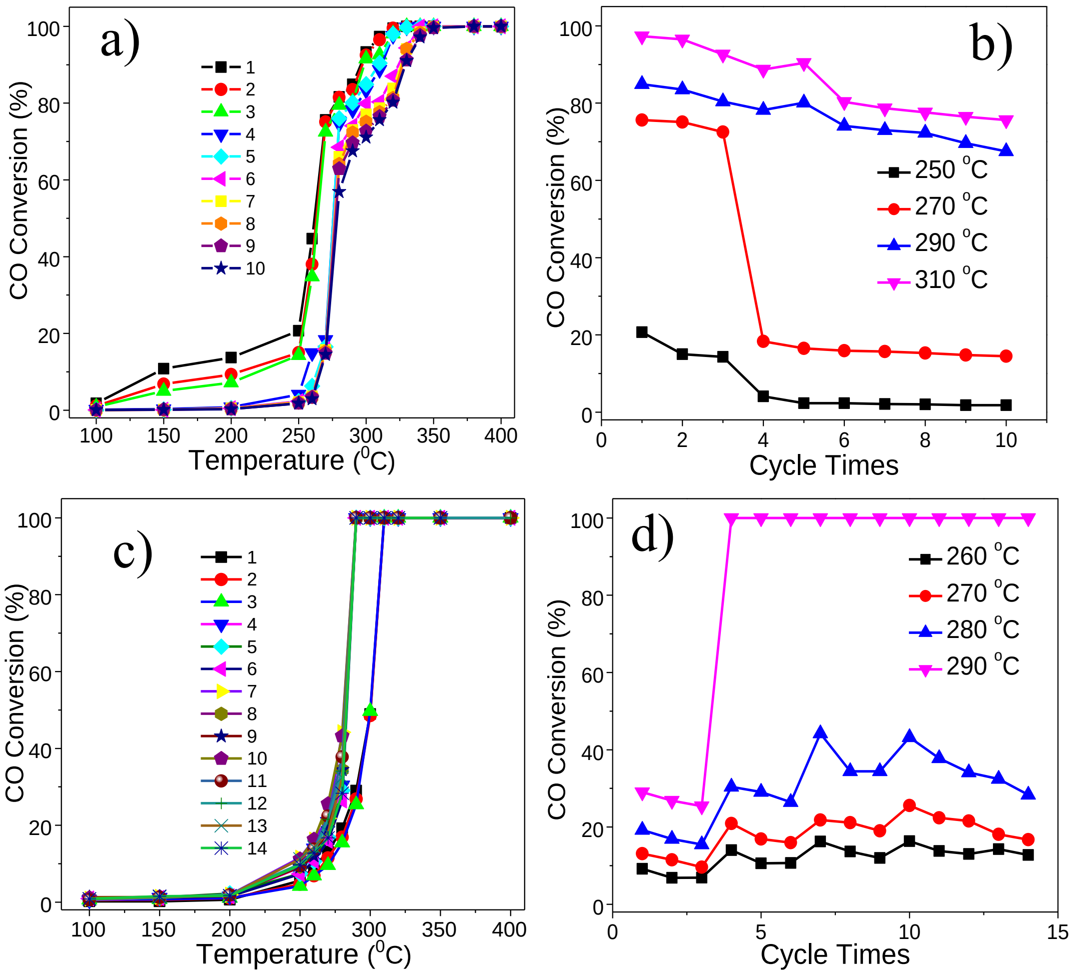

Supplementary Figure 57 | (a,b) CO conversion over commercial Pt/Al_2O_3 catalyst between 100-400 °C (1st-10th cycles), and **(c,d)** CO conversion over 0.2Pt/p-Al_2O_3-H_2 catalyst between 100-400 °C (1st-14th cycles). The feed gas containing 2.5 vol% CO, 2.5 vol% O_2 and balance Ar was allowed to pass through the reactor at a flow rate of 80 ml min^{-1}.

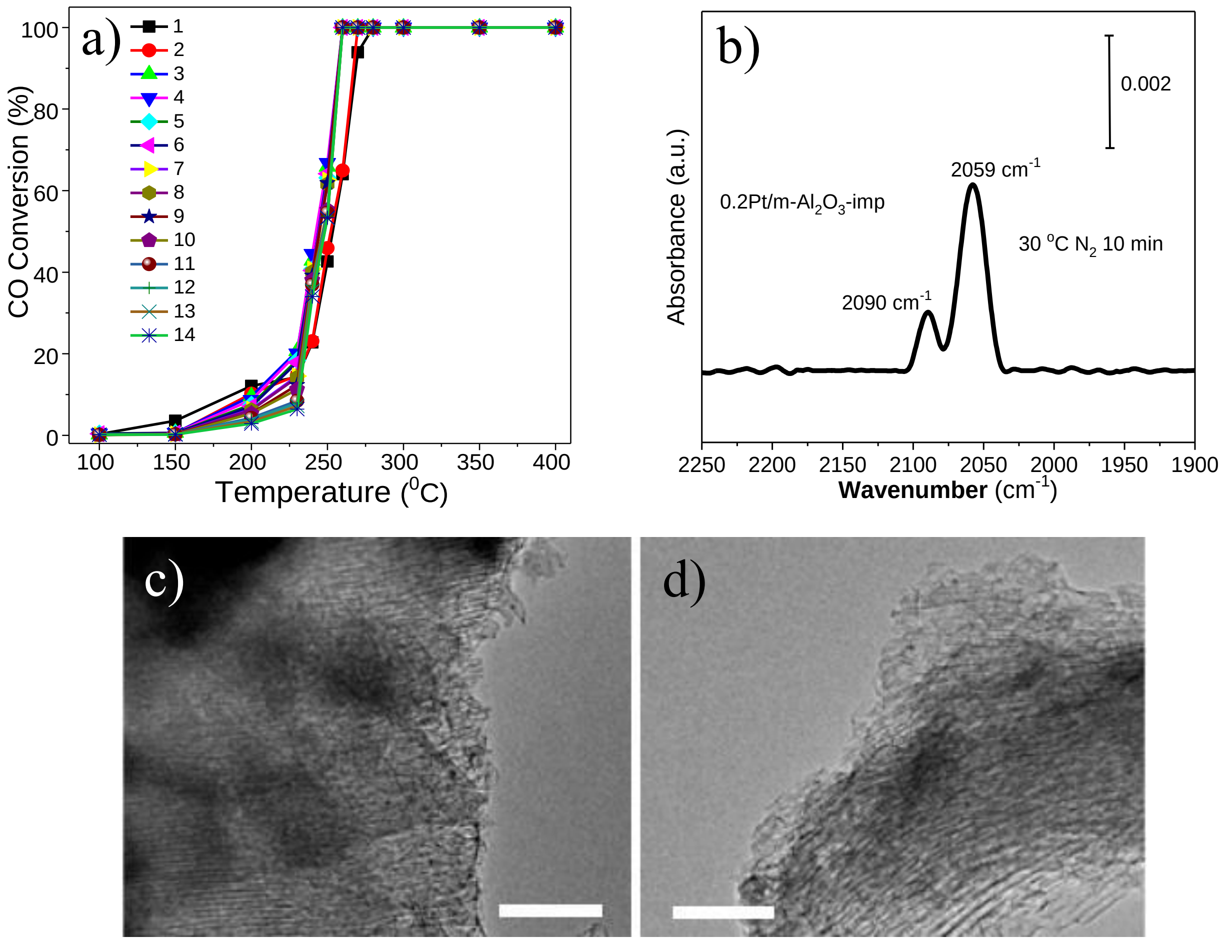

Supplementary Figure 58 | (**a**) CO conversion over 0.2Pt/m-Al_2O_3-imp between 100-400 °C (1st-14th cycles). (**b-d**) Characterization of spent 0.2Pt/m-Al_2O_3-imp catalyst after CO oxidation. (**b**) IR spectra of CO adsorption, and (**c**,**d**) TEM images. Scale bar, 100 nm (**c,d**).

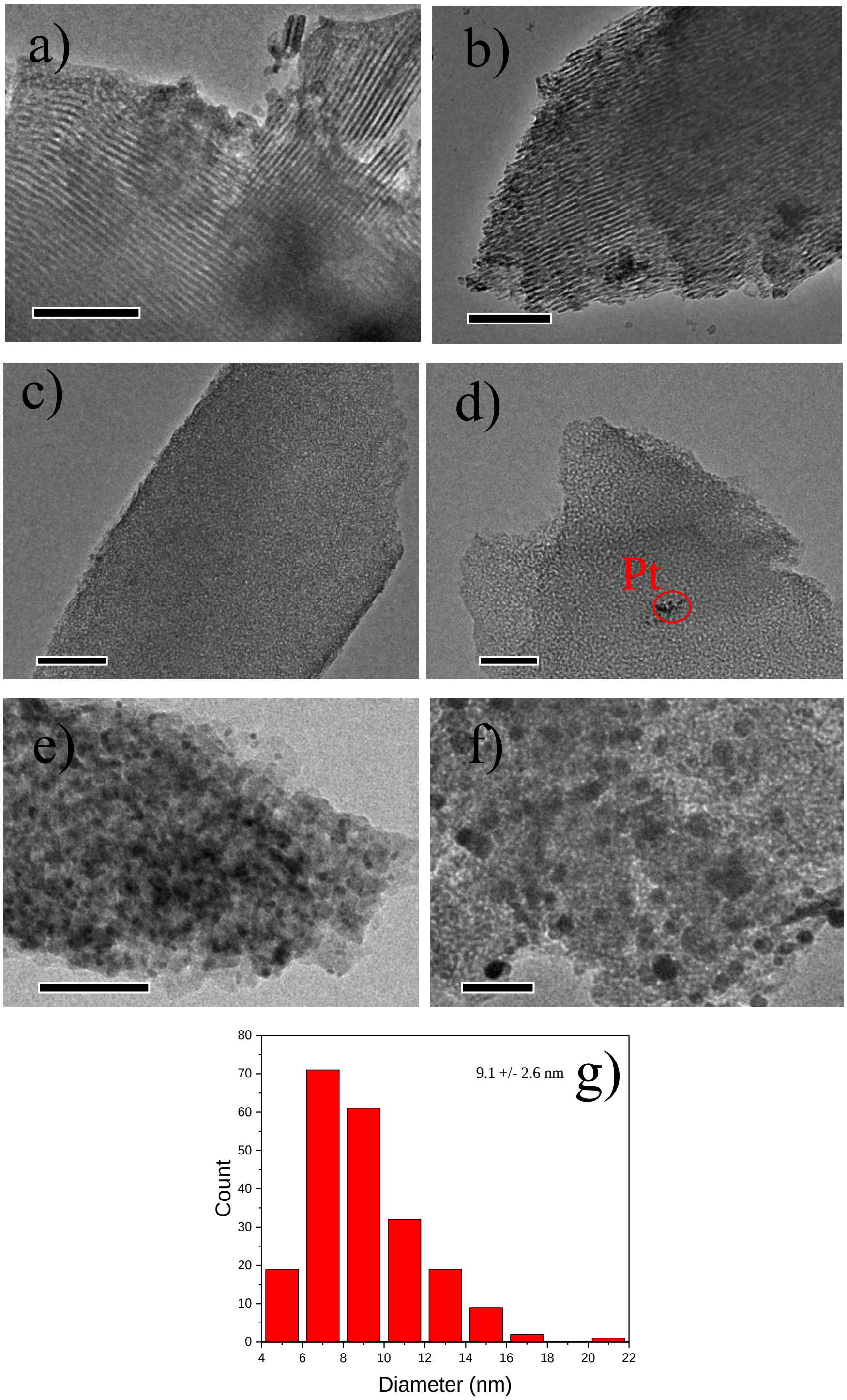

Supplementary Figure 59 | TEM images of catalysts after CO oxidation. (**a,b**) 0.2Pt/m-Al_2O_3-O_2, (**c,d**) 0.2Pt/p-Al_2O_3-H_2 after CO oxidation for 14 cycles, and (**e,f**) commercial Pt/Al_2O_3 after CO oxidation for 10 cycles. (**g**) Size distribution for commercial Pt/Al_2O_3 after CO oxidation for 10 cycles. Scale bar, 100 nm (**a**), 200 nm (**b,c**), 50 nm (**d,e,f**). No obvious Pt nanoparticles were found for 0.2Pt/m-Al_2O_3-O_2 catalyst after CO oxidation reaction. For 0.2Pt/p-Al_2O_3-H_2, some Pt nanoparticles are found in

disordered p-Al_2O_3. For commercial Pt/Al_2O_3, the Pt nanoparticles become larger (9.1 +/- 2.6 nm) than fresh commercial Pt/Al_2O_3 (3.9 +/-0.6 nm) after the reaction.

Supplementary Tables

Supplementary Table 1. ICP-OES analysis of Pt content for all samples.

Samples	Measurement amounts of Pt (wt%)	Stoichiometric amounts of Pt (wt%)
**0.2Pt/m-Al_2O_3-O_2**	0.19	0.2
**0.5Pt/m-Al_2O_3-O_2**	0.49	0.5
**2.0Pt/m-Al_2O_3-O_2**	1.91	2.0
**0.2Pt/m-Al_2O_3-H_2**	0.20	0.2
**0.5Pt/m-Al_2O_3-H_2**	0.48	0.5
**2.0Pt/m-Al_2O_3-H_2**	1.93	2.0
**0.2Pt/p-Al_2O_3-O_2**	0.20	0.2
**0.5Pt/p-Al_2O_3-O_2**	0.49	0.5
**2.0Pt/p-Al_2O_3-O_2**	1.96	2.0
**0.2Pt/p-Al_2O_3-H_2**	0.19	0.2
**0.5Pt/p-Al_2O_3-H_2**	0.48	0.5
**2.0Pt/p-Al_2O_3-H_2**	1.97	2.0
**0.2Pt/m-Al_2O_3-O_2-600**	0.21	0.2
**0.2Pt/m-Al_2O_3-O_2-800**	0.22	0.2

Supplementary Table 2. Organic elemental analysis of the samples

Sample	C (wt%)	H (wt%)	N (wt%)
**0.2Pt/m-Al_2O_3-O_2**	0.79	2.31	0.97
**0.2Pt/m-Al_2O_3-H_2**	Not detected	1.48	<0.50

Supplementary Table 3. Surface area, pore volume, and the maximum pore size of the samples.

Samples	Surface area[a] (m^2 g^{-1})	Vp[b] (cm^3 g^{-1})	The maximum pore size[c] (nm)
**2.0Pt/m-Al_2O_3-O_2**	218.4	0.53	10.8
**2.0Pt/m-Al_2O_3-H_2**	200.6	0.52	10.7
**0.5Pt/m-Al_2O_3-O_2**	217.4	0.56	10.8
**0.5Pt/m-Al_2O_3-H_2**	210.4	0.51	10.8
**0.2Pt/m-Al_2O_3-O_2**	227.3	0.56	10.7
**0.2Pt/m-Al_2O_3-H_2**	213.1	0.53	10.7

[a]Surface area derived from BET equation.

[b]Pore volume obtained from the volume of nitrogen adsorbed at the relative pressure of 0.98.

[c]The pore size at the maximum position derived from BJH method using adsorption branch.

Supplementary Table 4. EXAFS parameters of Pt foil, 0.2Pt/m-Al_2O_3-O_2, and 0.2Pt/m-Al_2O_3-H_2 and spent catalysts. C.N., coordination number; r, bond length; σ^2, the Debye–Waller factor; ΔE_0, inner potential correction to account for the difference in the inner potential between the sample and each FEFF simulated path. $\Delta k = 1.3 – 2.0^{*1,*2,*4}$; $1.7 – 3.2^{*3}$; $1.3 – 2.1^{*5}$; $1.4 – 3.4^{*6,*7}$

**Samples**	**Shell**	**C.N.**	**r/Å**	**σ^2**	**ΔE_0 (eV)**	**White line intensity**
**0.2Pt/m-Al_2O_3-O_2 *1**	Pt-O	3.8+/-1.8	2.023 +/-0.033	0.0053 +/-0.0044	1.6+/-5.7	1.66
**0.2Pt/m-Al_2O_3-H_2 *2**	Pt-O	4.0+/-1.2	2.009 +/-0.022	0.0055 +/-0.0029	-0.7+/-3.9	1.65
**Pt foil *3**	Pt-Pt	12 (fixed)	2.766 +/-0.002	0.0046 +/-0.0002	0.7+/-0.5	1.25
**PtO_2 *4**	Pt-O	6 (fixed)	2.016 +/-0.008	0.0028 +/-0.0005	0.0+/-1.6	2.20
**0.2Pt/m-Al_2O_3-H_2 *5** After CO oxidation	Pt-O	3.6+/-0.9	2.024 +/-0.019	0.0063 +/- 0.0026	6.6+/-3.3	1.63
**0.2Pt/m-Al_2O_3-H_2 *6** After reforming at 400 °C	Pt-O	1.3+/-0.4	2.056 +/-0.017	0.0024 +/-0.0022	8.1+/-3.7	1.46
	Pt-Pt	6.5+/1.6	2.726 +/-0.015	0.0095 +/-0.0015	-6.6+/-3.3	
**0.2Pt/m-Al_2O_3-H_2 *7** After reforming at 550 °C	Pt-O	1.5+/-0.4	2.025 +/-0.015	0.0035 +/-0.0020	1.8+/-3.1	1.46
	Pt-Pt	5.6+/-1.0	2.732 +/-0.009	0.0076 +/-0.0010	-4.1+/-2.0	

Supplementary Table 5. Hydrogenation of acetophenone over 0.2Pt/m-Al_2O_3-H_2 and control catalysts.

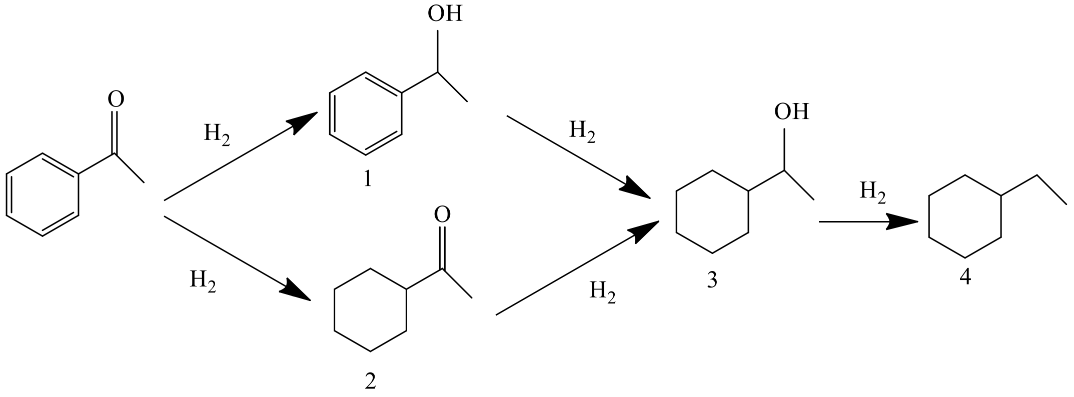

Catalyst	Conversion /%	Selectivity of 1/%	Selectivity of 2/%	Selectivity of 3/%	Selectivity of 4/%
**0.2Pt/m-Al_2O_3-H_2**	69.3	98.7	1.1	0.2	0
**0.2Pt/p-Al_2O_3-H_2**	53.1	78.9	20.0	1.1	0
**commercial Pt/Al_2O_3**	82.3	68.3	4.4	3.7	23.6

Reaction conditions: 1.0 mL methanol, 0.2 mmol acetophenone, Pt : acetophenone = 1:500 (molar), 1.0 MPa of H_2, 50 ^oC, 12 h.

Supplementary Table 6. Hydrogenation of phenylacetylene over 0.2Pt/m-Al_2O_3-H_2 and control catalysts.

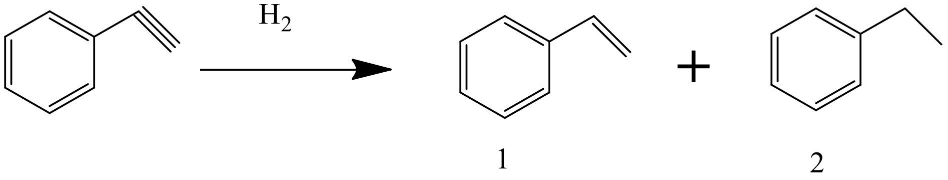

Catalyst	Conversion/%	Selectivity of 1/%	Selectivity of 2/%
**0.2Pt/m-Al_2O_3-H_2**	63.8	95.7	4.3
**0.2Pt/p-Al_2O_3-H_2**	55.1	88.0	12.0
**commercial Pt/Al_2O_3**	48.2	66.4	33.6

Reaction conditions: 1.0 mL methanol, 0.2 mmol phenylacetylene, Pt : phenylacetylene = 1:1000 (molar), 1.0 MPa of H_2, 25 ^oC, 30 min.

Supplementary Table 7. Hydrogenation of nitrobenzene over 0.2Pt/m-Al_2O_3-H_2 and control catalysts.

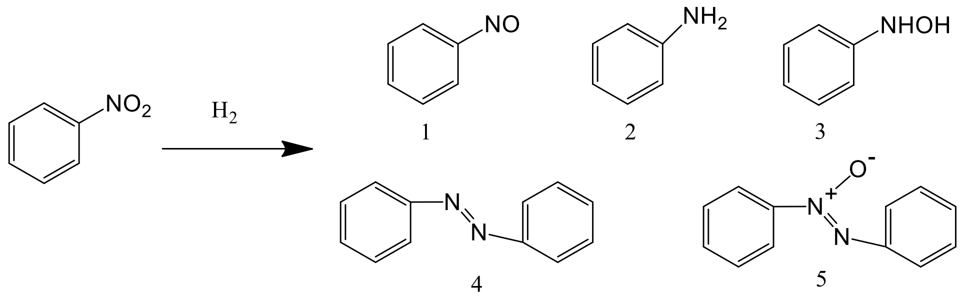

Catalyst	Conversion/%	Selectivity of 1/%	Selectivity of 2/%	Selectivity of 3/%	Selectivity of 4/%	Selectivity of 5/%
**0.2Pt/m-Al_2O_3-H_2**	99.8	0	100	0	0	0
**0.2Pt/p-Al_2O_3-H_2**	69.1	3.0	72.4	0	2.3	22.3
**commercial Pt/Al_2O_3**	72.5	1.1	62.0	0	2.7	34.2

Reaction conditions: 1.0 mL methanol, 0.2 mmol nitrobenzene, Pt : nitrobenzene = 1:2000 (molar), 1.0 MPa of H_2, 25 ^oC, 30 min.

Supplementary Table 8. A comparison of activity in selective hydrogenation of 1,3-butadiene on various catalysts.

Catalysts	Dispersion[a]	Buta/H_2	T (°C)	Conversion (%)	Butenes selectivity (%)	ATOF (s^{-1})	TOF (s^{-1})	Notes
**0.2Pt/m-Al_2O_3-H_2**	100%	1:8	30	25.8	100	0.034	0.034	This work
**0.2Pt/m-Al_2O_3-H_2**	100%	1:8	40	51.3	100	0.068	0.068	This work
**0.2Pt/m-Al_2O_3-H_2**	100%	1:8	50	92.6	100	0.12	0.12	This work
**0.2Pt/p-Al_2O_3-H_2**	82%	1:8	30	34	79	0.045	0.055	This work
**Commercial Pt/Al_2O_3**	41%	1:8	30	55	42	0.072	0.18	This work
**Pt_1@Cu/Al_2O_3**	100%	1:16	50	27	100	0.011	0.011	8
**Pt/SiO_2-N**	NR[b]	1:4	0	3	100	0.001	NR[b]	9
**Pt@ZSBA**	NR[b]	NR[b]	40	87	90	2.8	NR[b]	10
**Pt/γ-Al_2O_3**	46%	1:2	25	10	69	0.26	0.57	11
**Pt/Al_2O_3**	75%	1:3	60	NR[b]	76	0.24	0.32	12
**Pt/MgO**	5%	1:3	60	NR[b]	77.2	0.035	0.7	12
**Pt/SiO_2-Al_2O_3**	22%	1:3	60	NR[b]	64.4	0.14	0.65	12

[a]Dispersions in this work were calculated based on H_2-O_2 experiments. For 0.2Pt/m-Al_2O_3-H_2, 100% dispersion was used, although H_2 consumption was more than the expected value of 100% dispersion due to hydrogen spill over. [b]Not reported.

Supplementary Table 9. A comparison of activity in CO oxidation reaction on different Pt catalysts in this work.

Catalysts	Pt dispersion(%)[a]	T (°C)	Conversion (%)	TOF (s^{-1})
**0.2Pt/m-Al_2O_3-H_2 (first round test)**	100	150	0.23	0.003
		160	0.34	0.005
		170	0.51	0.007
		180	0.75	0.010
		190	1.10	0.015
		200	1.72	0.023
		210	2.63	0.035
		220	3.86	0.051
		230	5.70	0.076
		240	8.57	0.114
		250	13.2	0.174
**0.2Pt/p-Al_2O_3-H_2 (first round test)**	82	150	0.20	0.003
		200	0.65	0.011
		250	5.59	0.091
		260	9.19	0.149
		270	13.1	0.212
**Commercial Pt/Al_2O_3 (first round test)**	41	100	1.80	0.058
		150	10.8	0.350
		200	13.7	0.443

[a]Dispersion here refers to the percentage of the number of exposed surface Pt atoms to the total number of Pt atoms in the catalyst, and in this work was calculated based on H_2-O_2 experiments. For 0.2Pt/m-Al_2O_3-H_2, 100% dispersion was used, although H_2 consumption was more than 100% due to hydrogen spill over.

Supplementary Table 10. A comparison of activity in CO oxidation reaction on various supported Pt single-atom catalysts based on the first catalytic cycle.

Catalysts	Pt loading (wt%)	CO/O_2	T (°C)	Conversion (%)	TOF (s^{-1})	Notes
**0.2Pt/m-Al_2O_3-H_2**	0.2	2.5% CO<br>2.5% O_2<br>95% Ar	200<br>250	1.72<br>13.2	0.023<br>0.175	This work
**0.2Pt/p-Al_2O_3-H_2**	0.2	2.5% CO<br>2.5% O_2<br>95% Ar	200	2.2	0.029	This work
**Pt/FeO_x**	0.17	1% CO<br>1% O_2<br>98% He	27	NR[a]	0.136	3
**Pt/θ-Al_2O_3**	0.18	3.7% CO<br>3.7% O_2<br>93.6% He	200<br>251	1.4<br>~10	0.013<br>0.187	4
**$Pt(NH_3)_4^{2+}$/KLTL**	1.0	1% CO<br>1% O_2<br>98% He	150	NR	0.0038	5
**PtO_x/KLTL**	1.0	1% CO<br>1% O_2<br>98% He	150	NR	0.012	5
**Pt/Al_2O_3+Polyhedra Ceria aged 10 h**	1.0	1.9% CO<br>1.3% O_2<br>96.8% Ar	225	15.3	0.17	6
**Pt/Al_2O_3+Polydedra Ceria aged 1 week**	1.0	1.9% CO<br>1.3% O_2<br>96.8% Ar	225	15.4	0.15	6
**Pt/CeO_2**	0.22	1% CO<br>20% O_2<br>79% Ar	110	~10	0.018	7

[a]Not reported.

Supplementary references

1. Sbalzarini, I. F. & Koumoutsakos P. Feature point tracking and trajectory analysis for video imaging in cell biology. *J. Struct. Biol.* **151**, 182-195 (2005).
2. Chupas P. J. & Grey C. P. Surface modification of fluorinated aluminas: application of solid state NMR spectroscopy to the study of acidity and surface structure. *J. Catal.* **224**, 69-79 (2004).
3. Qiao, B. *et al.* Single-atom catalysis of CO oxidation using Pt_1/FeO_x. *Nat. Chem.* **3**, 634-641 (2011).
4. Moses-DeBusk, M. *et al.* CO oxidation on supported single Pt atoms: experimental and ab initio density functional studies of CO interaction with Pt atom on theta-Al_2O_3(010) surface. *J. Am. Chem. Soc.* **135**, 12634-12645 (2013).
5. Kistler, J. D. *et al.* A single-site platinum CO oxidation catalyst in zeolite KLTL: microscopic and spectroscopic determination of the locations of the platinum atoms. *Angew. Chem. Int. Ed.* **53**, 8904-8907 (2014).
6. Jones, J. *et al.* Thermally stable single-atom platinum-on-ceria catalysts via atom trapping. *Science* **353**, 150-154 (2016).
7. Wang, C. *et al.* Water-mediated Mars–Van Krevelen mechanism for CO oxidation on ceria-supported single-atom Pt_1 Catalyst. *ACS Catal.* **7**, 887-891 (2017).
8. Lucci, F. R. *et al.* Selective hydrogenation of 1,3-butadiene on platinum-copper alloys at the single-atom limit. *Nat. Commun.* **6**, 8550 (2015).
9. Liu, C., Yang, K., Zhao, J., Pan, Y. & Liu, D. Hydrogenation of 1,3-butadiene over Au and Pt/SiO_2-N catalysts at low temperature. *Catal. Commun.* **67**, 72-77 (2015).
10. Gao D. *et al.* Mercaptosilane-assisted synthesis of sub-nanosized Pt particles within hierarchically porous ZSM-5/SBA-15 materials and their enhanced hydrogenation properties. *Nanoscale* **7**, 10918-10924 (2015).
11. Lonergan, W. W., Xing, X., Zheng, R., Qi, S., Huang, B. & Chen, J. G. Low-temperature 1,3-butadiene hydrogenation over supported Pt/3d/γ-Al_2O_3 bimetallic catalysts. *Catal. Today* **160**, 61-69 (2011).
12. Primet, M. El Azhar, M. & Guenin, M. Influence of the support towards platinum catalysed 1,3-butadiene hydrogenation. *Appl. Catal.* **58**, 241-253 (1990).
